# Tumor-Specific Hypermethylation of Epigenetic Biomarkers, Including *SFRP1*, Predicts for Poorer Survival in Patients from the TCGA Kidney Renal Clear Cell Carcinoma (KIRC) Project

**DOI:** 10.1371/journal.pone.0085621

**Published:** 2014-01-15

**Authors:** Christopher J. Ricketts, Victoria K. Hill, W. Marston Linehan

**Affiliations:** 1 Urologic Oncology Branch, Center for Cancer Research, National Cancer Institute, National Institutes of Health, Bethesda, Maryland, United States of America; 2 Cancer Genetics Branch, National Human Genome Research Institute, National Institutes of Health, Bethesda, Maryland, United States of America; Johns Hopkins University, United States of America

## Abstract

The recent publication of the TCGA Kidney Renal Clear Cell Carcinoma (KIRC) project has provided an immense wealth and breadth of data providing an invaluable tool for confirmation and expansion upon previous observations in a large data set containing multiple data types including DNA methylation, somatic mutation, and clinical information. In clear cell renal cell carcinoma (CCRCC) many genes have been demonstrated to be epigenetically inactivated by promoter hypermethylated and in a small number of cases to be associated with clinical outcome. This study created two cohorts based on the Illumina BeadChip array used to confirm the frequency of tumor-specific hypermethylation of these published hypermethylated genes, assess the impact of somatic mutation or chromosomal loss and provide the most comprehensive assessment to date of the association of this hypermethylation with patient survival. Hypermethylation of the *Fibrillin 2* (*FBN2*) gene was the most consistent epigenetic biomarker for CCRCC across both cohorts in 40.2% or 52.5% of tumors respectively. Hypermethylation of the *secreted frizzled-related protein 1* (*SFRP1*) gene and the *basonuclin 1* (*BNC1*) gene were both statistically associated with poorer survival in both cohorts (*SFRP1* - p = <0.0001 or 0.0010 and *BNC1* - p = <0.0001 or 0.0380) and represented better independent markers of survival than tumor stage, grade or dimension in one cohort and tumor stage or dimension in the other cohort. Loss of the SFRP1 protein can potentially activate the WNT pathway and this analysis highlighted hypermethylation of several other WNT pathway regulating genes and demonstrated a poorer survival outcome for patients with somatic mutation of these genes. The success of demethylating drugs in hematological malignances and the current trials in solid tumors suggest that the identification of clinically relevant hypermethylated genes combined with therapeutic advances may improve the effectiveness and usefulness of such drugs in clear cell renal cell carcinoma.

## Introduction

Renal cell carcinoma (RCC) is diagnosed in >200,000 individuals in the world each year, accounting for ∼2% of all cancers [1]. In the U.S. it is estimated that in 2013 65,150 individuals (40,430 men and 24,720 women) will be diagnosed with this chemotherapy-resistant disease and that 13,630 individuals will die of cancer of the kidney and renal pelvis [2]. RCC is not a single uniform disease and can be classified by differing histologies and different clinical courses. The most common type (∼75% of cases) is the histologically defined clear cell renal cell carcinoma (CCRCC) that is closely associated with loss/inactivation of the von Hippel-Lindau (*VHL*) tumor suppressor gene [3]–[5]. For this reason the Cancer Genome Altas (TCGA) chose CCRCC as the first of the kidney cancer subtypes to study and publish [6]. This report re-demonstrated the importance of *VHL* mutation as well as confirming many of the recently identified commonly mutated genes in RCC, such as *PBRM1*, *SETD2* and *BAP1* [7]–[10]. The report demonstrated that tumor promoter hypermethylation was increased compared to normal kidney and increased in association with both higher stage and grade as well as demonstrating a specific hypomethylation profile in association with mutation of the *SETD2* mutation [6]. Additionally, the methylation profiles within the tumors could be used to subset the RCC patients and this differentiated for their overall survival [6]. That aberrant DNA methylation, in particular promoter hypermethylation and transcriptional silencing of tumor suppressor genes (TSGs), may play an important role in the development of renal cell carcinoma has been known for some time [11]. In CCRCC it has been shown that the *VHL* tumor suppressor gene is inactivated by promoter hypermethylation in ∼15% of cases [5], [12], [13] and that other tumor suppressor genes, such as *RASSF1*, can be commonly hypermethylated while they are rarely mutated [14], [15]. Additional to these examples there are reports of greater than 60 candidate tumor suppressor genes that demonstrate evidence of tumor-specific promoter hypermethylation in RCC than have been identified by various methodologies [11]. Traditionally, these studies used targeted gene specific analyses, such as methylation-specific PCR (MSP) and combined bisulfite restriction analysis (CoBRA), but more recently several different types of non-specific global methylation aberration assessment assays have been used to assess RCC. These include an RCC cell-line specific assay assessing gene up-regulation post 5-aza-2′-deoxycytidine demethylation treatment by gene expression microarrays [16], methylated DNA immunoprecipitation (MeDIP) analysis of tumors [17] and analyzing tumors using two different BeadChip arrays, the cancer gene specific goldengate array [18] and the global Infinium HumanMethylation27 BeadChip [19]. This wealth of investigation has provided a large number of potentially important methylated genes within sporadic RCC but very little consensus on which genes are truly important or hold true for larger populations and whether any single methylated gene or group of genes have relevance to clinical outcome. Thus, a great opportunity is provided to this field by the publication of the TCGA KIRC (Kidney Renal Clear Cell Carcinoma) data. These data include 199 CCRCC tumor/normal paired analyses using the Infinium HumanMethylation27 BeadChip and 160 CCRCC tumor/normal paired analyses using the newer and more extensive the Infinium HumanMethylation450 BeadChip. Additionally, there is somatic mutation analysis data and chromosome copy number alteration data for a large number of CCRCC tumor that can used to assess any given candidate gene for evidence of alternative methods of loss or for potentially relevant second hits to partially methylated tumor suppressor genes. The object of this manuscript is to assess a list known RCC candidate methylation genes (compiled from a recent review [11] and a recent Infinium HumanMethylation27 based publication [19]) by interrogation of the TCGA KIRC data and produce a refined list of confirmed, important genes/probes and to demonstrate whether their tumor-specific hypermethylation in tumors can predict for poorer survival in patients and thus be markers of poor prognosis kidney cancer.

## Methods

### Ethics Statement

All patient data was acquired from the published TCGA Kidney Renal Clear Cell Carcinoma (KIRC) project [6] and within this publication it is stated “Specimens were obtained from patients, with appropriate consent from institutional review boards”. This data can be accessed by going to the online open access Nature article entitled “Comprehensive molecular characterization of clear cell renal cell carcinoma” in volume 499 at pages 43–49 and selecting the supplementary information. Within this supporting information is a downloadable batch of files entitled “Supplementary Data” and within is a file entitled “Data_File_S2_clinical_dataset.xlsx that contains all the data used herein. This data is publicly available and the samples were de-identified and encoded with TCGA sample codes before publication. The TCGA data concerning genomic and clinical information is organized into two categories: one that is openly accessible to the public and one that has controlled access, available only to qualified researchers obligated to secure the data. The open access data set contains only information that is not individually unique and does not pose a risk of patient re-identification. All the data used within this manuscript was obtained from the open access data set and has passed the criteria for unrestricted publication with the following statement “No restrictions; all data available without limitations” listed at http://cancergenome.nih.gov/abouttcga/policies/publicationguidelines.

### The Cancer Genome Atlas (TCGA - http://cancergenome.nih.gov/) KIRC Data Retrieval

Data was retrieved from the Cancer Genome Atlas using the TCGA data portal to download the Infinium HumanMethylation27 BeadChip data for the 199 tumour and associated tumour normal samples for which the Infinium Methylation27 arrays had been performed in the TCGA KIRC (Kidney Renal Clear Cell Carcinoma) project. This comprised the following records: TCGA-A3-3306, 3308, 3311, 3313, 3316→3317, 3319→3320, 3322→3326, 3328→3329, 3331, 3335→3336, 3343, 3346, 3352, 3359, 3363, 3365, 3372, 3374, 3378, 3380, 3382→3383, 3347, 3349, 3351, 3362 (n = 34), TCGA-B0-4833→4834, 4836→4839, 5075, 5077, 5081, 5084→5085, 5088 (n = 12), TCGA-B2-3923→3924, 4098→4099, 4102 (n = 5), TCGA-B8-4143, 4154 (n = 2), TCGA-BP-4158→4167, 4169→4170, 4173→4174, 4176, 4325→4327, 4329→4332, 4334→4335, 4337→4338, 4340→4347, 4349, 4351→4355, 4756, 4758→4759, 4761→4769, 4771, 4774→4777, 4781, 4784, 4787, 4789→4790, 4797→4799, 4803→4804, 4807, 4959→4965, 4967→4977, 4981→4983, 4985→4989, 4991→4992, 4994→4995, 4998→5001, 5004, 5006-5009 (n = 105), TCGA-CJ-4634→4644, 4868, 4870→4876, 4878, 4881, 4884→4895, 4899→4900 (n = 35) and TCGA-CZ-4854, 4857→4858, 4860→4862 (n = 6). Data was retrieved from the Cancer Genome Atlas using the TCGA data portal to download the clinical data and Infinium HumanMethylation450 BeadChip data for the 160 tumour and associated tumour normal samples for which the Infinium Methylation27 arrays had been performed in the TCGA Kidney renal clear cell carcinoma (KIRC) project. This comprised the following records: TCGA-A3-3357, 3367, 3370, 3373, 3376, 3385 (n = 6), TCGA-B0-4688, 4690→4691, 4693→4694, 4696→4699, 4701, 4703, 4706→4707, 4710, 4712→4714, 4718, 4810→4811, 4813→4819, 4821→4824, 4823→4824, 4827→4828, 4841→4849, 4852, 4945, 5080, 5083, 5092, 5094→5100, 5102, 5104, 5106→5110, 5113, 5115→5117, 5119→5121, 5400, 5402, 5710→5713 (n = 74), TCGA-BP-4177, 4760, 4770, 4782, 4795, 4801, 4993, 5010, 5168→5170, 5173→5178, 5180→5196, 5198→5202 (n = 37), TCGA-CJ-4869, 4882, 4897, 4901→4905, 4907→4908, 4912→4913, 4916, 4918, 4920, 4923 (n = 16), TCGA-CZ-4853, 4856, 4859, 4863→4866, 5451→5470 (n = 27).

### Infinium BeadChip Data Analysis

After all the data was downloaded all the probes that represented the 77 previously published kidney cancer-associated hypermethylated gene candidates (Table S1 in File S1) were selected. In the case of the HumanMethylation450 BeadChip arrays only probes that were described by the annotation files as being within the CpG island were selected. Selected probes were then removed from analysis if the average β-value for the associated normals was greater than 0.25 to remove normally methylated genes. For the remaining probes a difference value (D-value) was calculated for each probe in each tumor by subtracting the associated normal β-value from the tumor β-value in each case. A nominal D-value of +0.35 was selected as representing significantly increased methylation, assuming that as a minimum this would represent a change from no methylation to hemi-methylation given some background level of methylation and some impurity in the tumors. Ideally, a probe would have a starting β-value of 0.00 in the associated normal and a totally pure homozygously hemi-methylated tumor would have a β-value of 0.50 and thus a D-value of +0.50, but if the probe had a starting β-value of 0.05 in the associated normal and a 80% pure hemi-methylated tumor had a β-value of ∼0.40 then a D-value of +0.35 would represent significant changes in methylation. Hypermethylation of a probe was considered significantly frequent for either cohort if it occurred in 5% or more of tumors.

### Assessment of Chromosomal Deletion/Amplification Data, Somatic Mutation Data and Clinical Information

The chromosomal deletion and amplification data and clinical information was freely available from the supplementary files provided by the TCGA KIRC project Nature publication [6]. The somatic mutation data was acquired using the cBioPortal for Cancer Genomics provided by the Memorial Sloan-Kettering Cancer Center (http://www.cbioportal.org/public-portal/) and analyzing the Kidney Renal Clear Cell Carcinoma (TCGA, in press) data set. Survival analysis for somatic mutations was performed using the inbuilt cBioPortal software.

### Statistical Analysis

The Kaplan-Meier survival curve analysis and the Cox proportional-hazard regression was performed using the MedCalc statistical software (http://www.medcalc.org/index.php). Cox proportional-hazard analysis was performed with the “Enter” method (i.e. enter all variables in the model in one single step, without checking) and in each analysis the hypermethylation of a single probe or gene was compared to tumor stage, tumor grade, maximum tumor dimension and patient gender. Correction for multiple analyses was performed using False Discovery Rate (FDR) modulation of p-values for the Kaplan-Meier survival curve analysis. All statistics were considered significant with a p-value ≤0.05.

## Results

The TCGA KIRC (Kidney Renal Clear Cell Carcinoma) project ran HumanMethylation27 bead arrays on 199 clear cell Renal Cell Carcinoma (CCRCC) tumor/normal paired samples and HumanMethylation450 bead arrays on a further 160 separate CCRCC tumor/normal paired samples, most of which were also assessed for chromosomal copy number loss/gain and somatic mutation (sample ids listed in methods). Importantly, clinical data was available for 326 of these samples. This provides a unique tool for the assessment of the previously published kidney cancer-associated hypermethylated gene candidates from which 77 genes were chosen (Table S1 in File S1).

### Tumor-specific Hypermethylation Analysis of the 199 CCRCC HumanMethylation27 BeadChip Cohort

Initial analysis was performed using the 199 CCRCC tumor/normal paired samples assessed by the HumanMethylation27 BeadChip arrays. For the purpose of this analysis only probes that were unmethylated in the normal state were of interest thus the average β-value across all the associated normals for any probe had to be less than or equal to 0.25. To assess tumor-specific hypermethylation a difference value (D-value) was calculated for each probe in each tumor by subtracting the associated normal β-value from the tumor β-value in each case. A nominal D-value of +0.35 was selected as representing significantly increased tumor-specific hypermethylation and a frequency greater than or equal to 5% was nominally considered significantly frequent.

For the initial analysis, all 287 probes pertaining to the 77 genes in the hypermethylated candidate list were selected and assessed by the above criteria (Table S2 in File S1). The HumanMethylation27 BeadChip arrays contain an average of 2 probes per gene with some selected genes represented by larger numbers of probes, the selected genes had a higher than average representation of 3.73 probes per gene. From these 287 probes, 47 probes were considered positive for hypermethylation representing 29 different genes (Table 1). Twelve genes had two or more hypermethylated probes (5 probes - *BNC1*, 3 probes *- CDKN2A, GSTP1, SFRP1*, 2 probes - *COL1A2, DKK1, ESR1, FBN2, GNB4, OVOL1,SLC34A2, TMPRSS2*) and 9 genes had probes that were hypermethylated in greater than 15% of tumors (*BNC1* - 22.1%, *COL1A2* - 16.1%, *FBN2* - 40.2% & 24.1%, *GREM1* - 16.1%, *PCDH8* - 19.1%, *SFRP1* - 16.1%, *SLC34A2* - 30.2% & 16%, *SST* - 16.6%, *TM6SF1* - 35.2%).

**Table 1:** Analysis of the Tumor-Specific Probe Hypermethylation in the Candidate Genes for the 199 TCGA RCC Tumor/Normal Pairs Present in the HumanMethylation27 BeadChip Cohort.

	Gene Symbol	Infinium Probe ID	Percentage of 199 Tumors with Probe Difference Values ≥0.35	Average Associated Normal β-Value
**1**	BMP4	cg14310034	8.0%	0.08
**2**	BNC1	cg18952647	22.1%	0.10
	BNC1	cg19988449	11.6%	0.12
	BNC1	cg15736165	11.6%	0.21
	BNC1	cg10398682	11.1%	0.23
	BNC1	cg17051321	6.5%	0.11
**3**	CCDC8	cg15984661	5.5%	0.15
**4**	CDH1	cg24765079	5.5%	0.20
**5**	CDKN2A	cg09099744	11.1%	0.09
	CDKN2A	cg10895543	10.1%	0.15
	CDKN2A	cg07752420	9.0%	0.13
**6**	COL1A2	cg18511007	16.1%	0.17
	COL1A2	cg25300386	12.1%	0.19
**7**	DKK1	cg07684796	8.5%	0.11
	DKK1	cg12621514	7.0%	0.17
**8**	DLEC1	cg23881725	12.1%	0.20
**9**	ESR1	cg20253551	5.0%	0.13
	ESR1	cg02720618	5.0%	0.14
**10**	FBN2	cg27223047	40.2%	0.13
	FBN2	cg25084878	24.1%	0.24
**11**	GNB4	cg17483510	9.0%	0.19
	GNB4	cg09997760	5.5%	0.04
**12**	GREM1	cg21296230	16.1%	0.09
**13**	GSTP1	cg04920951	13.6%	0.10
	GSTP1	cg02659086	5.0%	0.06
	GSTP1	cg09038676	5.0%	0.18
**14**	GUCY2D	cg25465406	8.0%	0.10
**15**	HOXB13	cg15786837	6.5%	0.09
**16**	LSAMP	cg14294758	8.0%	0.10
**17**	OVOL1	cg20909686	14.1%	0.18
	OVOL1	cg13496736	11.1%	0.06
**18**	PCDH8	cg20366906	19.1%	0.14
**19**	RPRM	cg27420236	9.0%	0.08
**20**	SCUBE3	cg21604042	13.1%	0.07
**21**	SFRP1	cg22418909	16.1%	0.08
	SFRP1	cg13398291	11.1%	0.09
	SFRP1	cg15839448	7.0%	0.05
**22**	SFRP2	cg23207990	9.0%	0.07
**23**	SLC34A2	cg19616230	30.2%	0.07
	SLC34A2	cg21200703	16.1%	0.17
**24**	SST	cg02164046	16.6%	0.08
**25**	TM6SF1	cg14696396	35.2%	0.07
**26**	TMPRSS2	cg24901042	12.1%	0.17
	TMPRSS2	cg02613803	5.0%	0.06
**27**	UCHL1	cg24715245	7.0%	0.11
**28**	VHL	cg22782492	6.0%	0.10
**29**	WIF1	cg19427610	8.0%	0.05

**Figure 1:**
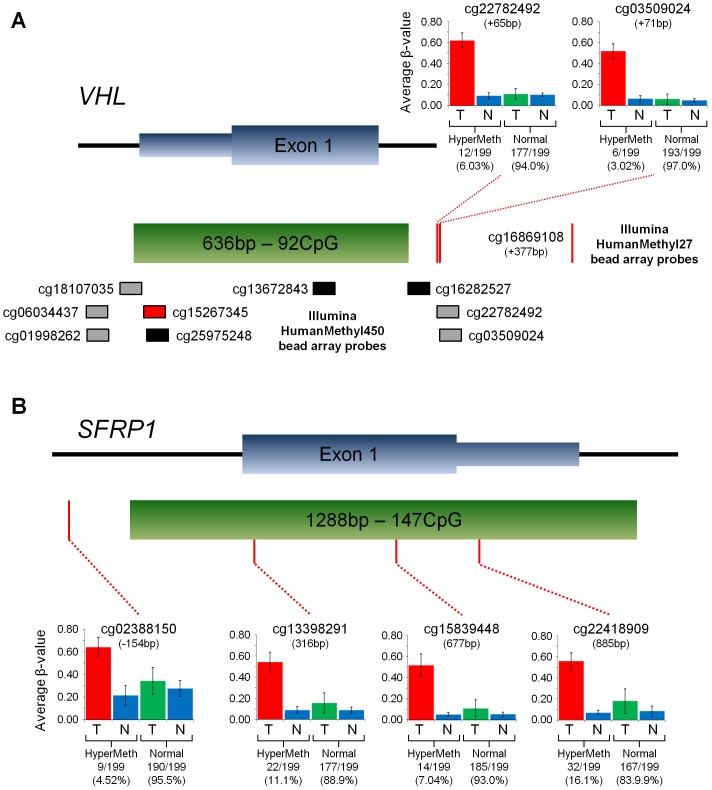
Gene Maps of the CpG Islands for the *VHL* and *SFRP1* genes demonstrating the Positioning of Probes and the Levels of Hypermethylation. This demonstrates the first exons of either the *VHL* gene (A) or the *SFRP1* gene (B) with the predicted CpG island region (green box), the position and methylation levels of the HumanMethylation27 BeadChip array probes (red lines) and, for *VHL* only, the position of the HumanMethylation450 BeadChip array probes (Black, grey and red boxes). The graphs for the HumanMethylation27 BeadChip array probes split the samples into two groups; those with tumor-specific hypermethylation (by the criteria described within) and those without. The average -values for the tumors and associated normals were graphed for each group and the standard deviation used to produce error bars. The boxes representing the HumanMethylation450 BeadChip array probes were colored black if their annotation designated them within the CpG island and grey if their annotation designated them outside of the CpG island. The red box represents the single probe that was both hypermethylated and designated within the CpG island.

Tumor-specific methylation of the *VHL* gene is an expected event and was observed in a single probe, but with a frequency slightly below the expected levels of hypermethylation. Further investigation of this highlighted a potential issue with the HumanMethylation27 BeadChip array that the probes may not be positioned within the predicted CpG island. In fact, for the *VHL* gene all 7 of the probes associated with *VHL* occur downstream of the CpG with only the closest probe demonstrating frequent tumor-specific hypermethylation (Figure 1A). In contrast, another commonly reported CCRCC hypermethylated gene, *SFRP1*, has 3 of its 5 associated probes within the predicted CpG island and all three demonstrate frequent tumor-specific hypermethylation (Figure 1B). This suggests that probe position can affect the analysis and that assessment of the CpG island probes may provide more relevant results and that the HumanMethylation450 BeadChip arrays may provide more substantial results as there are a larger number of probes than fall within the CpG islands as demonstrated with the *VHL* CpG island (Figure 1A). It is notable that the *BNC1* gene has 7 associated probes, similar to VHL, but 5 of these are within the predicted CpG island and has the largest number of frequently hypermethylated probes (Figure S1 in File S1). Interestingly, only four of the five *BNC1* CpG island probes are frequently hypermethylated and the remaining probe is positioned between other hypermethylated probes, possibly indicating variation can be present across the CpG island.

### Tumor-specific Hypermethylation Analysis of the 160 CCRCC HumanMethylation450 BeadChip Cohort

The analysis of the HumanMethylation450 BeadChip cohort was slightly modified to limit probe selection to those that described as being within the predicted CpG islands. This identified 652 probes with an average associated normal β-value ≤0.25, of which 182 probes representing 45 genes demonstrated tumor-specific hypermethylation in ≥5% of tumors (Table S3 in File S1). For each of these 45 genes the most frequently hypermethylated probe was assessed as well as the average frequency for all hypermethylated probes for genes with multiple hypermethylated probes (Table 2). These 45 genes were represented by an average of 9.2 CpG island probes per gene with a range of 1 to 25 probes. The percentage of these CpG island probes that demonstrated frequent hypermethylated in any of the 45 genes ranged from 6.3% (1 out of 16 probes in *RASSF5*) to 100% (10 out of 10 probes with *FBN2*) (Table S4 in File S1). Thirteen genes demonstrated a single probe hypermethylated in greater than 20% of tumors (*ATP5G2* - 35.6%, *DKK1* - 24.4%, *DKK2* - 26.9%, *FBN2* - 52.5%, *GUCY2D* - 28.1%, *PCDH8* - 62.5%, *PDLIM4* - 31.9%, *ROBO1* - 27.5%, *SFRP1* - 33.8%, *SLC34A2* - 26.9%, *SST* - 24.4%, *TM6SF1* - 36.9%, *ZSCAN18* - 55.0%) and furthermore, eight of these genes (underlined) had multiple hypermethylated probes with an average hypermethylation frequency in tumors of greater than 20% (Table 2).

**Table 2:** Analysis of the Tumor-Specific Probe Hypermethylation in the Candidate Genes for the 160 TCGA RCC Tumor/Normal Pairs Present in the HumanMethylation450 BeadChip Cohort.

	Gene Symbol	Percentage of Diff. Values ≥0.35 in Tumors for the Most Hypermethylated Gene-Associated Probe (Infinium Probe ID)	No. of Probes Methylated in >5% of tumors out of all the Gene-Associated CpG Island Probes	Average Percentage of Diff. Values ≥0.35 in Tumors for all Positive Gene-Associated Probes
**1**	ATP5G2	35.63% (cg13691247)	4 out of 10	26.70%
**2**	BMP4	8.13% (cg08162372)	2 out of 14	7.50%
**3**	BNC1	18.75% (cg06523224)	13 out of 20	10.20%
**4**	BTG3	5.00% (cg02652260)	1 out of 9	n/a
**5**	CCDC8	8.75% (cg06747432)	1 out of 1	n/a
**6**	CDH13	7.50% (cg08747377)	2 out of 7	7.20%
**7**	COL14A1	6.88% (cg04242021)	1 out of 6	n/a
**8**	CORO6	5.00% (cg04568355)	1 out of 7	n/a
**9**	CST6	10.00% (cg04327181)	1 out of 5	n/a
**10**	DKK1	24.38% (cg08812555)	5 out of 7	14.00%
**11**	DKK2	26.88% (cg13139972)	3 out of 5	12.70%
**12**	DKK3	6.88% (cg19867649)	3 out of 13	5.80%
**13**	DLEC1	18.13% (cg20684180)	3 out of 6	10.80%
**14**	EPB41L3	18.75% (cg07352438)	11 out of 13	10.50%
**15**	FBN2	52.50% (cg27223047)	10 out of 10	23.80%
**16**	GATA5	13.13% (cg09339194)	14 out of 25	7.10%
**17**	GNB4	15.00% (cg12872693)	4 out of 7	11.30%
**18**	GREM1	13.75% (cg21296230)	3 out of 7	10.20%
**19**	GSTP1	12.50% (cg04920951)	4 out of 5	11.60%
**20**	GUCY2D	28.13% (cg04157161)	2 out of 8	20.90%
**21**	HOXC13	7.50% (cg07892422)	2 out of 9	6.30%
**22**	KLHL35	15.00% (cg12001148)	2 out of 7	10.00%
**23**	KRT19	8.13% (cg11462865)	2 out of 3	7.50%
**24**	LOXL1	6.25% (cg22590761)	1 out of 11	n/a
**25**	MGMT	7.50% (cg02022136)	1 out of 14	n/a
**26**	OVOL1	18.75% (cg20909686)	2 out of 11	14.40%
**27**	PCDH8	62.50% (cg05336395)	11 out of 13	27.20%
**28**	PDLIM4	31.88% (cg02033258)	2 out of 2	23.10%
**29**	PTGS2	6.25% (cg13986130)	1 out of 10	n/a
**30**	QPCT	15.00% (cg08786077)	5 out of 8	12.10%
**31**	RASSF5	9.38% (cg18328206)	1 out of 16	n/a
**32**	ROBO1	27.50% (cg21865845)	3 out of 7	16.50%
**33**	RPRM	16.88% (cg26649384)	8 out of 12	10.30%
**34**	SCUBE3	6.25% (cg00347904)	1 out of 6	n/a
**35**	SFRP1	33.75% (cg24319902)	6 out of 8	16.90%
**36**	SFRP2	18.75% (cg22178613)	8 out of 19	8.60%
**37**	SLC34A2	26.88% (cg27513574)	7 out of 7	22.10%
**38**	SLIT2	5.00% (cg13078140)	2 out of 12	5.00%
**39**	SST	24.38% (cg02164046)	3 out of 3	17.30%
**40**	TM6SF1	36.88% (cg26460092)	5 out of 7	24.80%
**41**	TMPRSS2	18.75% (cg16084872)	4 out of 10	12.00%
**42**	UCHL1	11.88% (cg070687560	5 out of 7	9.30%
**43**	VHL	5.00% (cg15267345)	1 out of 3	n/a
**44**	WIF1	17.50% (cg26397188)	4 out of 6	15.20%
**45**	ZSCAN18	55.00% (cg14231297)	7 out of 18	24.40%

An important use of the HumanMethylation450 BeadChip cohort was to confirm the results of the HumanMethylation27 BeadChip cohort. Thus, there were 23 genes that were frequently hypermethylated (≥5%) in both cohorts (Figure S2 in File S1) and 7 genes that were hypermethylated in ≥15% of tumors for both cohorts, *BNC1*, *FBN2*, *PCDH8*, *SFRP1*, *SLC34A2*, *SST*, *TM6SF1* (Tables 1 & 2), that represent good potential epigenetic biomarkers for CCRCC.

### Assessment of Chromosomal Loss/Deletion and Somatic Mutation of the Hypermethylated Genes in the TCGA Project Tumors

**Figure 2:**
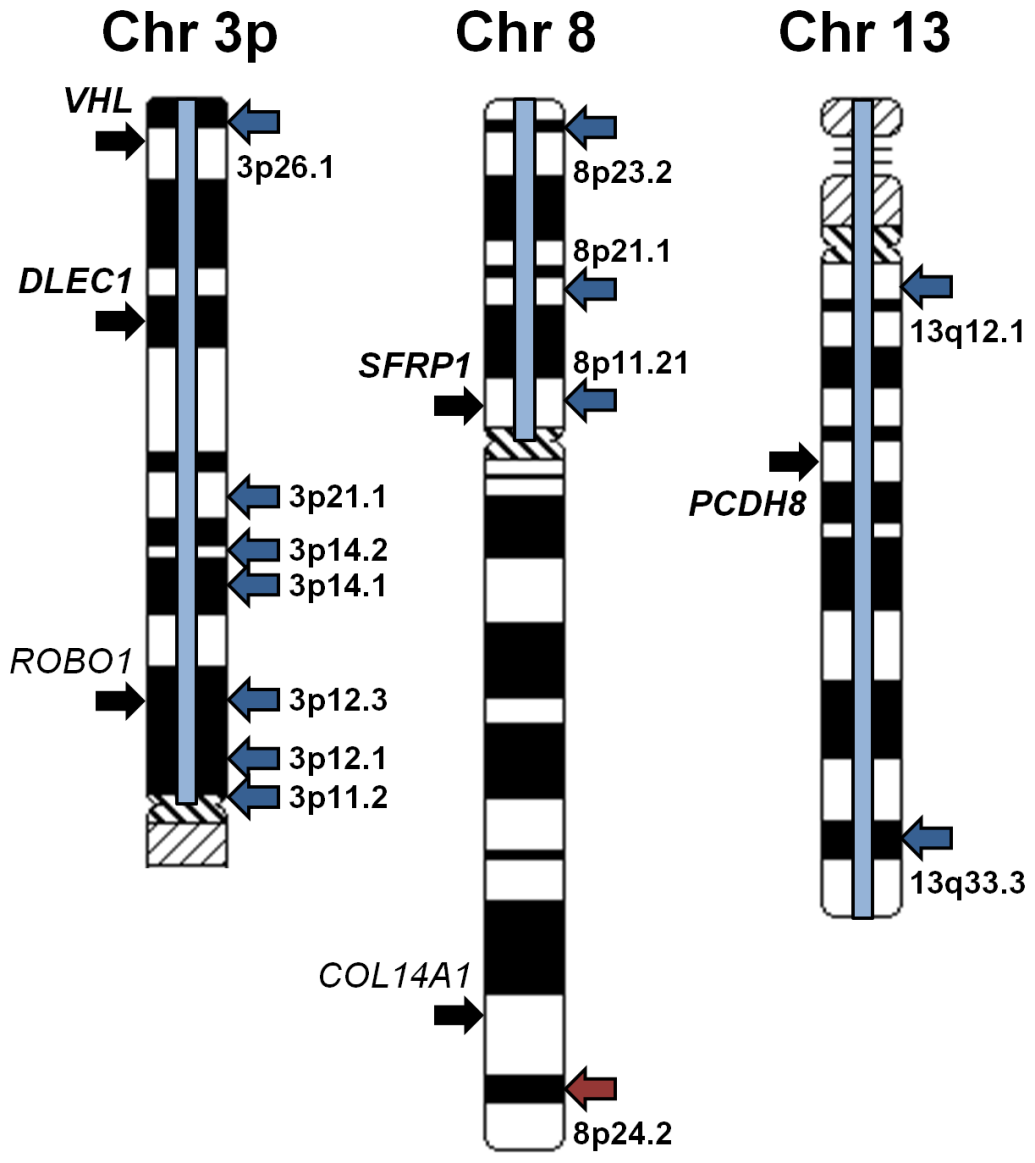
TCGA KIRC hypermethylated genes that occur in regions of common whole/partial chromosomal or focal loss/deletion. The regions of chromosomal variation identified from the published TCGA Kidney Renal Clear Cell Carcinoma (KIRC) project data for chromosomes 3p, 8 and 13 were mapped with the regions of whole/partial chromosomal loss/deletion highlighted with blue bars and regions of focal loss or gain designated with blue or red arrows respectively. Genes that demonstrated tumor-specific hypermethylation in either cohort were designated with black arrows and those genes that were present in both cohorts were highlighted in bold.

The published TCGA KIRC project data includes information on common regions of chromosomal deletion and amplification, at both the whole/partial chromosome and focal level, as well as data on somatic mutation in the tumors. This data was assessed on the assumption that frequent chromosomal deletion or somatic mutation could either occur in conjunction with hypermethylation, possibly as a “second hit”, or as an alternative method of gene silencing and thus refine the list of hypermethylated genes to those of greater potential importance. Currently not all TCGA KIRC project samples have been analyzed for all these different data types and so the data does not exactly match the specific 359 CCRCC tumors that were analyzed for methylation but in both cases represents data from over 400 CCRCC tumors. The 51 genes that demonstrated hypermethylated probes in either of the two BeadChip cohorts were mapped onto the human chromosomes, with those genes hypermethylated in both cohorts highlighted in bold (Figure S3 in File S1). Added to this were the regions of whole/partial chromosomal loss/deletion (with blue bars) or amplification (with red bars) and regions of focal loss (blue arrows) or gain (red arrows) (Figure S3 in File S1). This identified four genes that mapped to regions of chromosomal and focal loss, *VHL* (Chr.3p), *ROBO1* (Chr.3p), *SFRP1* (Chr.8p) and *CDKN2A* (Chr.9p), as well as a further three genes that mapped to regions of chromosomal loss, *DLEC1* (Chr.3p), ESR1 (Chr.6q), PCDH8 (Chr.13q) (Figure 2, Figure S3 in File S1).

Somatic mutation was a rare event for any of the hypermethylated genes with the exception of the VHL gene that demonstrated the expected high level of mutation with 235 out of 424 (55.4%) tumors having detectable mutation. A small number of genes demonstrated low levels of somatic mutation with a frequency of approximately 1% or greater; *BNC1* (0.9%), *DLEC1* (0.9%), *PTGS2* (0.9%), *CDKN2A* (1.2%), *COL1A2* (1.4%) and *FBN2* (3.1%) (Figure S4 in File S1).

### Survival Analysis of the Hypermethylated Genes in the TCGA Project Patients

The published TCGA KIRC project data includes clinical information for a large number of the patients from which the CCRCC tumors were excised. Complete clinical data, including gender, tumor size, tumor grade, tumor stage, patient survival status and duration from surgery to last check-up or death, was available for 181 patients from the HumanMethylation27 BeadChip cohort (Table S5 in File S1) and 145 patients from the HumanMethylation450 BeadChip cohort (Table S6 in File S1). In all cases the presence of tumor-specific hypermethylation was compared to the duration in days of patient survival (recurrence was not used as a factor) using Kaplan-Meier survival curves and selected probes/genes were further assessed by Cox proportional-hazards regression to see if the hypermethylation had any significant value as an independent marker for poorer survival in comparison to gender, maximum tumor dimension, tumor grade and tumor stage.

**Figure 3:**
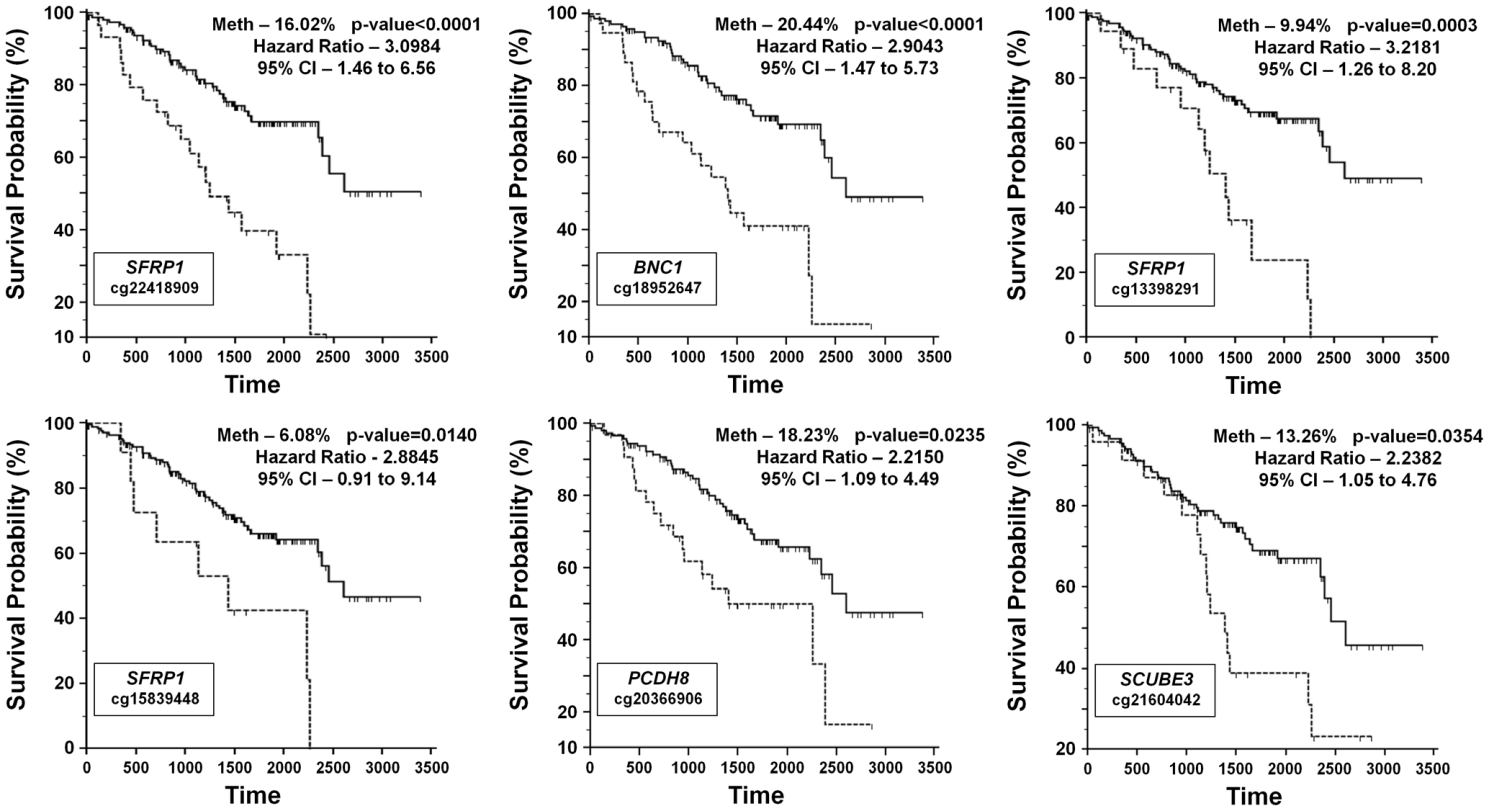
Kaplan-Meier survival curves for the hypermethylated probes from the HumanMethylation27 BeadChip cohort. Kaplan-Meier survival curves were calculated for all of the tumor-specific hypermethylated probes within the HumanMethylation27 BeadChip cohort and these graphs represent the probes for which the p-value remained significant after false discovery rate (FDR) correction for multiple analyses. The percentage of hypermethylation (Meth) is representative of the 181 CCRCC tumor/normal pairs for which clinical information was available. The relevant BeadChip probe is listed with the gene name.

For HumanMethylation27 BeadChip cohort this analysis was performed considering each hypermethylated probe separately and hypermethylation of 15 out of the 48 probes were demonstrated to predict for worse survival (Table 3). To correct for false positives due to multiple analyses a false discovery rate (FDR) modification was applied and 6 probes representing 4 genes, *SFRP1 (3x)*, *BNC1*, *PCDH8* and *SCUBE3* remained statically significant (Figure 3). Cox proportional-hazards regression for these 6 probes demonstrated that 3 probes represented the most statistically significant indicators of survival compared to tumor stage, grade, maximum dimension or patient gender; *SFRP1-*cg13398291 (p = 0.009, HR = 2.40, CI = 1.25–4.64), *BNC1* (p = 0.004, HR = 2.36, CI = 1.32–4.20) and *PCDH8* (p = 0.012, HR = 2.13, CI = 1.18–3.82) (Table S7 in File S1). The remaining 3 probes were all statistically significant indicators, but were less significant than tumor stage (Table S7 in File S1).

**Table 3:** Kaplan Meier Survival Analysis for the HumanMethylation27 BeadChip Cohort.

HumanMethylation27 BeadChip Cohort			Survival Analysis - Kaplan-Meier Analysis*			
Gene Name	HyperMeth. Probe	HyperMethylation (% of 181 tumors)	p-value	Hazard Ratio	95% Confidence Interval	False Discovery Rate corrected p-value
BNC1	cg18952647	20.44%	<0.0001	2.9043	1.4731 to 5.7260	<0.0001
SFRP1	cg22418909	16.02%	<0.0001	3.0984	1.4628 to 6.5630	<0.0001
SFRP1	cg13398291	9.94%	0.0001	3.2181	1.2628 to 8.2007	0.0003
SFRP1	cg15839448	6.08%	0.0035	2.8845	0.9106 to 9.1371	0.0140
PCDH8	cg20366906	18.23%	0.0047	2.2150	1.0929 to 4.4891	0.0235
SCUBE3	cg21604042	13.26%	0.0059	2.2382	1.0534 to 4.7553	0.0354
UCHL1	cg24715245	5.52%	0.0087	2.7507	0.8208 to 9.2179	0.0609
TM6SF1	cg14696396	35.36%	0.0089	1.9721	1.1484 to 3.3867	0.0712
GNB4	cg09997760	4.97%	0.0170	2.6824	0.7354 to 9.7838	0.1530
BNC1	cg10398682	9.94%	0.0213	2.1786	0.8753 to 5.4223	0.2130
OVOL1	cg13496736	9.94%	0.0245	2.1399	0.8655 to 5.2905	0.2695
BMP4	cg14310034	6.63%	0.0336	2.2939	0.7522 to 6.9951	0.4032
SFRP2	cg23207990	8.84%	0.0427	2.0543	0.8043 to 5.2472	0.5551
RPRM	cg27420236	8.29%	0.0436	2.1148	0.7730 to 5.7858	0.6104
WIF1	cg19427610	7.73%	0.0436	2.1156	0.7731 to 5.7889	0.6540

181 patients from the HumanMethylation27 BeadChip Cohort had the appropriate clinical data.

To confirm these predictions similar analysis was performed upon the HumanMethylation450 BeadChip cohort. When the most hypermethylated probe for each gene was analyzed 12 out of the 45 probes were demonstrated to predict for worse survival with 5 genes still remaining statically significant after correction for multiple analyses (Figure S5 and Table S8 in File S1). By Cox proportional-hazards regression, two probes were statistically significant indicators, but were both less significant than tumor grade; *BNC1* (p = 0.050, HR = 1.76, CI = 1.00–3.08) and *ZSCAN18* (p = 0.028, HR = 1.87, CI = 1.07–3.24) (Table S9 in File S1). This strengthened the potential importance of *BNC1* hypermethylation as a indicator for poorer patient survival.

**Figure 4:**
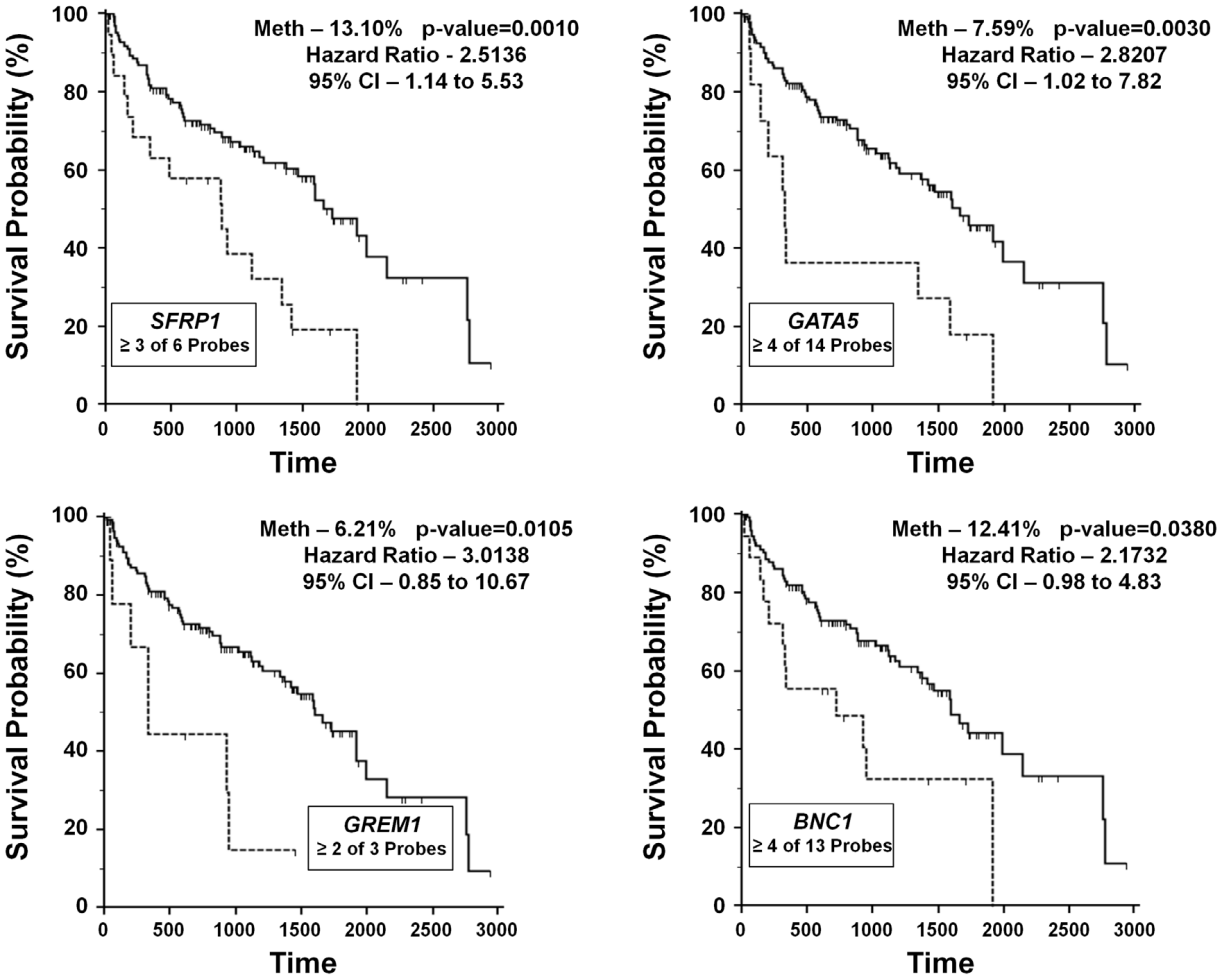
Kaplan-Meier survival curves for the hypermethylated genes from the HumanMethylation450 BeadChip cohort. Kaplan-Meier survival curves were calculated for each of the tumor-specific hypermethylated genes within the HumanMethylation450 BeadChip cohort and these graphs represent the genes for which the p-value remained significant after false discovery rate (FDR) correction for multiple analyses. The percentage of hypermethylation (Meth) is representative of the 145 CCRCC tumor/normal pairs for which clinical information was available. For each gene, the ratio of hypermethylated CpG island probes needed per tumor for it to be considered positive is listed with the gene name.

Due to the increased number probes present in the HumanMethylation450 BeadChip array, the analysis was repeated for genes with multiple hypermethylated probes such that a proportion of the probes had to be hypermethylated in each tumor before that tumor was considered positive for hypermethylation. The proportions were assigned such that genes with 2–4 hypermethylated probes needed a minimum of 2 per tumor, genes with 5–6 hypermethylated probes needed a minimum of 3 per tumor and genes with 7 or more hypermethylated probes needed a minimum of 4 per tumor. This was performed assuming the results might be more comparable to other studies where the methodologies always assess multiple CpGs and might provide a more accurate view of the CpG island methylation. Subsequently, 11 out of the 26 hypermethylated genes were demonstrated to predict for worse survival with 4 genes, *SFRP1*, *GATA5*, *GREM1* and *BNC1*, still remaining statically significant after correction for multiple analyses (Table 4, Figure 4). Cox proportional-hazards regression for hypermethylation of these 4 genes demonstrated that 3 genes represented statistically significant indicators of survival, but were all less significant than tumor stage; *SFRP1* (p = 0.036, HR = 1.92, CI = 1.05–3.50), *GREM1* (p = 0.007, HR = 3.05, CI = 1.36–6.86) and *BNC1* (p = 0.033, HR = 1.99, CI = 1.06–3.75) (Table S9 in File S1). This data demonstrated that hypermethylation of either *SFRP1* or *BNC1* were independent markers for poor patient survival in both cohorts.

**Table 4:** Kaplan Meier Survival Analysis for the HumanMethylation450 BeadChip Cohort.

HumanMethylation450 BeadChip Cohort			Survival Analysis - Kaplan-Meier Analysis*			
Gene Name	HyperMeth. Probes required per gene	HyperMethylation (% of 145 tumors)	p-value	Hazard Ratio	95% Confidence Interval	False Discovery Rate corrected p-value
SFRP1	≥3 out of 6	13.10%	0.0010	2.5136	1.1429 to 5.5278	0.0010
GATA5	≥4 out of 14	7.59%	0.0015	2.8207	1.0170 to 7.8236	0.0030
GREM1	≥2 out of 3	6.21%	0.0035	3.0138	0.8511 to 10.672	0.0105
BNC1	≥4 out of 13	12.41%	0.0095	2.1732	0.9774 to 4.8323	0.0380
DKK2	≥2 out of 3	8.97%	0.0127	2.2806	0.9000 to 5.7789	0.0635
FBN2	≥4 out of 10	28.28%	0.0171	1.8109	1.0204 to 3.2139	0.1026
WIF1	≥2 out of 4	16.55%	0.0184	1.9052	0.9772 to 3.7144	0.1288
TM6SF1	≥3 out of 5	26.21%	0.0202	1.7914	1.0111 to 3.1738	0.1616
GUCY2D	Both	5.52%	0.0207	2.5730	0.7214 to 9.1773	0.1863
GNB4	≥2 out of 4	13.79%	0.0211	2.0013	0.9236 to 4.3366	0.2110
RPRM	≥4 out of 8	6.90%	0.0219	2.3031	0.8100 to 6.5488	0.2409

145 patients from the HumanMethylation450 BeadChip Cohort had the appropriate clinical data.

## Discussion

Clear cell renal cell carcinoma (CCRCC) has been shown to associate with a wide spectrum of tumor-specific genetic and epigenetic alterations that can result in loss or inactivation of numerous tumor suppressor genes (TSGs). The investigation and identification of which TSG losses are most associated with tumor progression or patient survival should therefore aid in the management of this therapeutically resistant disease. CCRCC has a very strong association with the tumor-specific loss of chromosome 3p and mutation/inactivation, including promoter hypermethylation, of the *VHL* gene on the remaining chromosome. In fact this association with chromosome 3p loss has recently been strengthened by several publications demonstrating that 4 out of five (*VHL*, *PBRM1*, *SETD2* and *BAP1*) of the most frequently mutated genes associated with CCRCC are on chromosome 3p [6]–[10] and that the mutation of either *SETD2*, *BAP1* or *PBRM1* associates with poorer prognosis or progression in those patients [6], [20], [21].

Although these comprehensive analyses have demonstrated and reinforced the importance of these 3p genes, there are still only a small number of frequently (>5%) mutated genes associated with CCRCC. In contrast, a far larger number of genes have been demonstrated to have tumor-specific CpG island hypermethylation in CCRCC and other histological sub-types of kidney cancer, including several genes that demonstrate promoter hypermethylation in greater than 20% of cases [11]. Unlike sequence analysis, where a relatively uniform method of assessment is used, methylation analysis has been performed by various different methods that may not be comparable and largely on relatively small numbers of samples. Many methodologies only assess a small number of CpGs per CpG island that are expected to be representative of the methylation levels of the entire CpG island. For Methylation-Specific PCR (MSP) it is limited to those CpGs within the primers, while in combined bisulfite restriction analysis (CoBRA) by the number of CpGs within the relevant restriction cut sites. More comprehensive analysis of CpG islands can be achieved by bisulfite sequencing or pyrosequencing, but these methodologies are very time consuming and allow for analysis of only a small fragment of the genome each time. Thus confirmation of tumor-specific methylation in novel large cohorts by alternative methodologies is very valuable in demonstrating the importance of the hypermethylation in any given gene.

The TCGA KIRC (Kidney Renal Clear Cell Carcinoma) project data provides a large amount of raw and processed data including analysis of methylation using the BeadChip array technology. This technology uses probes that investigate only a single CpG per probe, which is obviously restrictive, but the number of probes investigated is only limited by the current state of the technology. Thus during the time the TCGA KIRC data was being gathered early samples were analyzed by an initial array (HumanMethylation27) containing ∼27,000 probes, but later samples used a newer, larger array (HumanMethylation450) containing ∼450,000 probes that provided a greater intensity to the analysis. To date, no other CCRCC samples have been assessed by the array HumanMethylation27 BeadChip array and only a single study, from which we obtained some of the candidate hypermethylated genes, has used the array HumanMethylation27 BeadChip array [19]. This provides a large amount of data for assessment and importantly both the tumor and associated normals were assessed allowing for the calculation of tumor-specific gain of hypermethylation (tumor β-value – normal β-value) that should hopefully control for the fact that CpG island methylation levels can increase with age in normal tissue [22].

By splitting the patients into two cohorts based on the use of the two different BeadChip arrays we could both look confirmatory hypermethylation of genes occurring in both cohorts and compare the usefulness of each array. This highlighted one of the known weaknesses with the original HumanMethylation27 BeadChip array that most genes are only represented by a few probes and that the positioning of those probes in respect to the CpG island may affect the result of the analysis. This could easily result in the underestimation or complete lack of assessment of methylation for a gene. This is exemplified by comparison of the position of the probes associated with the *VHL* gene and the *SFRP1* gene in the HumanMethylation27 BeadChip array and demonstrates the advantages of using the HumanMethylation450 BeadChip array. The increased number of probes within the HumanMethylation450 BeadChip array allowed for the restriction of probe selection to those within the CpG island and for the assessment of genes based on the presence of multiple hypermethylated probes. This approach seems justified as the survival analysis using genes with multiple probes produced 4 statistically significant genes all of which have been previously associated with poor patient survival and tumor progression. Two recent publications demonstrated that GATA5 hypermethylation in RCC associated with status of metastasis, progressive disease and shortened progression-free survival [23] and that GREM1 hypermethylation in CCRCC.associated with increased Fuhrman grade and decreased overall survival [24]. While the remaining two genes, BNC1 and SFRP1, are discussed in greater length below.

Due to the potential limitations of using either array we considered genes that showed frequent methylation in both cohorts to have demonstrated strong evidence of tumor specific methylation in CCRCC and those for which only one cohort showed frequent methylation to have demonstrated weaker strong evidence of tumor specific methylation in CCRCC. These genes that were only identified by one platform included well known tumor suppressors such as *CDKN2A* and *RASSF5* that obviously warrant investigation, but did not meet the criteria for this specific study. These limitations are particularly relevant to the HumanMethylation27 BeadChip arrays and subsequent studies are likely to use the newer platforms, but the currently available data is still important and useful. Thus, data was not used to discredit or disagree with any previously published data concerning a genes methylation frequency as we could not be certain that this methodology accurately assessed all target genes.

This analysis was successful in confirming a large number of the predicted hypermethylated genes within the TCGA KIRC data, but one of the purposes of using this data was to ingrate in the other analyses, such as chromosomal loss or somatic mutation, to highlight particularly important genes and to use the clinical information to attempt to identify epigenetic markers of poorer patient survival. From this analysis 4 genes (*FBN2*, *PCDH8*, *BNC1* and *SFRP1*) stand out as either particularly strong epigenetic biomarkers of kidney cancer or as significant predictors of patient outcome. One form of analysis that was not performed due to a lack of data was the assessment of correlation between promoter hypermethylation and mRNA expression. Although a large number of the tumor samples had been analyzed for mRNA expression by RNASeq analysis, only a small number of tumor/normal pairs had been assessed (n = 69) and of those only a third (n = 22) had been assessed for methylation. For these selected 4 genes the mRNA expression levels were compared between the methylated tumors vs the non-methylated tumors within the HumanMethylation450 BeadChip cohort and demonstrated decreased expression within the methylated tumors (Figure S6 in File S1). That the *PCDH8* and *SFRP1* genes demonstrated mild decreases could potentially be a result of the unmethylated samples having the common chromosomal losses and thus also having low expression compared to normal kidney. Due to the lack of normal controls, this data was considered supportive but not substantial enough to draw conclusions from.

The *Fibrillin 2* (*FBN2*) gene was very frequently hypermethylated in tumors from both cohorts with a single probe being hypermethylated in 40.2% of the HumanMethylation27 BeadChip cohort and 52.5% of the HumanMethylation450 BeadChip cohort and the gene demonstrated a relatively high somatic mutation rate (3.1%), although the effects of these mutations are yet to be elucidated. Hypermethylation of *FBN2* has been previously reported in RCC (34%) [17] as well as several other cancer types including small cell lung cancer [25], esophageal squamous cell carcinoma [26] and colorectal cancer, in which it was potentially associated with hepatic metastasis [27]. Currently, the function of FBN2 protein is thought to be similar to the related FBN1 protein, they are both key components of human microfibrils that may affect regulation of TGF-β signaling. Loss of FBN1 has been demonstrated to result in excess activation of TGF-β [28] and this may also be true for FBN2 loss and aid malignancy by dysregulating the TGF-β signaling pathway. Although, hypermethylation of FBN2 demonstrated no correlation with patient survival it presents as potential biomarker for CCRCC.

The *protocadherin 8* (*PCDH8*) gene was hypermethylated in both cohorts, with one cohort demonstrating hypermethylation in >20% of tumors, and resides on the frequently lost chromosome 13q region. *PCDH8* hypermethylation correlated with poorer patient survival in one cohort where it represented a more significant prognostic marker for patient survival than tumor stage, grade or dimension, but was not statically significant in the second cohort, although a trend was present. Hypermethylation of *PCDH8* has been previously reported in RCC (58%) [17] as well as in mantle cell lymphoma [29], breast carcinoma [30] and gastric cancer, including para-carcinomic but not normal gastric tissue [31]. In gastric cancer, *PCDH8* hypermethylation was associated with lymph node metastasis that could be indicative poorer survival outcome [31]. In these previous reports it was shown that either re-introduction of PCDH8 or treatment with the 5-aza-2′-deoxycytidine demethylating agent resulted in suppression of migration and promotion of apoptosis [30], [31]. Although the 5-aza-2′-deoxycytidine treatment was non-specific, thus the activation of other hypermethylated tumor suppressors could be producing the effect, the re-introduction of PCDH8 demonstrates the specific importance of this gene. Cadherin molecules are known to be important for creating and maintaining proper tissue architecture and development and mutation of classic cadherins, such as E-cadherin, in several cancer types have shown them to function as tumor suppressors [32]. The exact function of PCDH8 protein is unknown, but there is increasing evidence for the importance of this family of proteins as potential tumor suppressors possibly via disruption of cell-cell communication necessary for tissue organization. Within the same gene cluster as *PCDH8* there resides *PCDH17* and *PCDH20* that are both hypermethylated and homozygously deleted in esophageal squamous cell carcinoma [33] and lung cancer [34] respectively. Thus, *PCDH8* hypermethylation presents as a good biomarker for CCRCC as well as a potential marker for poorer survival in patients and the initial evidence that re-expression of *PCDH8*, possibly via 5-aza-2′-deoxycytidine treatment, has a negative effect on tumors could be therapeutically beneficial in improving these patients' survival outcomes.

The *basonuclin 1* (*BNC1*) gene was hypermethylated in both cohorts with one cohort demonstrating hypermethylation in >20% of tumors and a small proportion of tumors demonstrated somatic mutation (0.9%). More importantly, *BNC1* hypermethylation correlated with poorer patient survival in the HumanMethylation27 BeadChip cohort, where it represented a more significant prognostic marker for patient survival than tumor stage, grade or dimension, and in the HumanMethylation450 BeadChip cohort, where it was the only remaining statistically significant marker after tumor grade. Tumor-specific *BNC1* hypermethylation has been previously reported in RCC (46%) [16] as well as in prostate, breast, lung and colon cancers [35]. It has also been reported in hematological malignances including lymphoblastic [36] and lymphocytic leukemia [37]. The previous study in RCC also associated *BNC1* hypermethylation with poorer patient prognosis independent of tumor stage, grade or dimension [16]. *BNC1* encodes a transcription factor that is important in the regulation of keratinocyte differentiation and why the loss this transcription factor would be advantageous to tumor growth or progression is yet to be elucidated. A recent publication demonstrated *BNC1* to be a direct transcriptional target for the tumor suppressor gene *p63*, thus its loss could affect the tumor suppressing effect of *p63*, but in this publication the expression of *BNC1* and *p63* were elevated in the squamous cell carcinomas that were studied [38]. In spite of the lack of knowledge about its function, *BNC1* hypermethylation presents as a good biomarker for CCRCC and an excellent marker for poor patient survival. This data confirms the previous observation of this using a much greater number of samples and, after further confirmation, this may prove to be one of the strongest epigenetic markers for poor survival in CCRCC.

The *secreted frizzled-related protein 1* (*SFRP1*) gene was hypermethylated in both cohorts, with one cohort demonstrating hypermethylation in >20% of tumors, and resides on the frequently lost chromosome 8p region. *SFRP1* hypermethylation strongly correlated with poorer patient survival in the HumanMethylation27 BeadChip cohort, with 3 of 6 statistically significant probes representing *SFRP1*. One probe represented a more significant prognostic marker for patient survival than tumor stage, grade or dimension and remaining two probes were statistically significant markers after tumor stage. In the HumanMethylation450 BeadChip cohort, hypermethylation of SFRP1 was the only remaining statistically significant marker of poorer survival after tumor stage. This data confirms a conclusion that has already been well demonstrated by numerous others that have shown both substantial levels of tumor specific *SFRP1* hypermethylation in RCC [16], [39]–[41] and that this methylation associates with higher grade and stage in tumors and predicts for poor prognosis in RCC patients [16], [40]. SFRP1 protein acts as a WNT antagonist, and thus a tumor suppressor, by inhibiting the activation of the WNT pathway, which when activated can influence cell proliferation, survival and invasion by the canonical/β-catenin pathway and cell adhesion, migration and cytoskeletal reorganization by the non-canonical pathway [42]. Dysregulation of the WNT pathway is a common event in a variety of cancers and this can result from the loss of WNT antagonists like *SFRP1*, its family members *SFRP2-5* and the *Wnt inhibitory factor 1* (*WIF1*) gene or by loss of the *Dickkoft* genes (*DKK1-4*) that bind the low-density lipoprotein receptor-related protein 5/6 component of the WNT receptor complex (Figure S7 in File S1) [42]. Previous publications have demonstrated hypermethylation of many of these genes in RCC [16], [39]–[41], [43]–[47] and within the TCGA data the *SFRP2*, *WIF1*, *DKK1* and *DKK2* genes all had probes that demonstrated hypermethylation in greater than 15% of samples in one cohort. Additionally, before correction for multiple analyses hypermethylation of *WIF1* associated with poorer survival in both cohorts and hypermethylation of *SFRP2* and *DKK2* associated with poorer survival in a single cohort (Figure S7 in File S1). In combination with the statistically significant association of hypermethylation of *SFRP1* with poorer patient survival, this suggests that the dysregulation of the WNT pathway is important for CCRCC tumor progression. Furthermore, although there is a very low rate of somatic mutation for *SFRP1* alone, 2.1% of the TCGA CCRCC tumors demonstrated mutually exclusive somatic mutation of regulators of the WNT pathway and this 2.1% had a statistically significantly (p = 0.017) worse rate of survival (Figure S8 in File S1). Thus, this data confirms the importance of hypermethylation of *SFRP1* and dysregulation of the WNT pathway in CCRCC and its relevance to poor patient survival. This may well have implications into therapy as a recent paper investigating the effects of both the demethylating drug decitabine (5-aza2′-deoxycytodine) and introduction of recombinant SRFP1 protein on renal and breast cancer lines demonstrated inhibition of cell growth and induction of apoptosis with both treatments [48].

In conclusion, the wealth and breadth of data provided by the Cancer Genome Altas programs are immense and provide an invaluable tool to researchers in many fields to both confirm and expand upon previous observations, as demonstrated herein, in a large data sat or to use as an investigative tool to produce new hypotheses for testing. By just using a fraction of the data, we hope to have provided convincing affirmation for four strong epigenetic markers of CCRCC, two of which may provide essential data for predicting poor patient survival and thus alter therapy. This may become particularly useful as the Ilumina BeadChip array analysis can be tailored to produce a specific set of probes, including newly designed ones, to assess specific genes and can be performed using very small amounts of material, such as that provided by a biopsy. The use of demethylating drugs has already proved successful with hematological malignances and hopefully advances in these therapies will improve their effectiveness in common solid tumors such as clear cell renal cell carcinoma.

## Supporting Information

File S1 **Includes Figures S1-S8 and Tables S1-S9. Figure S1:** Gene Map of the CpG Island for the *BNC1* gene demonstrating the Positioning of Probes and the Levels of Hypermethylation. **Figure S2:** The hypermethylated genes present in either both cohorts or just the HumanMethylation27 or 450 BeadChip cohort only. **Figure S3:** TCGA KIRC hypermethylated genes that occur in regions of common whole/partial chromosomal or focal loss/deletion. **Figure S4:** Somatic mutation maps for hypermethylated genes in the TCGA KIRC tumors. **Figure S5:** Kaplan-Meier survival curves for the most hypermethylated probes for each gene from the HumanMethylation450 BeadChip cohort. **Figure S6:** Relative tumor mRNA expression of selected candidate genes in methylated vs unmethylated tumors in the HumanMethylation450 BeadChip cohort. **Figure S7:** The WNT pathway and the potentially hypermethylated antagonists. **Figure S8:** Somatic mutation of the WNT pathway regulating genes and its affect on patient survival. **Table S1:** The 77 Candidate Hypermethylated Genes for Clear Cell Renal Cell Carcinoma Selected from Published Literature. **Table S2:** The Methylation Status of the 287 Infinium Probes Representing All 77 Candidate Hypermethylated Genes from the HumanMethylation27 BeadChip Cohort. **Table S3:** The 182 Hypermethylated Probes from the HumanMethylation450 BeadChip Cohort. **Table S4:** The Number of Hypermethylated Probes out of all the CpG Island Probes for Each Hypermethylated Gene from the HumanMethylation450 BeadChip Cohort. **Table S5:** Clinical Information for the HumanMethylation27 BeadChip Cohort and Hypermethylation Status for Significant Genes. **Table S6:** Clinical Information for the HumanMethylation450 BeadChip Cohort and Hypermethylation Status for Significant Genes. **Table S7:** Cox Proportional-Hazard Regression Analysis for the Probes that were Statistically Significantly Associated with Poorer Survival in Patients from the HumanMethylation27 BeadChip Cohort compared with Tumor Stage, Tumor Grade, Tumor Maximum Dimension and Patient Gender. **Table S8:** Kaplan-Meier Survival Curve Analysis for the Single Most Frequently Hypermethylated Probe for each Hypermethylated Gene in the HumanMethylation450 BeadChip Cohort and the Non-Significant Gene Level Results for the HumanMethylation450 BeadChip Cohort. **Table S9:** Cox Proportional-Hazard Regression Analysis for the Probes or Genes that were Statistically Significantly Associated with Poorer Survival in Patients from the HumanMethylation450 BeadChip Cohort compared with Tumor Stage, Tumor Grade, Tumor Maximum Dimension and Patient Gender.
(DOCX)
