# Supplementary material for: Tumor-Specific Hypermethylation of Epigenetic Biomarkers, Including SFRP1, Predicts for Poorer Survival in Patients from the TCGA Kidney Renal Clear Cell Carcinoma (KIRC) Project
Source: PLoS One. 2014 Jan 15;9(1):e85621. doi: 10.1371/journal.pone.0085621 (PMC3893219; doi:10.1371/journal.pone.0085621)
Supplement: File S1 — Includes Figures S1-S8 and Tables S1-S9. Figure S1: Gene Map of the CpG Island for the BNC1 gene demonstrating the Positioning of Probes and the Levels of Hypermethylation. Figure S2: The hypermethylated genes present in either both cohorts or just the HumanMethylation27 or 450 BeadChip cohort only. Figure S3: TCGA KIRC hypermethylated genes that occur in regions of common whole/partial chromosomal or focal loss/deletion. Figure S4: Somatic mutation maps for hypermethylated genes in the TCGA KIRC tumors. Figure S5: Kaplan-Meier survival curves for the most hypermethylated probes for each gene from the HumanMethylation450 BeadChip cohort. Figure S6: Relative tumor mRNA expression of selected candidate genes in methylated vs unmethylated tumors in the HumanMethylation450 BeadChip cohort. Figure S7: The WNT pathway and the potentially hypermethylated antagonists. Figure S8: Somatic mutation of the WNT pathway regulating genes and its affect on patient survival. Table S1: The 77 Candidate Hypermethylated Genes for Clear Cell Renal Cell Carcinoma Selected from Published Literature. Table S2: The Methylation Status of the 287 Infinium Probes Representing All 77 Candidate Hypermethylated Genes from the HumanMethylation27 BeadChip Cohort. Table S3: The 182 Hypermethylated Probes from the HumanMethylation450 BeadChip Cohort. Table S4: The Number of Hypermethylated Probes out of all the CpG Island Probes for Each Hypermethylated Gene from the HumanMethylation450 BeadChip Cohort. Table S5: Clinical Information for the HumanMethylation27 BeadChip Cohort and Hypermethylation Status for Significant Genes. Table S6: Clinical Information for the HumanMethylation450 BeadChip Cohort and Hypermethylation Status for Significant Genes. Table S7: Cox Proportional-Hazard Regression Analysis for the Probes that were Statistically Significantly Associated with Poorer Survival in Patients from the HumanMethylation27 BeadChip Cohort compared with Tumor Stage, Tumor Grade, Tumor Maximum Dimens [file pone.0085621.s001.docx]

**Supporting Materials For**

**Tumor-Specific Hypermethylation of Epigenetic Biomarkers, including *SFRP1,* Predicts for Poorer Survival in Patients from the TCGA Kidney Renal Clear Cell Carcinoma (KIRC) Project**

Christopher J. Ricketts ^1^, Victoria K. Hill ^2^, W. Marston Linehan ^1^

Affiliations:

^1^ Urologic Oncology Branch, Center for Cancer Research, National Cancer Institute, National Institutes of Health, Bethesda, MD, USA.

^2^ Cancer Genetics Branch, National Human Genome Research Institute, National Institutes of Health, Bethesda, MD, USA

Correspondence to:

W. Marston Linehan, M.D.

Urologic Oncology Branch,

National Cancer Institute

Building 10 CRC Room 1-5940

Bethesda, MD 20892-1107 USA

Tel: 301-496-6353

Fax: 301-402-0922

Email: [WML@nih.gov](mailto:WML@nih.gov)

**Supporting Figure Legends**

**Figure S1: Gene Map of the CpG Island for the *BNC1* gene demonstrating the Positioning of Probes and the Levels of Hypermethylation.**

This demonstrates the first exon of BNC1 gene with the predicted CpG island region (green box), the position and methylation levels of the HumanMethylation27 BeadChip array probes (red lines). The graphs for the HumanMethylation27 BeadChip array probes split the samples into two groups; those with tumor-specific hypermethylation (by the criteria described within) and those without. The average -values for the tumors and associated normals were graphed for each group and the standard deviation used to produce error bars.

**Figure S2: The hypermethylated genes present in either both cohorts or just the HumanMethylation27 or 450 BeadChip cohort only.**

This Venn diagram demonstrated the overlap in hypermethylated genes between the HumanMethylation27 BeadChip cohort and the HumanMethylation450 BeadChip cohort.

**Figure S3: TCGA KIRC hypermethylated genes that occur in regions of common whole/partial chromosomal or focal loss/deletion.**

The regions of chromosomal variation identified from the published TCGA Kidney Renal Clear Cell Carcinoma (KIRC) project data for all human chromosomes were mapped with the regions of whole/partial chromosomal loss/deletion or amplification highlighted with blue bars or red bars respectively and regions of focal loss or gain designated with blue or red arrows respectively. Genes that demonstrated tumor-specific hypermethylation in either cohort were designated with black arrows and those genes that were present in both cohorts were highlighted in bold.

**Figure S4: Somatic mutation maps for hypermethylated genes in the TCGA KIRC tumors.**

These schematics show the position of the somatic mutations in all of the previously published hypermethylated genes that were also hypermethylated in the TCGA that had 4 or more somatic mutations out of 424 exome sequenced tumors (0.9% or more), excluding *VHL.* Below each schematic is a table describing the mutation and its predicted effect, all data was obtained from the the cBioPortal for Cancer Genomics provided by the Memorial Sloan-Kettering Cancer Center (<http://www.cbioportal.org/public-portal/>).

**Figure S5: Kaplan-Meier survival curves for the most hypermethylated probes for each gene from the HumanMethylation450 BeadChip cohort.**

Kaplan-Meier survival curves were calculated for the most frequently hypermethylated probes for each gene within the HumanMethylation450 BeadChip cohort and these graphs represent the probes for which the p-value remained significant after false discovery rate (FDR) correction for multiple analyses. The percentage of hypermethylation (Meth) is representative of the 145 CCRCC tumor/normal pairs for which clinical information was available. The relevant BeadChip probe is listed with the gene name.

**Figure S6: Relative tumor mRNA expression of selected candidate genes in methylated vs unmethylated tumors in the HumanMethylation450 BeadChip cohort**

This graph represents the mRNA expression levels for the 4 selected candidate hypermethylated genes in the methylated tumors compared to the unmethylated tumors in the HumanMethylation 450 BeadChip cohort. For each gene, the expression level of the unmethylated tumors was averaged and considered 100% expression. The average expression level for each specific gene within the methylated tumors was then calculated relative to this 100% level. Of the 160 samples within the HumanMethylation450 BeadChip cohort, 134 tumors had RNASeq data available.

**Figure S7: The WNT pathway and the potentially hypermethylated antagonists.**

This schematic shows the basic control of the WNT pathway by its antagonists. The WNT receptor complex activated by binding the WNT ligand to the Frizzled receptor in conjunction with the presence of the low-density lipoprotein receptor-related protein 5/6 (LRP5/LRP6). This activation can be suppressed by the sequestration of WNT by the secreted frizzed gene family (SFRP1-5) or the Wnt inhibitory factor 1 (WIF-1) or by endocytosis of the LRP5/LRP6 complex component by the Dickkoft gene family (DKK1-4). With the WNT receptor complex in an inactive state the GSK3β/AXIN/APC complex is free to degrade and inactivate β-catenin, but once WNT is bound the WNT receptor complex is activated and can recruit the dishevelled homolog protein (DVL2). Once DVL2 is recruited this results in activation of the non-canonical WNT pathway and repression of the GSK3β/AXIN/APC complex, which results in the accumulation of β-catenin and activation of the canonical WNT pathway. Repression of the WNT pathway is often negated in cancer cells by epigenetic inactivation or chromosomal deletion of these WNT pathway antagonists. SFRP1 is highlighted in dark grey as it was frequently hypermethylated in both cohorts and associated with poorer survival. The gene highlighted in light grey were frequently hypermethylated in both cohorts and associated with poorer survival before correction for multiple analyses..

**Figure S8: Somatic mutation of the WNT pathway regulating genes and its affect on patient survival.**

The somatic mutation rate for the WNT pathway antagonist genes, SFRP1, SFRP2, FRZB (SFRP3), SFRP4, SFRP5, WIF1, DKK1, DKK2, DKK3 and DKK4, was 9 out of 424 tumors (2.1%) and demonstrated mutual exclusivity. A comparison of the survival rate for the patients with these 9 tumors compared to the remaining patients demonstrated a statistically significant trend for poorer survival with WNT pathway antagonist gene mutation. All data and survival analysis was obtained from the the cBioPortal for Cancer Genomics provided by the Memorial Sloan-Kettering Cancer Center (<http://www.cbioportal.org/public-portal/>).

**Figure S1: Gene Map of the CpG Island for the *BNC1* gene demonstrating the Positioning of Probes and the Levels of Hypermethylation.**

**
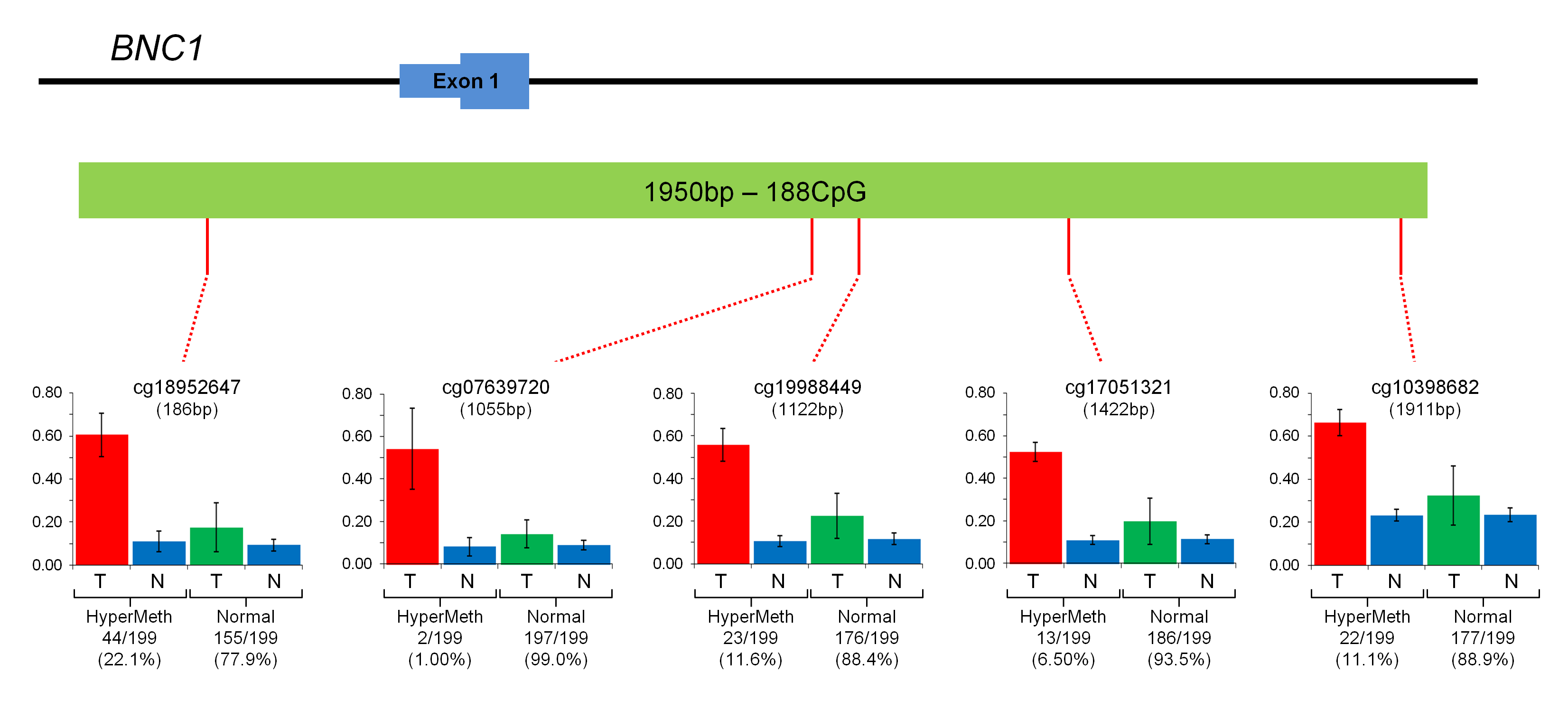
**

**Figure S2: The hypermethylated genes present in either both cohorts or just the HumanMethylation27 or 450 BeadChip cohort only.**

**
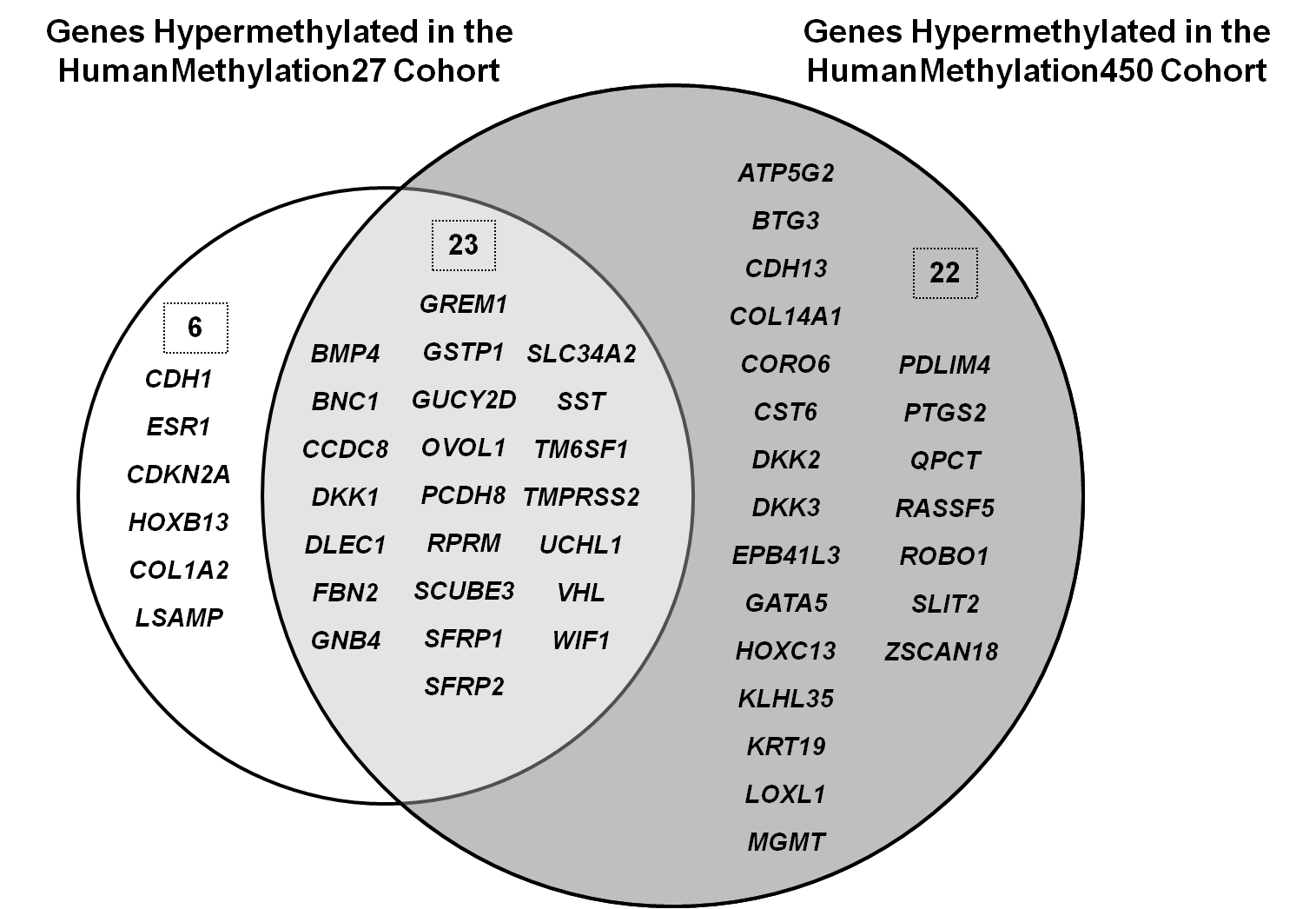
**

**Figure S2:** **TCGA KIRC hypermethylated genes that occur in regions of common whole/partial chromosomal or focal loss/deletion.**

**
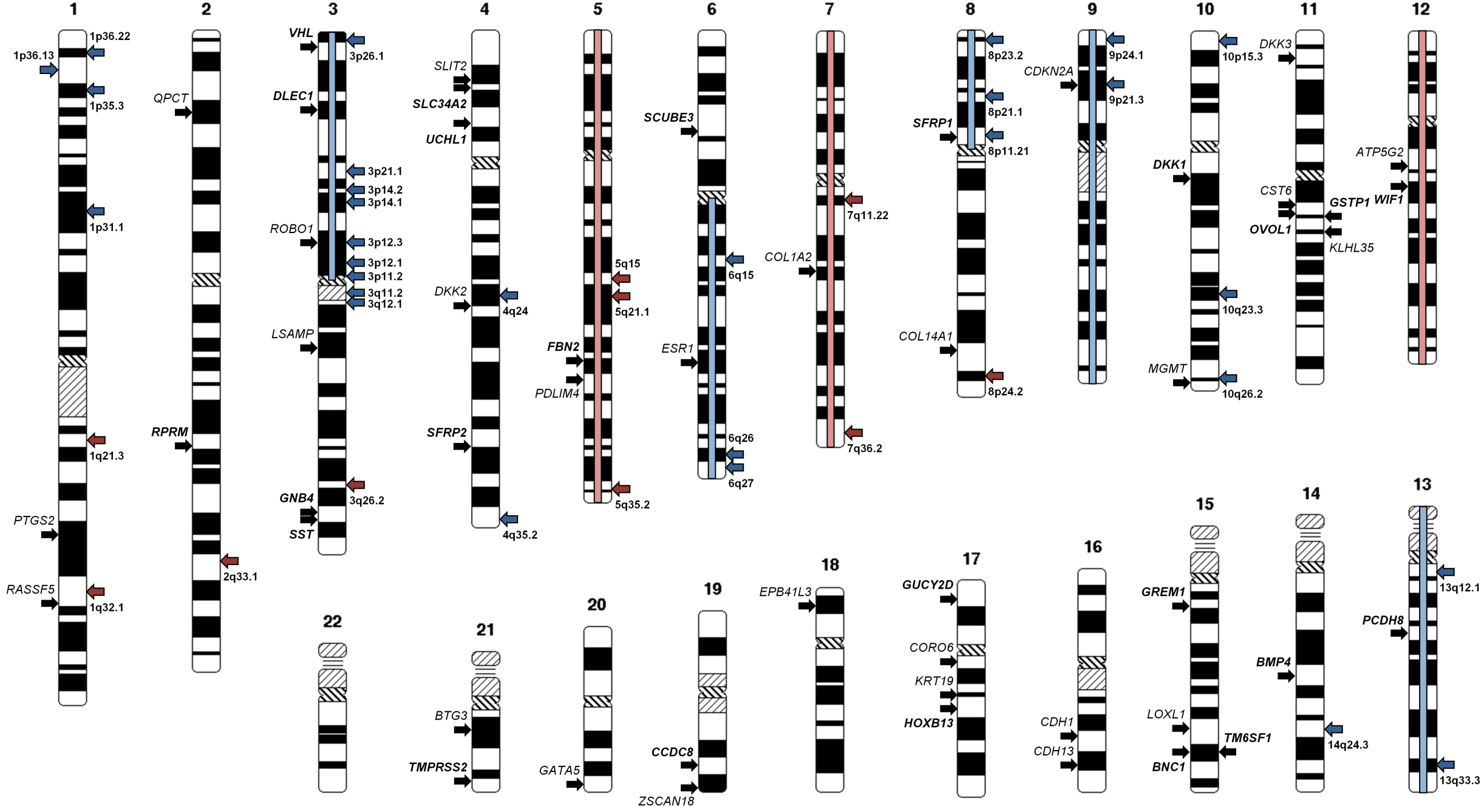
**

**Figure S4: Somatic mutation maps for hypermethylated genes in the TCGA KIRC tumors.**

**
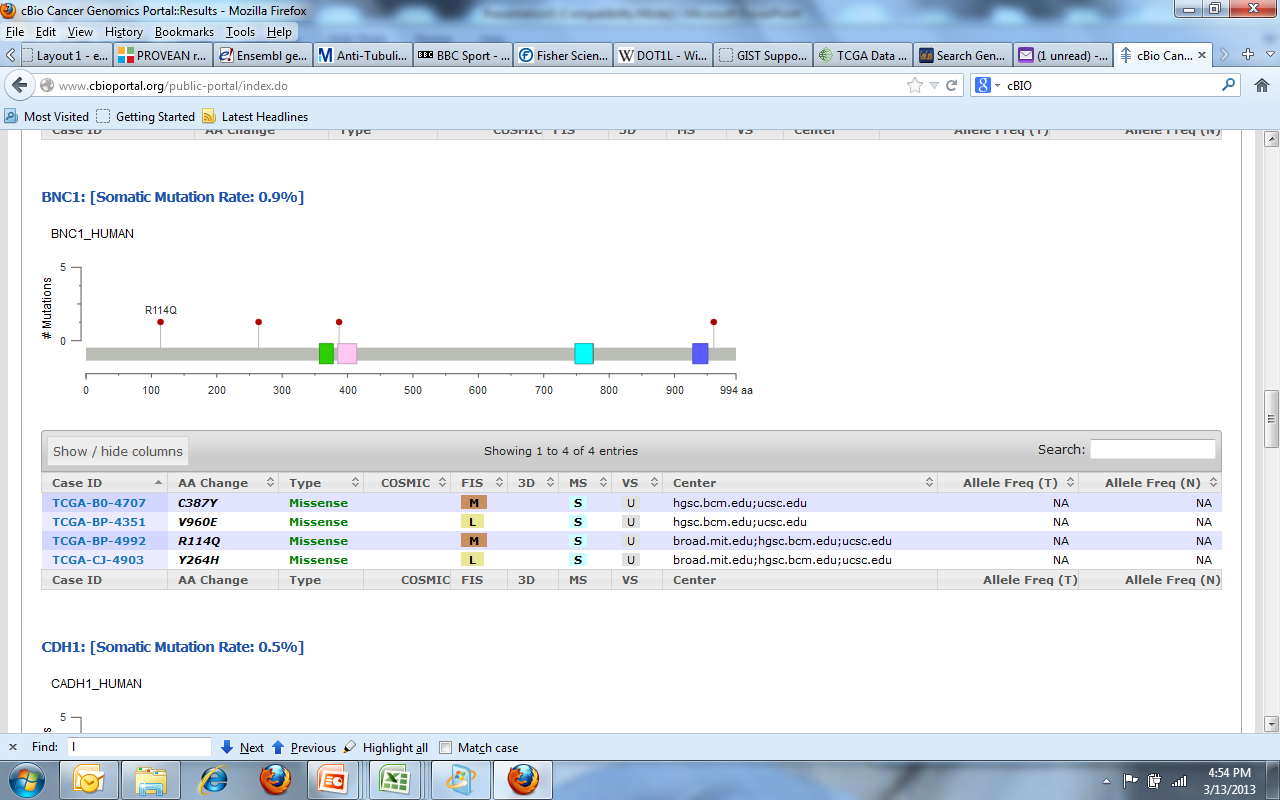
**

**
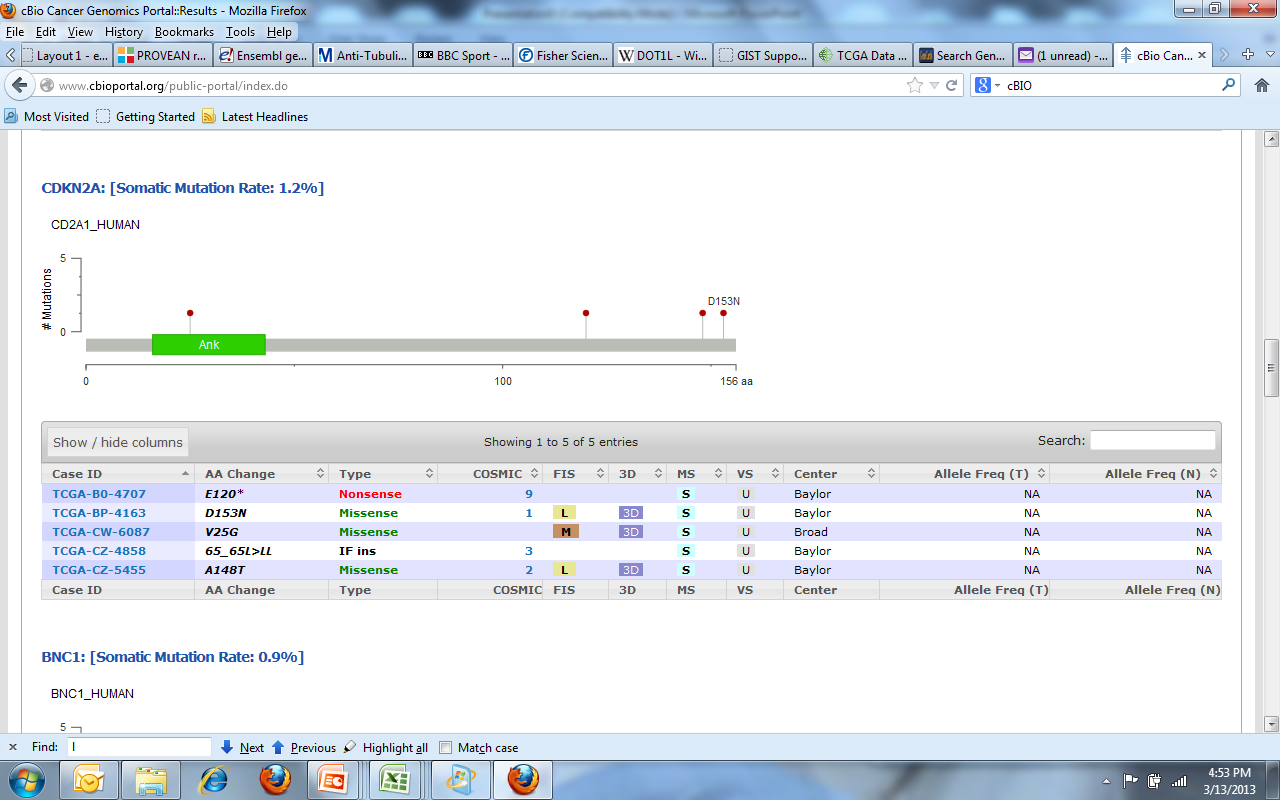
**

**
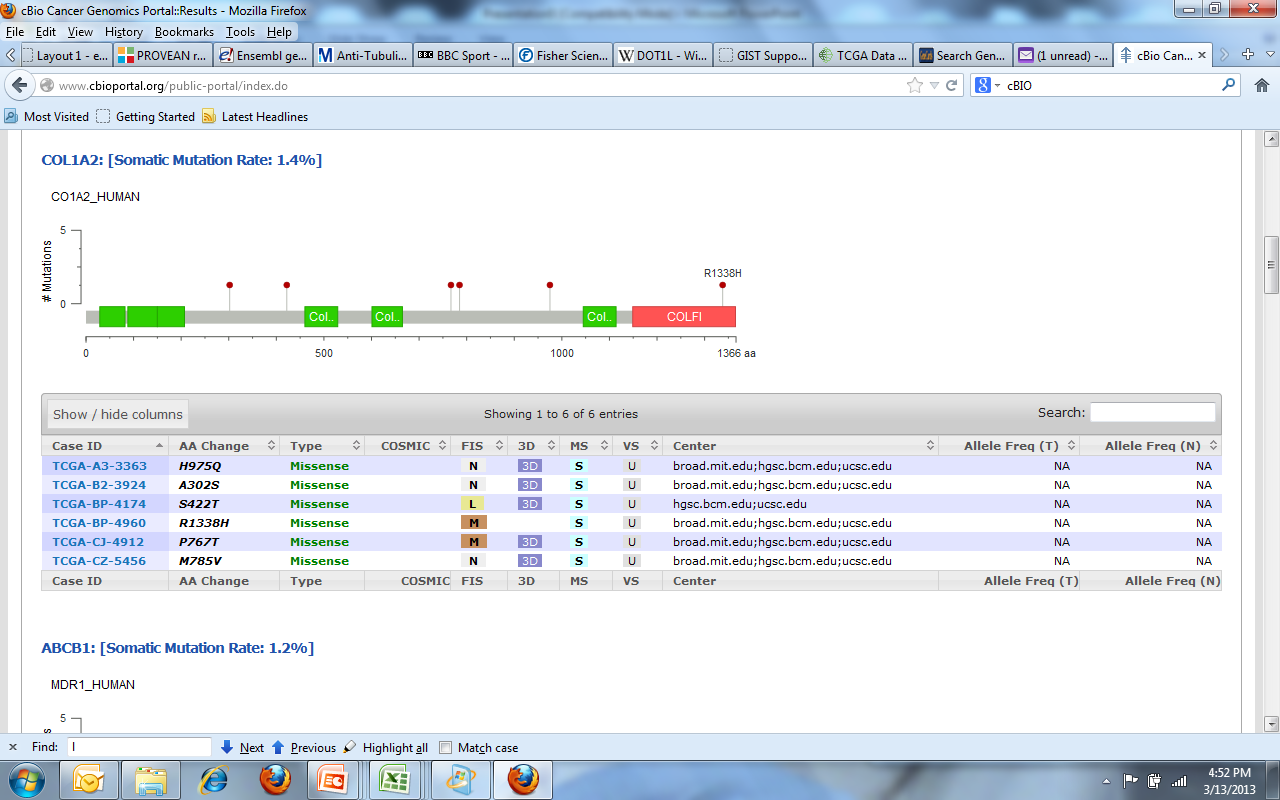
**

**Figure S4: Somatic mutation maps for hypermethylated genes in the TCGA KIRC tumors.**

**(Cont.)**

**
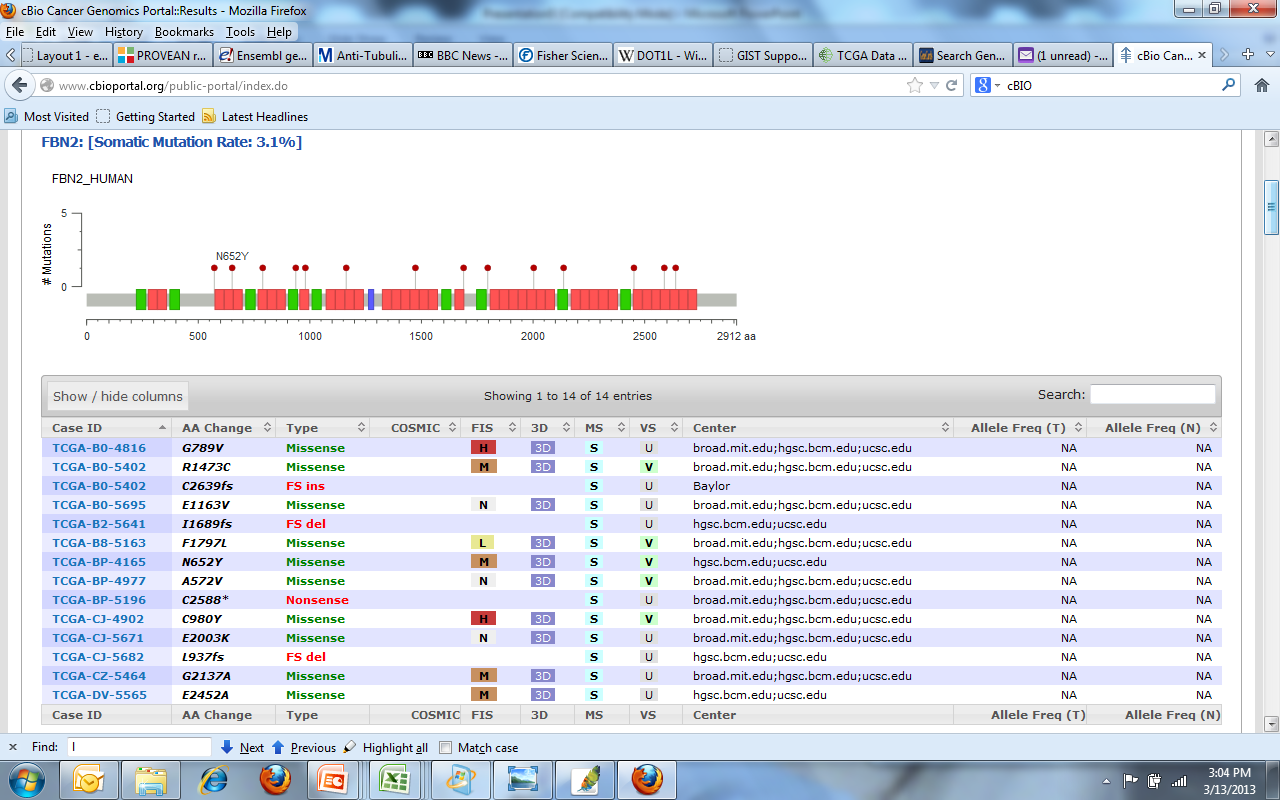
**

**
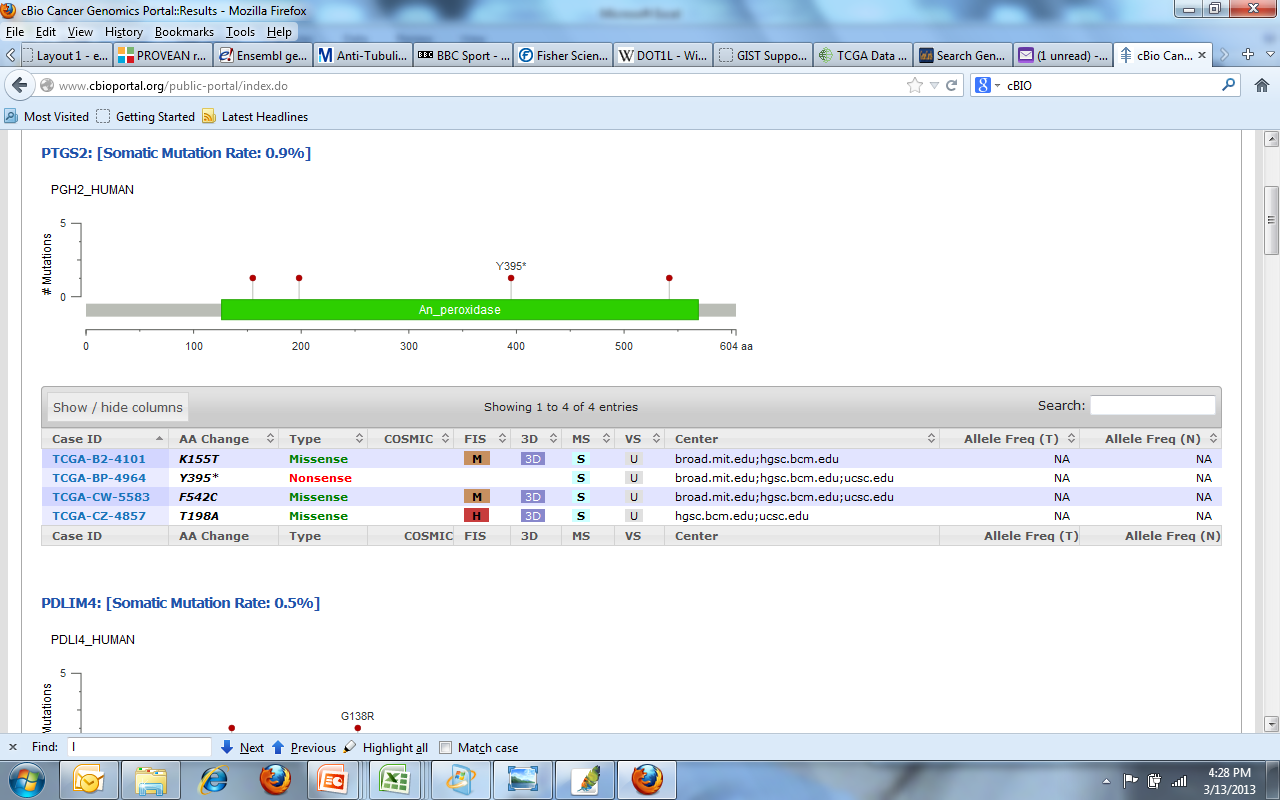
**

**
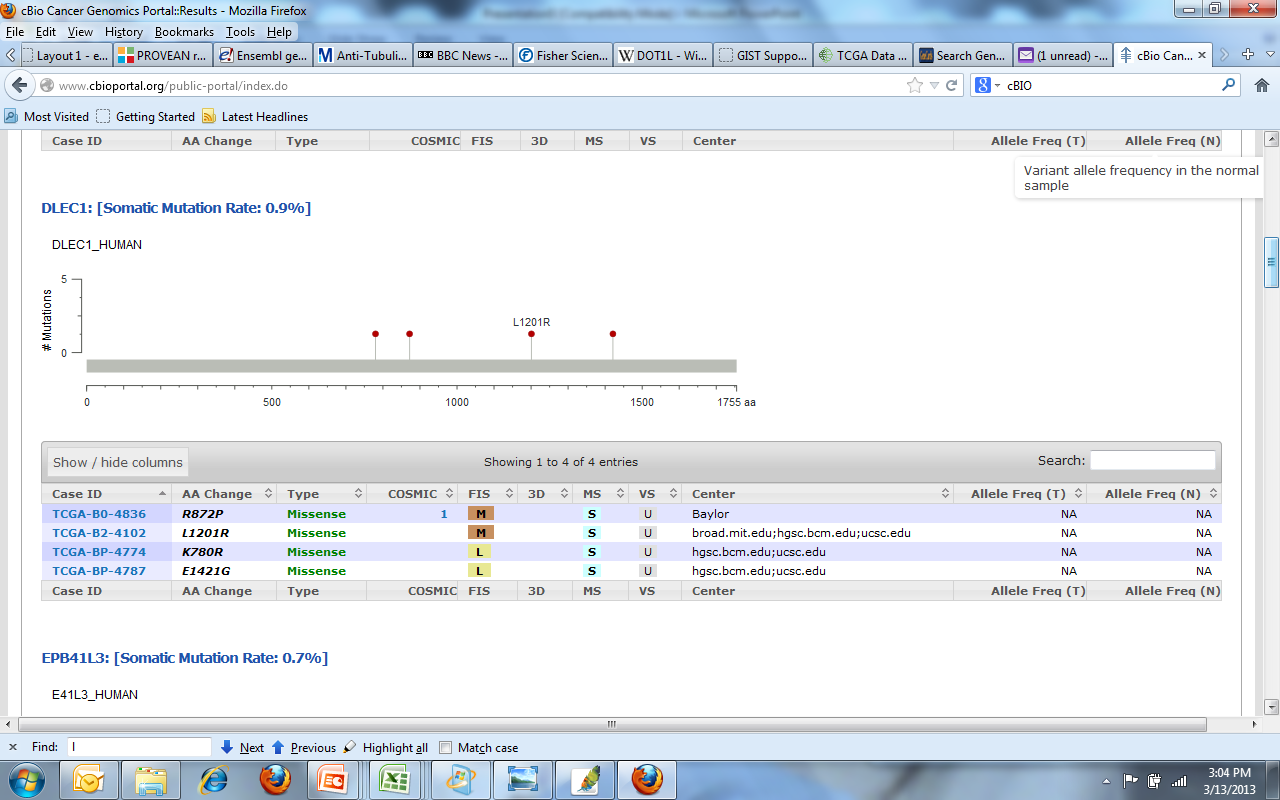
**

**Figure S5: Kaplan-Meier survival curves for the most hypermethylated probes for each gene from the HumanMethylation450 BeadChip cohort.**

**
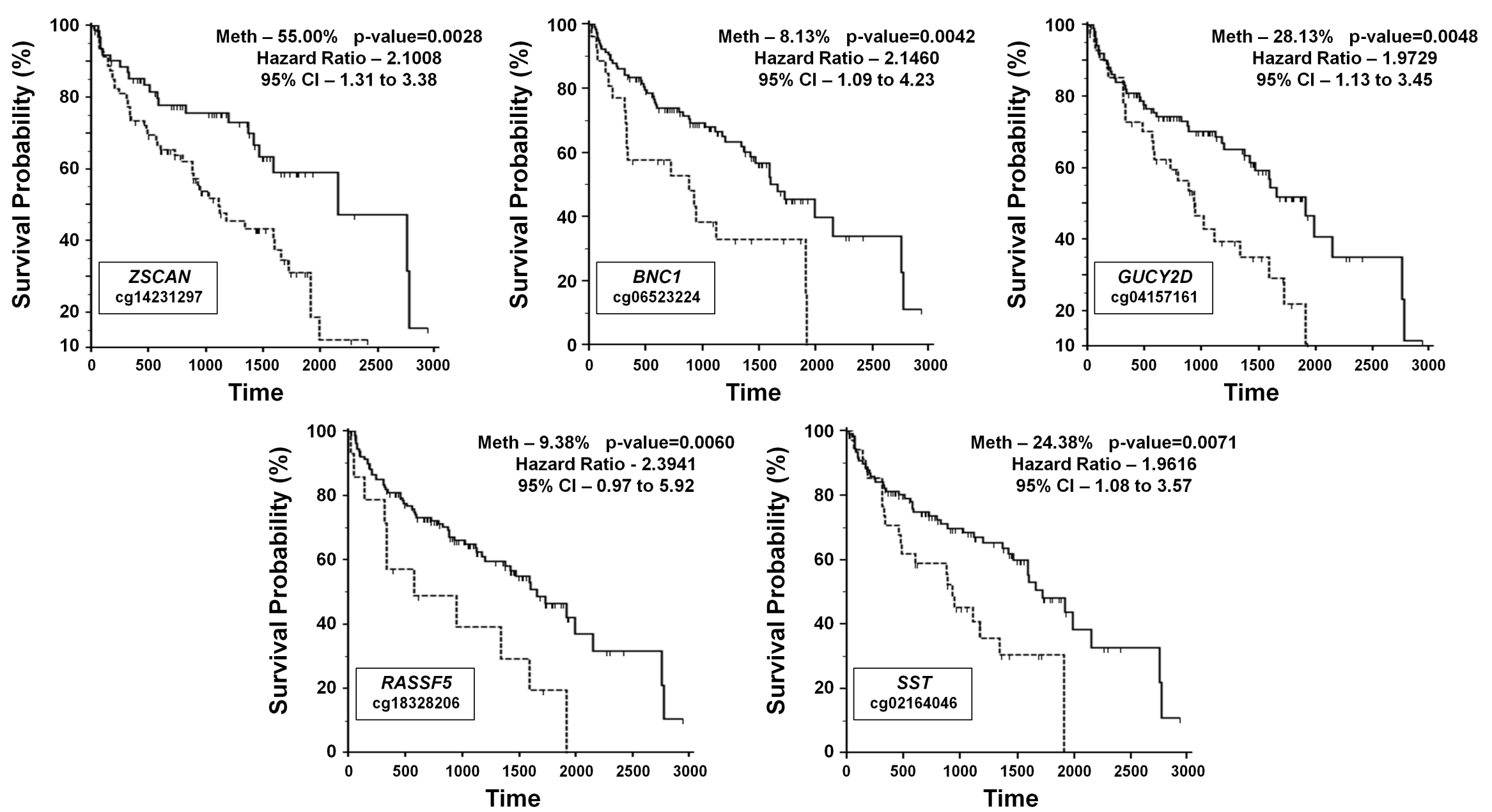
**

**Figure S6: Relative tumor mRNA expression of selected candidate genes in methylated vs unmethylated tumors in the HumanMethylation450 BeadChip cohort**

**
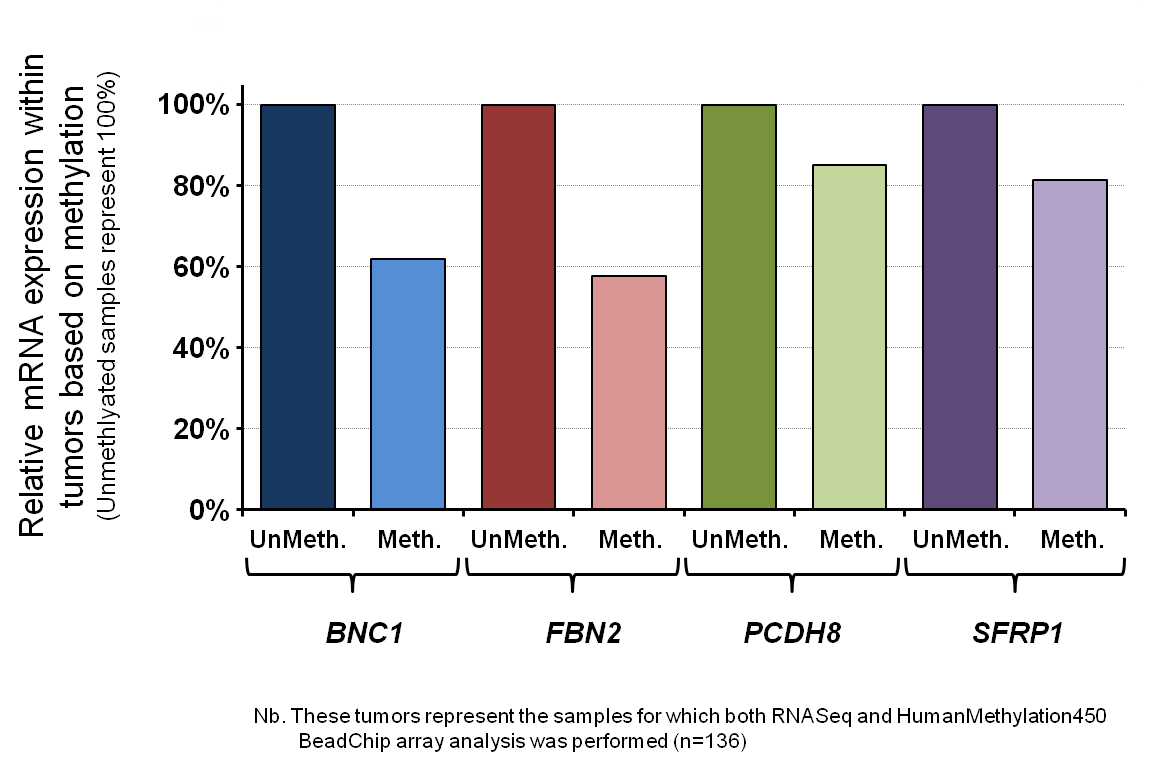
**

**Figure S7: The WNT pathway and the potentially hypermethylated antagonists.**

**
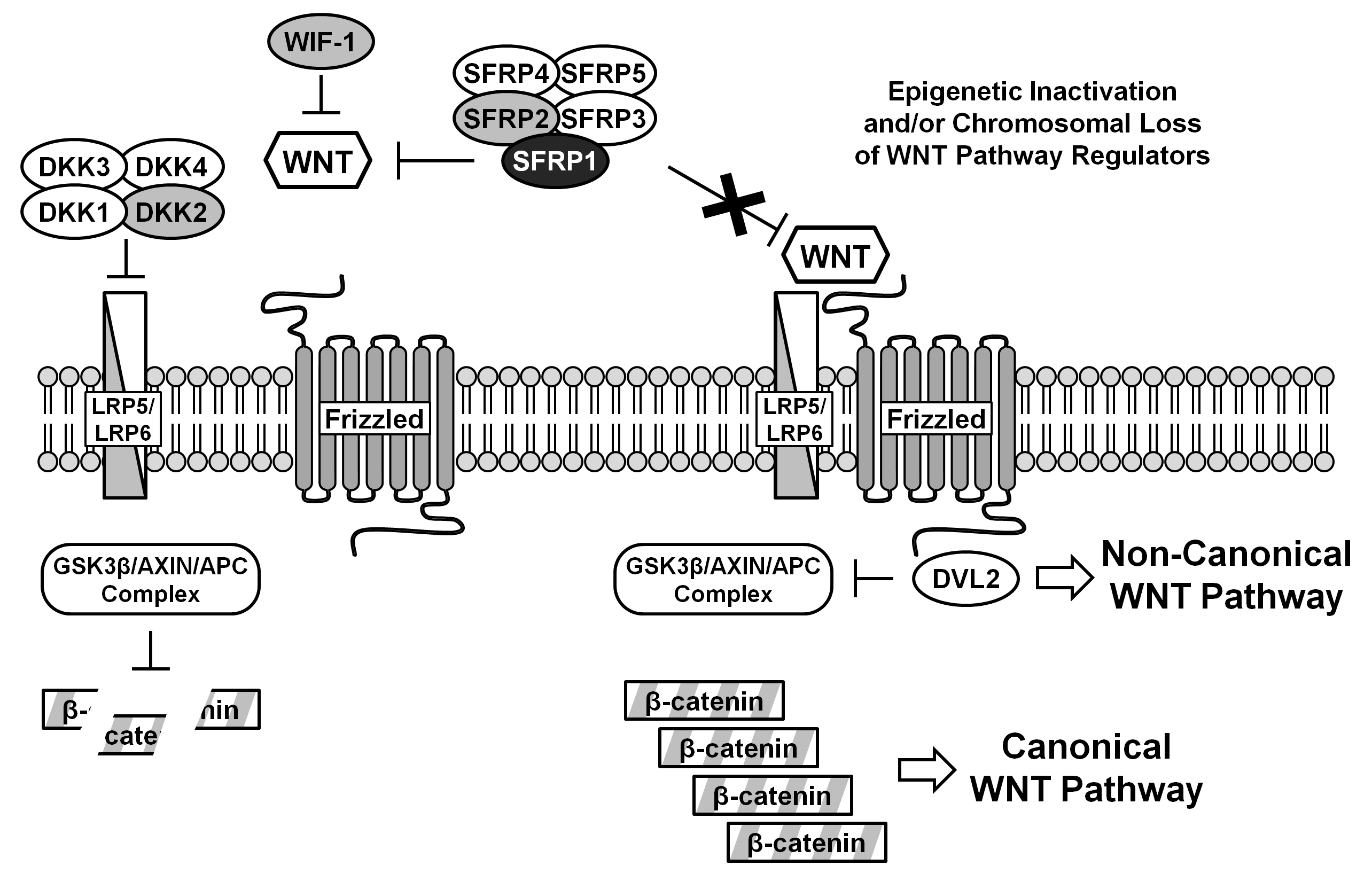
**

**Figure S8: Somatic mutation of the WNT pathway regulating genes and its affect on patient survival.**


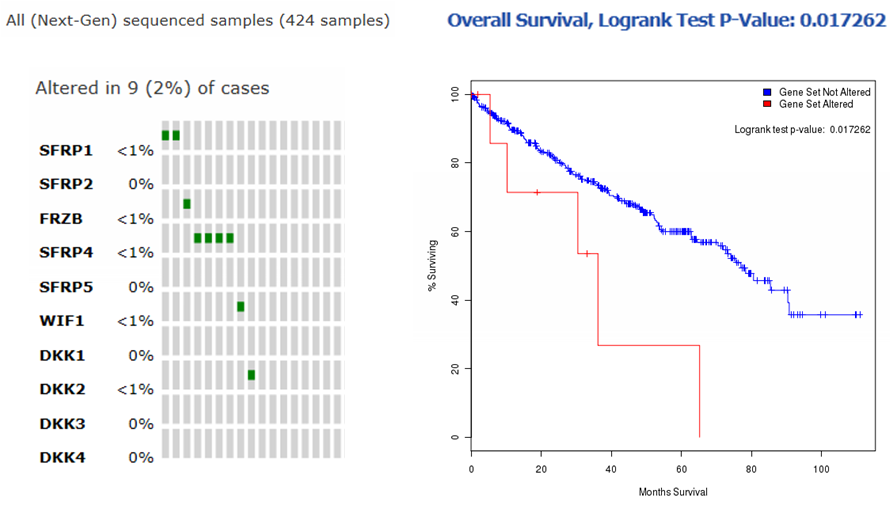


**Supporting Tables**

**Table S1: The 77 Candidate Hypermethylated Genes for Clear Cell Renal Cell Carcinoma Selected from Published Literature.**

| **Candidate Hypermethylated Genes in Clear Cell Renal Cell Carcinoma (CCRCC)** | | | | | | | |
| --- | --- | --- | --- | --- | --- | --- | --- |
|  |  |  |  |  |  |  |  |
| **1** | *ABCA1* | **21** | *DAPK1* | **41** | *HOXB13* | **61** | *SCUBE3* |
| **2** | *APAF1* | **22** | *DKK1* | **42** | *IGFBP1* | **62** | *SDHB* |
| **3** | *APC* | **23** | *DKK2* | **43** | *IGFBP3* | **63** | *SFRP1* |
| **4** | *ATP5G2* | **24** | *DKK3* | **44** | *JUP* | **64** | *SFRP2* |
| **5** | *BIRC4BP* | **25** | *DLC1* | **45** | *KRT19* | **65** | *SFRP4* |
| **6** | *BMP4* | **26** | *DLEC1* | **46** | *LOXL1* | **66** | *SFRP5* |
| **7** | *BNC1* | **27** | *EPB41L3* | **47** | *LSAMP* | **67** | *SLC34A2* |
| **8** | *BTG1* | **28** | *ESR1* | **48** | *MGMT* | **68** | *SLIT2* |
| **9** | *CASP8* | **29** | *ESR2* | **49** | *OVOL1* | **69** | *SPINT2* |
| **10** | *CCDC8* | **30** | *FAM107A* | **50** | *PCDH8* | **70** | *SST* |
| **11** | *CDH1* | **31** | *FBN2* | **51** | *PDLIM4* | **71** | *TIMP3* |
| **12** | *CDH13* | **32** | *FHIT* | **52** | *PML* | **72** | *TM6SF1* |
| **13** | *CDKN2A* | **33** | *FLCN* | **53** | *PTGS2* | **73** | *TMPRSS2* |
| **14** | *COL14A1* | **34** | *FLJ33790* | **54** | *QPCT* | **74** | *UCHL1* |
| **15** | *COL15A1* | **35** | *GATA5* | **55** | *RARB* | **75** | *VHL* |
| **16** | *COL1A1* | **36** | *GNB4* | **56** | *RASSF1* | **76** | *WIF1* |
| **17** | *COL1A2* | **37** | *GREM1* | **57** | *RASSF5* | **77** | *ZNF447* |
| **18** | *CORO6* | **38** | *GSTP1* | **58** | *RBP1* |  |  |
| **19** | *CST6* | **39** | *GUCY2D* | **59** | *ROBO1* |  |  |
| **20** | *CXCL16* | **40** | *HIC1* | **60** | *RPRM* |  |  |

**Table S2: The Methylation Status of the 287 Infinium Probes Representing All 77 Candidate Hypermethylated Genes from the HumanMethylation27 BeadChip Cohort.**

|  | **Gene Symbol** | **Infinium Probe ID** | **Percentage of 199 Tumors with Probe Difference Values ≥0.35** | **Average Associated Normal β-Value** |
| --- | --- | --- | --- | --- |
|  |  |  |  |  |
| **1** | ABCA1 | cg05286874 | 0.0% | 0.02 |
|  | ABCA1 | cg21572316 | 0.0% | 0.05 |
| **2** | APAF1 | cg00210842 | 0.0% | 0.11 |
|  | APAF1 | cg19196182 | 0.0% | 0.09 |
| **3** | APC | cg01240931 | 1.0% | 0.48 |
|  | APC | cg15020645 | 0.5% | 0.11 |
|  | APC | cg16970232 | 0.5% | 0.08 |
|  | APC | cg20311501 | 0.5% | 0.10 |
|  | APC | cg21634602 | 0.5% | 0.03 |
|  | APC | cg24332422 | 0.5% | 0.13 |
| **4** | ATP5G2 | cg08995424 | 3.5% | 0.08 |
|  | ATP5G2 | cg22546318 | 0.5% | 0.08 |
| **5** | BIRC4BP | cg23571857 | 0.0% | 0.69 |
|  | BIRC4BP | cg27146152 | 0.0% | 0.15 |
| **6** | BMP4 | cg14310034 | 8.0% | 0.08 |
|  | BMP4 | cg24526899 | 0.0% | 0.48 |
| **7** | BNC1 | cg18952647 | 22.1% | 0.10 |
|  | BNC1 | cg15736165 | 11.6% | 0.21 |
|  | BNC1 | cg19988449 | 11.6% | 0.12 |
|  | BNC1 | cg10398682 | 11.1% | 0.23 |
|  | BNC1 | cg17051321 | 6.5% | 0.11 |
|  | BNC1 | cg07639720 | 1.0% | 0.09 |
|  | BNC1 | cg27169020 | 1.0% | 0.53 |
| **8** | BTG1 | cg04211745 | 0.0% | 0.06 |
|  | BTG1 | cg13333141 | 0.0% | 0.02 |
| **9** | CASP8 | cg05130485 | 0.0% | 0.32 |
|  | CASP8 | cg22898761 | 0.0% | 0.76 |
|  | CASP8 | cg23410113 | 0.0% | 0.80 |
|  | CASP8 | cg25095814 | 0.0% | 0.24 |
|  | CASP8 | cg26799474 | 0.0% | 0.75 |
|  | CASP8 | cg26986815 | 0.0% | 0.81 |
| **10** | CCDC8 | cg15984661 | 5.5% | 0.15 |
|  | CCDC8 | cg11715966 | 0.0% | 0.72 |
| **11** | CDH1 | cg24765079 | 5.5% | 0.20 |
|  | CDH1 | cg00240312 | 1.0% | 0.04 |
|  | CDH1 | cg11667754 | 1.0% | 0.09 |
|  | CDH1 | cg20716119 | 0.5% | 0.11 |
|  | CDH1 | cg11255163 | 0.0% | 0.04 |
|  | CDH1 | cg17655614 | 0.0% | 0.41 |
|  | CDH1 | cg22832044 | 0.0% | 0.15 |
|  | CDH1 | cg23989635 | 0.0% | 0.12 |
|  | **Gene Symbol** | **Infinium Probe ID** | **Percentage of 199 Tumors with Probe Difference Values ≥0.35** | **Average Associated Normal β-Value** |
|  |  |  |  |  |
| **12** | CDH13 | cg19369556 | 4.5% | 0.17 |
|  | CDH13 | cg13759328 | 4.0% | 0.07 |
|  | CDH13 | cg08977371 | 3.0% | 0.10 |
|  | CDH13 | cg00806490 | 2.0% | 0.19 |
|  | CDH13 | cg08747377 | 1.0% | 0.05 |
|  | CDH13 | cg01880569 | 0.0% | 0.42 |
|  | CDH13 | cg02168291 | 0.0% | 0.72 |
|  | CDH13 | cg16777782 | 0.0% | 0.94 |
|  | CDH13 | cg19854301 | 0.0% | 0.90 |
| **13** | CDKN2A | cg09099744 | 11.1% | 0.09 |
|  | CDKN2A | cg10895543 | 10.1% | 0.15 |
|  | CDKN2A | cg07752420 | 9.0% | 0.13 |
|  | CDKN2A | cg11653709 | 4.0% | 0.17 |
|  | CDKN2A | cg12840719 | 1.5% | 0.22 |
|  | CDKN2A | cg00718440 | 0.0% | 0.09 |
|  | CDKN2A | cg03079681 | 0.0% | 0.06 |
|  | CDKN2A | cg13479669 | 0.0% | 0.14 |
|  | CDKN2A | cg26673943 | 0.0% | 0.08 |
| **14** | COL14A1 | cg23196831 | 4.0% | 0.09 |
|  | COL14A1 | cg16907566 | 1.5% | 0.36 |
| **15** | COL15A1 | cg19461644 | 4.5% | 0.08 |
|  | COL15A1 | cg20503329 | 0.0% | 0.66 |
| **16** | COL1A1 | cg01234133 | 1.0% | 0.14 |
|  | COL1A1 | cg01593886 | 0.5% | 0.11 |
| **17** | COL1A2 | cg18511007 | 16.1% | 0.17 |
|  | COL1A2 | cg25300386 | 12.1% | 0.19 |
| **18** | CORO6 | cg06038133 | 4.0% | 0.28 |
|  | CORO6 | cg26388152 | 0.5% | 0.09 |
| **19** | CST6 | cg22524514 | 4.0% | 0.20 |
|  | CST6 | cg15887846 | 1.5% | 0.40 |
| **20** | CXCL16 | cg18777448 | 0.0% | 0.94 |
|  | CXCL16 | cg19478743 | 0.0% | 0.04 |
| **21** | DAPK1 | cg24754277 | 3.5% | 0.25 |
|  | DAPK1 | cg15746719 | 1.0% | 0.05 |
|  | DAPK1 | cg08719486 | 0.0% | 0.42 |
|  | DAPK1 | cg08797471 | 0.0% | 0.15 |
|  | DAPK1 | cg13814950 | 0.0% | 0.05 |
|  | DAPK1 | cg17090012 | 0.0% | 0.82 |
|  | DAPK1 | cg19734228 | 0.0% | 0.04 |
|  | DAPK1 | cg22571217 | 0.0% | 0.05 |

|  | **Gene Symbol** | **Infinium Probe ID** | **Percentage of 199 Tumors with Probe Difference Values ≥0.35** | **Average Associated Normal β-Value** |
| --- | --- | --- | --- | --- |
|  |  |  |  |  |
| **22** | DKK1 | cg07684796 | 8.5% | 0.11 |
|  | DKK1 | cg12621514 | 7.0% | 0.17 |
| **23** | DKK2 | cg01404615 | 2.0% | 0.07 |
|  | DKK2 | cg01471384 | 1.0% | 0.04 |
| **24** | DKK3 | cg13216057 | 2.5% | 0.04 |
|  | DKK3 | cg25787984 | 0.0% | 0.55 |
| **25** | DLC1 | cg00933411 | 0.0% | 0.61 |
|  | DLC1 | cg05226008 | 0.0% | 0.50 |
| **26** | DLEC1 | cg23881725 | 12.1% | 0.20 |
|  | DLEC1 | cg00807586 | 3.5% | 0.10 |
| **27** | EPB41L3 | cg00027083 | 2.5% | 0.36 |
| **28** | ESR1 | cg15626350 | 23.1% | 0.27 |
|  | ESR1 | cg02720618 | 5.0% | 0.14 |
|  | ESR1 | cg20253551 | 5.0% | 0.13 |
|  | ESR1 | cg07671949 | 3.0% | 0.33 |
|  | ESR1 | cg11251858 | 1.0% | 0.07 |
|  | ESR1 | cg00655307 | 0.0% | 0.08 |
|  | ESR1 | cg20627916 | 0.0% | 0.39 |
| **29** | ESR2 | cg11059483 | 1.0% | 0.06 |
|  | ESR2 | cg16792632 | 0.0% | 0.04 |
| **30** | FAM107A | cg06638451 | 3.0% | 0.31 |
|  | FAM107A | cg01446393 | 1.5% | 0.43 |
| **31** | FBN2 | cg27223047 | 40.2% | 0.13 |
|  | FBN2 | cg25084878 | 24.1% | 0.24 |
| **32** | FHIT | cg22215728 | 1.0% | 0.12 |
|  | FHIT | cg02854288 | 0.0% | 0.07 |
|  | FHIT | cg04835638 | 0.0% | 0.87 |
|  | FHIT | cg11158760 | 0.0% | 0.09 |
|  | FHIT | cg15931943 | 0.0% | 0.03 |
|  | FHIT | cg16986494 | 0.0% | 0.02 |
|  | FHIT | cg19049316 | 0.0% | 0.09 |
|  | FHIT | cg19574488 | 0.0% | 0.16 |
|  | FHIT | cg24796403 | 0.0% | 0.04 |
| **33** | FLCN | cg02975142 | 0.0% | 0.07 |
|  | FLCN | cg25448636 | 0.0% | 0.05 |
| **34** | FLJ33790 | cg12001148 | 4.0% | 0.08 |
|  | FLJ33790 | cg16547529 | 0.0% | 0.52 |

|  | **Gene Symbol** | **Infinium Probe ID** | **Percentage of 199 Tumors with Probe Difference Values ≥0.35** | **Average Associated Normal β-Value** |
| --- | --- | --- | --- | --- |
|  |  |  |  |  |
| **35** | GATA5 | cg15749748 | 4.5% | 0.08 |
|  | GATA5 | cg14519000 | 0.5% | 0.90 |
|  | GATA5 | cg03777459 | 0.0% | 0.11 |
|  | GATA5 | cg04132607 | 0.0% | 0.80 |
|  | GATA5 | cg09864990 | 0.0% | 0.73 |
|  | GATA5 | cg09923671 | 0.0% | 0.58 |
|  | GATA5 | cg16542081 | 0.0% | 0.83 |
|  | GATA5 | cg20822628 | 0.0% | 0.88 |
| **36** | GNB4 | cg17483510 | 9.0% | 0.19 |
|  | GNB4 | cg09997760 | 5.5% | 0.04 |
| **37** | GREM1 | cg21296230 | 16.1% | 0.09 |
|  | GREM1 | cg18145505 | 1.5% | 0.15 |
| **38** | GSTP1 | cg04920951 | 13.6% | 0.10 |
|  | GSTP1 | cg02659086 | 5.0% | 0.06 |
|  | GSTP1 | cg09038676 | 5.0% | 0.18 |
|  | GSTP1 | cg11566244 | 3.5% | 0.38 |
|  | GSTP1 | cg22224704 | 3.5% | 0.35 |
|  | GSTP1 | cg25025243 | 2.0% | 0.46 |
|  | GSTP1 | cg05244766 | 0.5% | 0.77 |
| **39** | GUCY2D | cg25465406 | 8.0% | 0.10 |
|  | GUCY2D | cg14425294 | 0.5% | 0.32 |
| **40** | HIC1 | cg01168201 | 0.5% | 0.03 |
|  | HIC1 | cg17210604 | 0.0% | 0.10 |
|  | HIC1 | cg19001794 | 0.0% | 0.05 |
| **41** | HOXB13 | cg15786837 | 6.5% | 0.09 |
|  | HOXB13 | cg21842478 | 3.0% | 0.04 |
| **42** | IGFBP1 | cg05660795 | 0.0% | 0.17 |
|  | IGFBP1 | cg27447599 | 0.0% | 0.24 |
| **43** | IGFBP3 | cg22083798 | 0.5% | 0.14 |
|  | IGFBP3 | cg04796162 | 0.0% | 0.08 |
|  | IGFBP3 | cg06713098 | 0.0% | 0.05 |
|  | IGFBP3 | cg08831744 | 0.0% | 0.10 |
|  | IGFBP3 | cg15898840 | 0.0% | 0.06 |
| **44** | JUP | cg13784792 | 0.0% | 0.05 |
|  | JUP | cg17388484 | 0.0% | 0.09 |
| **45** | KRT19 | cg11462865 | 3.0% | 0.05 |
|  | KRT19 | cg16585619 | 0.5% | 0.73 |
| **46** | LOXL1 | cg14435807 | 2.5% | 0.25 |
|  | LOXL1 | cg03682712 | 1.5% | 0.14 |
| **47** | LSAMP | cg14294758 | 8.0% | 0.10 |
|  | LSAMP | cg25315362 | 2.0% | 0.35 |
|  |  |  |  |  |
|  |  |  |  |  |
|  | **Gene Symbol** | **Infinium Probe ID** | **Percentage of 199 Tumors with Probe Difference Values ≥0.35** | **Average Associated Normal β-Value** |
|  |  |  |  |  |
| **48** | MGMT | cg25946389 | 1.5% | 0.11 |
|  | MGMT | cg02941816 | 0.5% | 0.08 |
|  | MGMT | cg03271907 | 0.5% | 0.80 |
|  | MGMT | cg12434587 | 0.5% | 0.03 |
|  | MGMT | cg12981137 | 0.5% | 0.03 |
|  | MGMT | cg16698623 | 0.5% | 0.49 |
|  | MGMT | cg17686260 | 0.5% | 0.61 |
|  | MGMT | cg27056559 | 0.5% | 0.88 |
|  | MGMT | cg00904483 | 0.0% | 0.90 |
|  | MGMT | cg02330106 | 0.0% | 0.82 |
|  | MGMT | cg02381948 | 0.0% | 0.86 |
|  | MGMT | cg02803836 | 0.0% | 0.87 |
|  | MGMT | cg04473030 | 0.0% | 0.86 |
|  | MGMT | cg07453748 | 0.0% | 0.89 |
|  | MGMT | cg07638938 | 0.0% | 0.85 |
|  | MGMT | cg10036895 | 0.0% | 0.79 |
|  | MGMT | cg10333959 | 0.0% | 0.88 |
|  | MGMT | cg12040555 | 0.0% | 0.89 |
|  | MGMT | cg14129786 | 0.0% | 0.68 |
|  | MGMT | cg18488970 | 0.0% | 0.65 |
|  | MGMT | cg19680672 | 0.0% | 0.68 |
|  | MGMT | cg19706602 | 0.0% | 0.10 |
|  | MGMT | cg20537325 | 0.0% | 0.89 |
|  | MGMT | cg20778669 | 0.0% | 0.87 |
|  | MGMT | cg24580056 | 0.0% | 0.91 |
|  | MGMT | cg26201213 | 0.0% | 0.59 |
| **49** | OVOL1 | cg20909686 | 14.1% | 0.18 |
|  | OVOL1 | cg13496736 | 11.1% | 0.06 |
|  | OVOL1 | cg03681481 | 0.0% | 0.05 |
|  | OVOL1 | cg20315951 | 0.0% | 0.07 |
| **50** | PCDH8 | cg20366906 | 19.1% | 0.14 |
|  | PCDH8 | cg10484958 | 0.5% | 0.13 |
| **51** | PDLIM4 | cg01305625 | 0.0% | 0.60 |
|  | PDLIM4 | cg20512303 | 0.0% | 0.66 |
| **52** | PML | cg05697231 | 0.0% | 0.12 |
| **53** | PTGS2 | cg13986130 | 3.0% | 0.09 |
|  | PTGS2 | cg15774283 | 2.0% | 0.04 |
|  | PTGS2 | cg19155599 | 2.0% | 0.18 |
|  | PTGS2 | cg03160309 | 1.0% | 0.24 |
|  | PTGS2 | cg18335243 | 1.0% | 0.08 |
|  | PTGS2 | cg09461185 | 0.5% | 0.06 |

|  | **Gene Symbol** | **Infinium Probe ID** | **Percentage of 199 Tumors with Probe Difference Values ≥0.35** | **Average Associated Normal β-Value** |
| --- | --- | --- | --- | --- |
|  |  |  |  |  |
| **54** | QPCT | cg16029957 | 0.0% | 0.11 |
| **55** | RARB | cg27486427 | 0.5% | 0.06 |
|  | RARB | cg02499249 | 0.0% | 0.12 |
|  | RARB | cg10712623 | 0.0% | 0.21 |
|  | RARB | cg26124016 | 0.0% | 0.10 |
| **56** | RASSF1 | cg21554552 | 0.5% | 0.38 |
|  | RASSF1 | cg00777121 | 0.0% | 0.53 |
|  | RASSF1 | cg06063729 | 0.0% | 0.09 |
|  | RASSF1 | cg06821120 | 0.0% | 0.44 |
|  | RASSF1 | cg06980053 | 0.0% | 0.49 |
|  | RASSF1 | cg08047457 | 0.0% | 0.55 |
|  | RASSF1 | cg11035216 | 0.0% | 0.02 |
|  | RASSF1 | cg15043975 | 0.0% | 0.15 |
|  | RASSF1 | cg26357744 | 0.0% | 0.08 |
| **57** | RASSF5 | cg24450312 | 1.0% | 0.12 |
|  | RASSF5 | cg23520347 | 0.5% | 0.09 |
|  | RASSF5 | cg01860753 | 0.0% | 0.17 |
|  | RASSF5 | cg02589695 | 0.0% | 0.55 |
|  | RASSF5 | cg08617916 | 0.0% | 0.07 |
|  | RASSF5 | cg10167296 | 0.0% | 0.06 |
|  | RASSF5 | cg17558126 | 0.0% | 0.16 |
|  | RASSF5 | cg19452316 | 0.0% | 0.04 |
|  | RASSF5 | cg22857604 | 0.0% | 0.04 |
| **58** | RBP1 | cg12497564 | 1.5% | 0.03 |
|  | RBP1 | cg23363832 | 1.5% | 0.28 |
|  | RBP1 | cg06543018 | 1.0% | 0.27 |
|  | RBP1 | cg11027570 | 0.5% | 0.12 |
|  | RBP1 | cg13099330 | 0.5% | 0.54 |
|  | RBP1 | cg24594997 | 0.0% | 0.04 |
|  | RBP1 | cg27457941 | 0.0% | 0.08 |
| **59** | ROBO1 | cg20145360 | 0.0% | 0.73 |
| **60** | RPRM | cg27420236 | 9.0% | 0.08 |
|  | RPRM | cg18411898 | 1.0% | 0.20 |
| **61** | SCUBE3 | cg21604042 | 13.1% | 0.07 |
|  | SCUBE3 | cg00347904 | 4.5% | 0.10 |
| **62** | SDHB | cg03861428 | 0.0% | 0.02 |
|  | SDHB | cg24305835 | 0.0% | 0.04 |
| **63** | SFRP1 | cg22418909 | 16.1% | 0.08 |
|  | SFRP1 | cg13398291 | 11.1% | 0.09 |
|  | SFRP1 | cg15839448 | 7.0% | 0.05 |
|  | SFRP1 | cg02388150 | 4.5% | 0.27 |
|  | SFRP1 | cg06166767 | 0.0% | 0.32 |

|  | **Gene Symbol** | **Infinium Probe ID** | **Percentage of 199 Tumors with Probe Difference Values ≥0.35** | **Average Associated Normal β-Value** |
| --- | --- | --- | --- | --- |
|  |  |  |  |  |
| **64** | SFRP2 | cg23207990 | 9.0% | 0.07 |
|  | SFRP2 | cg07694025 | 2.5% | 0.27 |
|  | SFRP2 | cg11354906 | 1.0% | 0.10 |
|  | SFRP2 | cg05774801 | 0.5% | 0.11 |
|  | SFRP2 | cg23910835 | 0.0% | 0.14 |
| **65** | SFRP4 | cg20019546 | 1.5% | 0.19 |
|  | SFRP4 | cg08261094 | 0.0% | 0.15 |
|  | SFRP4 | cg10806140 | 0.0% | 0.05 |
|  | SFRP4 | cg12515638 | 0.0% | 0.05 |
|  | SFRP4 | cg16433922 | 0.0% | 0.10 |
|  | SFRP4 | cg19166347 | 0.0% | 0.13 |
| **66** | SFRP5 | cg09874752 | 2.5% | 0.04 |
|  | SFRP5 | cg25156443 | 2.5% | 0.19 |
|  | SFRP5 | cg09542745 | 2.0% | 0.10 |
|  | SFRP5 | cg05937453 | 1.5% | 0.04 |
|  | SFRP5 | cg06692050 | 0.0% | 0.92 |
| **67** | SLC34A2 | cg19616230 | 30.2% | 0.07 |
|  | SLC34A2 | cg21200703 | 16.1% | 0.17 |
| **68** | SLIT2 | cg18972811 | 3.0% | 0.09 |
|  | SLIT2 | cg03742003 | 1.0% | 0.08 |
| **69** | SPINT2 | cg15375239 | 2.0% | 0.02 |
|  | SPINT2 | cg13301014 | 0.5% | 0.10 |
| **70** | SST | cg02164046 | 16.6% | 0.08 |
|  | SST | cg13206017 | 3.5% | 0.17 |
| **71** | TIMP3 | cg05260966 | 1.0% | 0.19 |
|  | TIMP3 | cg22578204 | 1.0% | 0.07 |
|  | TIMP3 | cg05288803 | 0.0% | 0.02 |
|  | TIMP3 | cg24080529 | 0.0% | 0.46 |
| **72** | TM6SF1 | cg14696396 | 35.2% | 0.07 |
|  | TM6SF1 | cg17018527 | 1.0% | 0.06 |
| **73** | TMPRSS2 | cg24901042 | 12.1% | 0.17 |
|  | TMPRSS2 | cg02613803 | 5.0% | 0.06 |
| **74** | UCHL1 | cg24715245 | 7.0% | 0.11 |
|  | UCHL1 | cg08319991 | 1.0% | 0.04 |
| **75** | VHL | cg22782492 | 6.0% | 0.10 |
|  | VHL | cg03509024 | 2.5% | 0.05 |
|  | VHL | cg20916523 | 0.5% | 0.72 |
|  | VHL | cg16869108 | 0.0% | 0.82 |
|  | VHL | cg24092914 | 0.0% | 0.81 |
|  | VHL | cg25539131 | 0.0% | 0.76 |
|  | VHL | cg27226214 | 0.0% | 0.82 |

|  | **Gene Symbol** | **Infinium Probe ID** | **Percentage of 199 Tumors with Probe Difference Values ≥0.35** | **Average Associated Normal β-Value** |
| --- | --- | --- | --- | --- |
|  |  |  |  |  |
| **76** | WIF1 | cg19427610 | 8.0% | 0.05 |
|  | WIF1 | cg20098478 | 0.0% | 0.66 |
| **77** | ZNF447 | cg18888520 | 4.0% | 0.09 |
|  | ZNF447 | cg06243556 | 3.5% | 0.05 |

**Table S3: The 182 Hypermethylated Probes from the HumanMethylation450 BeadChip Cohort.**

|  | **Gene Symbol** | **Chr.** | **Position** | **Probe Name** | **Percentage of 160 Tumors with Difference Values >0.35 (number)** | | **Average Associated Normal β-Value** | **Number of Associated Normals with β-Values >0.25** |
| --- | --- | --- | --- | --- | --- | --- | --- | --- |
| **1** | ATP5G2 | 12 | 52357357 | cg13691247 | 35.63% | (57) | 0.124 | 8 |
|  | ATP5G2 | 12 | 52357461 | cg16264705 | 26.25% | (42) | 0.183 | 38 |
|  | ATP5G2 | 12 | 52357378 | cg23278196 | 26.25% | (42) | 0.086 | 0 |
|  | ATP5G2 | 12 | 52357435 | cg04418091 | 18.75% | (30) | 0.246 | 89 |
| **2** | BMP4 | 14 | 53492675 | cg08162372 | 8.13% | (13) | 0.027 | 0 |
|  | BMP4 | 14 | 53492525 | cg20297199 | 6.88% | (11) | 0.045 | 0 |
| **3** | BNC1 | 15 | 81744887 | cg06523224 | 18.75% | (30) | 0.122 | 3 |
|  | BNC1 | 15 | 81743778 | cg04090392 | 13.75% | (22) | 0.215 | 30 |
|  | BNC1 | 15 | 81743812 | cg19988449 | 13.75% | (22) | 0.093 | 0 |
|  | BNC1 | 15 | 81743349 | cg02318926 | 13.75% | (22) | 0.023 | 0 |
|  | BNC1 | 15 | 81744868 | cg26654798 | 11.25% | (18) | 0.134 | 2 |
|  | BNC1 | 15 | 81744748 | cg18952647 | 11.25% | (18) | 0.090 | 0 |
|  | BNC1 | 15 | 81744884 | cg17124224 | 8.75% | (14) | 0.072 | 0 |
|  | BNC1 | 15 | 81744823 | cg20941110 | 8.13% | (13) | 0.167 | 7 |
|  | BNC1 | 15 | 81744784 | cg09180848 | 8.13% | (13) | 0.074 | 0 |
|  | BNC1 | 15 | 81744779 | cg26429925 | 6.88% | (11) | 0.210 | 35 |
|  | BNC1 | 15 | 81744933 | cg07016258 | 6.25% | (10) | 0.176 | 16 |
|  | BNC1 | 15 | 81744769 | cg14385245 | 6.25% | (10) | 0.114 | 0 |
|  | BNC1 | 15 | 81744072 | cg08939095 | 6.25% | (10) | 0.015 | 0 |
| **4** | BTG3 | 21 | 17907254 | cg02652260 | 5.00% | (8) | 0.023 | 0 |
| **5** | CCDC8 | 19 | 51608581 | cg06747432 | 8.75% | (14) | 0.061 | 1 |
| **6** | CDH13 | 16 | 81218171 | cg08747377 | 7.50% | (12) | 0.043 | 0 |
|  | CDH13 | 16 | 81218228 | cg05374412 | 5.63% | (9) | 0.013 | 0 |
| **7** | COL14A1 | 8 | 121206469 | cg04242021 | 6.88% | (11) | 0.019 | 0 |
| **8** | CORO6 | 17 | 24973987 | cg04568355 | 5.00% | (8) | 0.031 | 0 |
| **9** | CST6 | 11 | 65536102 | cg04327181 | 10.00% | (16) | 0.064 | 0 |
| **10** | DKK1 | 10 | 53744794 | cg08812555 | 24.38% | (39) | 0.022 | 0 |
|  | DKK1 | 10 | 53744085 | cg09445939 | 23.13% | (37) | 0.075 | 0 |
|  | DKK1 | 10 | 53744046 | cg11931116 | 7.50% | (12) | 0.161 | 2 |
|  | DKK1 | 10 | 53744256 | cg27591349 | 7.50% | (12) | 0.097 | 0 |
|  | DKK1 | 10 | 53744749 | cg09465786 | 7.50% | (12) | 0.022 | 0 |
| **11** | DKK2 | 4 | 108176879 | cg13139972 | 26.88% | (43) | 0.040 | 0 |
|  | DKK2 | 4 | 108176811 | cg01962428 | 6.25% | (10) | 0.018 | 0 |
|  | DKK2 | 4 | 108176404 | cg08341316 | 5.00% | (8) | 0.028 | 0 |
| **12** | DKK3 | 11 | 11987414 | cg19867649 | 6.88% | (11) | 0.019 | 0 |
|  | DKK3 | 11 | 11987040 | cg12778215 | 5.63% | (9) | 0.083 | 0 |
|  | DKK3 | 11 | 11986664 | cg13024368 | 5.00% | (8) | 0.154 | 0 |
| **13** | DLEC1 | 3 | 38055639 | cg20684180 | 18.13% | (29) | 0.154 | 10 |
|  | DLEC1 | 3 | 38055646 | cg23881725 | 9.38% | (15) | 0.194 | 27 |
|  | DLEC1 | 3 | 38056190 | cg22884020 | 5.00% | (8) | 0.119 | 0 |
| **14** | EPB41L3 | 18 | 5533548 | cg07352438 | 18.75% | (30) | 0.135 | 34 |
|  | EPB41L3 | 18 | 5534169 | cg23564700 | 13.75% | (22) | 0.051 | 0 |
|  | EPB41L3 | 18 | 5534099 | cg16622495 | 13.13% | (21) | 0.085 | 11 |
|  | EPB41L3 | 18 | 5534031 | cg23847381 | 12.50% | (20) | 0.118 | 21 |
|  | EPB41L3 | 18 | 5533878 | cg19579167 | 11.88% | (19) | 0.118 | 23 |
|  | EPB41L3 | 18 | 5533945 | cg16304950 | 11.88% | (19) | 0.044 | 0 |
|  | EPB41L3 | 18 | 5533801 | cg00027083 | 10.63% | (17) | 0.183 | 58 |
|  | EPB41L3 | 18 | 5532879 | cg26790372 | 6.25% | (10) | 0.075 | 0 |
|  | EPB41L3 | 18 | 5534231 | cg16924702 | 6.25% | (10) | 0.023 | 0 |
|  | EPB41L3 | 18 | 5534153 | cg01673082 | 5.00% | (8) | 0.207 | 25 |
|  | EPB41L3 | 18 | 5534208 | cg06550462 | 5.00% | (8) | 0.139 | 10 |

|  | **Gene Symbol** | **Chr.** | **Position** | **Probe Name** | **Percentage of 160 Tumors with Difference Values >0.35 (number)** | | **Average Associated Normal β-Value** | **Number of Associated Normals with β-Values >0.25** |
| --- | --- | --- | --- | --- | --- | --- | --- | --- |
| **15** | FBN2 | 5 | 127902724 | cg27223047 | 52.50% | (84) | 0.119 | 0 |
|  | FBN2 | 5 | 127901296 | cg12940822 | 40.63% | (65) | 0.022 | 0 |
|  | FBN2 | 5 | 127901182 | cg05209584 | 32.50% | (52) | 0.098 | 0 |
|  | FBN2 | 5 | 127901610 | cg25084878 | 29.38% | (47) | 0.066 | 0 |
|  | FBN2 | 5 | 127902486 | cg05686497 | 22.50% | (36) | 0.014 | 0 |
|  | FBN2 | 5 | 127902362 | cg11965976 | 21.25% | (34) | 0.051 | 0 |
|  | FBN2 | 5 | 127902365 | cg25532099 | 11.88% | (19) | 0.044 | 0 |
|  | FBN2 | 5 | 127902377 | cg01939274 | 10.00% | (16) | 0.050 | 0 |
|  | FBN2 | 5 | 127901879 | cg06993118 | 10.00% | (16) | 0.021 | 0 |
|  | FBN2 | 5 | 127901451 | cg17958315 | 7.50% | (12) | 0.015 | 0 |
| **16** | GATA5 | 20 | 60484980 | cg09339194 | 13.13% | (21) | 0.060 | 0 |
|  | GATA5 | 20 | 60484943 | cg02146001 | 9.38% | (15) | 0.038 | 0 |
|  | GATA5 | 20 | 60484956 | cg23770904 | 8.75% | (14) | 0.023 | 0 |
|  | GATA5 | 20 | 60484736 | cg16714055 | 8.13% | (13) | 0.017 | 0 |
|  | GATA5 | 20 | 60484431 | cg02484469 | 7.50% | (12) | 0.013 | 0 |
|  | GATA5 | 20 | 60484827 | cg08568720 | 6.88% | (11) | 0.023 | 0 |
|  | GATA5 | 20 | 60484416 | cg12664464 | 6.88% | (11) | 0.014 | 0 |
|  | GATA5 | 20 | 60484427 | cg20265733 | 6.25% | (10) | 0.016 | 0 |
|  | GATA5 | 20 | 60484280 | cg14831174 | 5.63% | (9) | 0.065 | 0 |
|  | GATA5 | 20 | 60484424 | cg13845982 | 5.63% | (9) | 0.019 | 0 |
|  | GATA5 | 20 | 60484743 | cg11982072 | 5.63% | (9) | 0.018 | 0 |
|  | GATA5 | 20 | 60484309 | cg16919517 | 5.63% | (9) | 0.017 | 0 |
|  | GATA5 | 20 | 60484434 | cg14980983 | 5.63% | (9) | 0.014 | 0 |
|  | GATA5 | 20 | 60484712 | cg24320612 | 5.00% | (8) | 0.014 | 0 |
| **17** | GNB4 | 3 | 180651492 | cg12872693 | 15.00% | (24) | 0.051 | 0 |
|  | GNB4 | 3 | 180651454 | cg10013716 | 13.75% | (22) | 0.075 | 0 |
|  | GNB4 | 3 | 180651946 | cg25823419 | 8.75% | (14) | 0.013 | 0 |
|  | GNB4 | 3 | 180652230 | cg20788479 | 7.50% | (12) | 0.070 | 0 |
| **18** | GREM1 | 15 | 30797828 | cg21296230 | 13.75% | (22) | 0.141 | 0 |
|  | GREM1 | 15 | 30797691 | cg08495115 | 10.00% | (16) | 0.016 | 0 |
|  | GREM1 | 15 | 30798458 | cg26727488 | 6.88% | (11) | 0.019 | 0 |
| **19** | GSTP1 | 11 | 67107847 | cg04920951 | 12.50% | (20) | 0.055 | 2 |
|  | GSTP1 | 11 | 67108066 | cg06928838 | 11.88% | (19) | 0.144 | 23 |
|  | GSTP1 | 11 | 67107849 | cg26250609 | 11.25% | (18) | 0.190 | 36 |
|  | GSTP1 | 11 | 67108184 | cg09038676 | 10.63% | (17) | 0.186 | 43 |
| **20** | GUCY2D | 17 | 7847572 | cg04157161 | 28.13% | (45) | 0.242 | 66 |
|  | GUCY2D | 17 | 7846823 | cg25465406 | 13.75% | (22) | 0.173 | 4 |
| **21** | HOXC13 | 12 | 52619590 | cg07892422 | 7.50% | (12) | 0.188 | 6 |
|  | HOXC13 | 12 | 52619259 | cg16856286 | 5.00% | (8) | 0.023 | 0 |
| **22** | KLHL35 | 11 | 74819463 | cg12001148 | 15.00% | (24) | 0.073 | 0 |
|  | KLHL35 | 11 | 74817384 | cg10909185 | 5.00% | (8) | 0.201 | 43 |
| **23** | KRT19 | 17 | 36937650 | cg11462865 | 8.13% | (13) | 0.067 | 0 |
|  | KRT19 | 17 | 36938070 | cg02893823 | 6.88% | (11) | 0.032 | 0 |
| **24** | LOXL1 | 15 | 72005974 | cg22590761 | 6.25% | (10) | 0.179 | 20 |
| **25** | MGMT | 10 | 131155049 | cg02022136 | 7.50% | (12) | 0.043 | 0 |
| **26** | OVOL1 | 11 | 65310618 | cg20909686 | 18.75% | (30) | 0.036 | 0 |
|  | OVOL1 | 11 | 65310751 | cg08296831 | 10.00% | (16) | 0.017 | 0 |
| **27** | PCDH8 | 13 | 52319689 | cg05336395 | 62.50% | (100) | 0.134 | 11 |
|  | PCDH8 | 13 | 52320692 | cg27360326 | 49.38% | (79) | 0.018 | 0 |
|  | PCDH8 | 13 | 52318934 | cg08136772 | 45.00% | (72) | 0.104 | 1 |
|  | PCDH8 | 13 | 52317964 | cg07847863 | 27.50% | (44) | 0.034 | 0 |
|  | PCDH8 | 13 | 52320778 | cg19712603 | 26.88% | (43) | 0.022 | 0 |
|  | PCDH8 | 13 | 52318387 | cg09813525 | 24.38% | (39) | 0.021 | 0 |
|  | PCDH8 | 13 | 52320872 | cg16027761 | 21.88% | (35) | 0.065 | 0 |
|  | PCDH8 | 13 | 52320781 | cg22763718 | 16.88% | (27) | 0.020 | 0 |
|  | PCDH8 | 13 | 52320382 | cg20366906 | 13.13% | (21) | 0.235 | 49 |
|  | PCDH8 | 13 | 52320475 | cg14171414 | 6.25% | (10) | 0.176 | 0 |
|  | PCDH8 | 13 | 52318136 | cg06460717 | 5.00% | (8) | 0.102 | 0 |

|  | **Gene Symbol** | **Chr.** | **Position** | **Probe Name** | **Percentage of 160 Tumors with Difference Values >0.35 (number)** | | **Average Associated Normal β-Value** | **Number of Associated Normals with β-Values >0.25** |
| --- | --- | --- | --- | --- | --- | --- | --- | --- |
| **28** | PDLIM4 | 5 | 131621160 | cg02033258 | 31.88% | (51) | 0.141 | 10 |
|  | PDLIM4 | 5 | 131621158 | cg22638593 | 14.38% | (23) | 0.225 | 84 |
| **29** | PTGS2 | 1 | 184915953 | cg13986130 | 6.25% | (10) | 0.038 | 0 |
| **30** | QPCT | 2 | 37425236 | cg08786077 | 15.00% | (24) | 0.037 | 0 |
|  | QPCT | 2 | 37425233 | cg15162392 | 14.38% | (23) | 0.030 | 0 |
|  | QPCT | 2 | 37425181 | cg22629987 | 13.13% | (21) | 0.157 | 2 |
|  | QPCT | 2 | 37425200 | cg20647735 | 11.25% | (18) | 0.053 | 0 |
|  | QPCT | 2 | 37425191 | cg09839443 | 6.88% | (11) | 0.063 | 0 |
| **31** | RASSF5 | 1 | 204748001 | cg18328206 | 9.38% | (15) | 0.036 | 0 |
| **32** | ROBO1 | 3 | 79898538 | cg21865845 | 27.50% | (44) | 0.175 | 9 |
|  | ROBO1 | 3 | 79898408 | cg04562217 | 12.50% | (20) | 0.029 | 0 |
|  | ROBO1 | 3 | 79898329 | cg11980129 | 9.38% | (15) | 0.047 | 0 |
| **33** | RPRM | 2 | 154042897 | cg26649384 | 16.88% | (27) | 0.025 | 0 |
|  | RPRM | 2 | 154043260 | cg06674731 | 16.25% | (26) | 0.049 | 0 |
|  | RPRM | 2 | 154043430 | cg17942639 | 15.63% | (25) | 0.012 | 0 |
|  | RPRM | 2 | 154042962 | cg27420236 | 9.38% | (15) | 0.065 | 0 |
|  | RPRM | 2 | 154042696 | cg14765646 | 8.75% | (14) | 0.029 | 0 |
|  | RPRM | 2 | 154043445 | cg22278296 | 5.63% | (9) | 0.079 | 0 |
|  | RPRM | 2 | 154043630 | cg19861138 | 5.00% | (8) | 0.016 | 0 |
|  | RPRM | 2 | 154043632 | cg00522056 | 5.00% | (8) | 0.011 | 0 |
| **34** | SCUBE3 | 6 | 35290486 | cg00347904 | 6.25% | (10) | 0.058 | 0 |
| **35** | SFRP1 | 8 | 41286147 | cg24319902 | 33.75% | (54) | 0.033 | 0 |
|  | SFRP1 | 8 | 41286270 | cg10406295 | 18.13% | (29) | 0.085 | 0 |
|  | SFRP1 | 8 | 41286266 | cg17816908 | 14.38% | (23) | 0.041 | 0 |
|  | SFRP1 | 8 | 41286264 | cg21517947 | 13.75% | (22) | 0.020 | 0 |
|  | SFRP1 | 8 | 41286244 | cg01495122 | 11.88% | (19) | 0.062 | 0 |
|  | SFRP1 | 8 | 41285895 | cg22418909 | 9.38% | (15) | 0.079 | 0 |
| **36** | SFRP2 | 4 | 154929949 | cg22178613 | 18.75% | (30) | 0.047 | 0 |
|  | SFRP2 | 4 | 154929985 | cg23207990 | 12.50% | (20) | 0.026 | 0 |
|  | SFRP2 | 4 | 154930276 | cg20881942 | 9.38% | (15) | 0.133 | 7 |
|  | SFRP2 | 4 | 154929823 | cg25775322 | 6.25% | (10) | 0.113 | 0 |
|  | SFRP2 | 4 | 154930246 | cg00082664 | 6.25% | (10) | 0.055 | 0 |
|  | SFRP2 | 4 | 154929803 | cg03202804 | 5.63% | (9) | 0.056 | 0 |
|  | SFRP2 | 4 | 154929871 | cg23121156 | 5.00% | (8) | 0.047 | 0 |
|  | SFRP2 | 4 | 154929868 | cg05164933 | 5.00% | (8) | 0.031 | 0 |
| **37** | SLC34A2 | 4 | 25266538 | cg27513574 | 26.88% | (43) | 0.017 | 0 |
|  | SLC34A2 | 4 | 25266527 | cg19616230 | 25.63% | (41) | 0.130 | 1 |
|  | SLC34A2 | 4 | 25266512 | cg07578695 | 24.38% | (39) | 0.028 | 0 |
|  | SLC34A2 | 4 | 25266479 | cg03738352 | 23.13% | (37) | 0.020 | 0 |
|  | SLC34A2 | 4 | 25266463 | cg19766441 | 18.75% | (30) | 0.020 | 0 |
|  | SLC34A2 | 4 | 25266258 | cg09752627 | 18.75% | (30) | 0.020 | 0 |
|  | SLC34A2 | 4 | 25266501 | cg01493016 | 17.50% | (28) | 0.101 | 1 |
| **38** | SLIT2 | 4 | 19865939 | cg03260566 | 5.00% | (8) | 0.087 | 0 |
|  | SLIT2 | 4 | 19864180 | cg13078140 | 5.00% | (8) | 0.036 | 0 |
| **39** | SST | 3 | 188870842 | cg02164046 | 24.38% | (39) | 0.040 | 0 |
|  | SST | 3 | 188870822 | cg16927040 | 18.13% | (29) | 0.041 | 0 |
|  | SST | 3 | 188870693 | cg15347189 | 9.38% | (15) | 0.104 | 0 |
| **40** | TM6SF1 | 15 | 81567424 | cg26460092 | 36.88% | (59) | 0.016 | 0 |
|  | TM6SF1 | 15 | 81567426 | cg03063639 | 33.13% | (53) | 0.018 | 0 |
|  | TM6SF1 | 15 | 81567450 | cg14696396 | 29.38% | (47) | 0.021 | 0 |
|  | TM6SF1 | 15 | 81567273 | cg11167100 | 14.38% | (23) | 0.015 | 0 |
|  | TM6SF1 | 15 | 81567275 | cg08452658 | 10.00% | (16) | 0.014 | 0 |

|  | **Gene Symbol** | **Chr.** | **Position** | **Probe Name** | **Percentage of 160 Tumors with Difference Values >0.35 (number)** | | **Average Associated Normal β-Value** | **Number of Associated Normals with β-Values >0.25** |
| --- | --- | --- | --- | --- | --- | --- | --- | --- |
| **41** | TMPRSS2 | 21 | 41800944 | cg16084872 | 18.75% | (30) | 0.062 | 0 |
|  | TMPRSS2 | 21 | 41802427 | cg26309194 | 16.88% | (27) | 0.126 | 2 |
|  | TMPRSS2 | 21 | 41801001 | cg02613803 | 7.50% | (12) | 0.043 | 0 |
|  | TMPRSS2 | 21 | 41802424 | cg24901042 | 5.00% | (8) | 0.172 | 5 |
| **42** | UCHL1 | 4 | 40953667 | cg07068756 | 11.88% | (19) | 0.020 | 0 |
|  | UCHL1 | 4 | 40953692 | cg16142306 | 11.25% | (18) | 0.096 | 0 |
|  | UCHL1 | 4 | 40953801 | cg16026922 | 10.63% | (17) | 0.200 | 18 |
|  | UCHL1 | 4 | 40954040 | cg08319991 | 6.88% | (11) | 0.052 | 0 |
|  | UCHL1 | 4 | 40953551 | cg24715245 | 5.63% | (9) | 0.212 | 16 |
| **43** | VHL | 3 | 10158328 | cg15267345 | 5.00% | (8) | 0.054 | 0 |
| **44** | WIF1 | 12 | 63801543 | cg26397188 | 17.50% | (28) | 0.108 | 0 |
|  | WIF1 | 12 | 63801737 | cg21383810 | 15.00% | (24) | 0.021 | 0 |
|  | WIF1 | 12 | 63801288 | cg03509412 | 14.38% | (23) | 0.098 | 0 |
|  | WIF1 | 12 | 63801557 | cg24166864 | 13.75% | (22) | 0.111 | 0 |
| **45** | ZSCAN18 | 19 | 63321713 | cg14231297 | 55.00% | (88) | 0.074 | 1 |
|  | ZSCAN18 | 19 | 63321531 | cg10659886 | 30.63% | (49) | 0.117 | 9 |
|  | ZSCAN18 | 19 | 63321779 | cg17408527 | 20.63% | (33) | 0.176 | 6 |
|  | ZSCAN18 | 19 | 63321787 | cg18693673 | 18.75% | (30) | 0.100 | 0 |
|  | ZSCAN18 | 19 | 63301414 | cg25784220 | 16.25% | (26) | 0.013 | 0 |
|  | ZSCAN18 | 19 | 63301772 | cg14972743 | 15.00% | (24) | 0.066 | 0 |
|  | ZSCAN18 | 19 | 63301430 | cg22721334 | 14.38% | (23) | 0.015 | 0 |

**Table S4: The Number of Hypermethylated Probes out of all the CpG Island Probes for Each Hypermethylated Gene from the HumanMethylation450 BeadChip Cohort.**

| **Gene Name** | **No. of Hypermethylated Probes from Total CpG Island Probes (%)** | | **Average Frequency of Hypermethylated Probes** |
| --- | --- | --- | --- |
| ATP5G2 | 4 out of 10 | (40.0%) | 26.7% |
| BMP4 | 2 out of 14 | (14.3%) | 7.5% |
| BNC1 | 13 out of 20 | (65.0%) | 10.2% |
| BTG3 | 1 out of 9 | (11.1%) | n/a |
| CCDC8 | 1 out of 1 | (100.0%) | n/a |
| CDH13 | 2 out of 7 | (28.6%) | 7.2% |
| COL14A1 | 1 out of 6 | (16.7%) | n/a |
| CORO6 | 1 out of 7 | (14.3%) | n/a |
| CST6 | 1 out of 5 | (20.0%) | n/a |
| DKK1 | 5 out of 7 | (71.4%) | 14.0% |
| DKK2 | 3 out of 5 | (60.0%) | 12.7% |
| DKK3 | 3 out of 13 | (23.1%) | 5.8% |
| DLEC1 | 3 out of 6 | (50.0%) | 10.8% |
| EPB41L3 | 11 out of 13 | (84.6%) | 10.5% |
| FBN2 | 10 out of 10 | (100.0%) | 23.8% |
| GATA5 | 14 out of 25 | (56.0%) | 7.1% |
| GNB4 | 4 out of 7 | (57.1%) | 11.3% |
| GREM1 | 3 out of 7 | (42.9%) | 10.2% |
| GSTP1 | 4 out of 5 | (80.0%) | 11.6% |
| GUCY2D | 2 out of 8 | (25.0%) | 20.9% |
| HOXC13 | 2 out of 9 | (22.2%) | 6.3% |
| KLHL35 | 2 out of 7 | (28.6%) | 10.0% |
| KRT19 | 2 out of 3 | (66.7%) | 7.5% |
| LOXL1 | 1 out of 11 | (9.1%) | n/a |
| MGMT | 1 out of 14 | (7.1%) | n/a |
| OVOL1 | 2 out of 11 | (18.2%) | 14.4% |
| PCDH8 | 11 out of 13 | (84.6%) | 27.2% |
| PDLIM4 | 2 out of 2 | (100.0%) | 23.1% |
| PTGS2 | 1 out of 10 | (10.0%) | n/a |
| QPCT | 5 out of 8 | (62.5%) | 12.1% |
| RASSF5 | 1 out of 16 | (6.3%) | n/a |
| ROBO1 | 3 out of 7 | (42.9%) | 16.5% |
| RPRM | 8 out of 12 | (66.7%) | 10.3% |
| SCUBE3 | 1 out of 6 | (16.7%) | n/a |
| SFRP1 | 6 out of 8 | (75.0%) | 16.9% |
| SFRP2 | 8 out of 19 | (42.1%) | 8.6% |
| SLC34A2 | 7 out of 7 | (100.0%) | 22.1% |
| SLIT2 | 2 out of 12 | (16.7%) | 5.0% |
| SST | 3 out of 3 | (100.0%) | 17.3% |
| TM6SF1 | 5 out of 7 | (71.4%) | 24.8% |
| TMPRSS2 | 4 out of 10 | (40.0%) | 12.0% |
| UCHL1 | 5 out of 7 | (71.4%) | 9.3% |
| VHL | 1 out of 3 | (33.3%) | n/a |
| WIF1 | 4 out of 6 | (66.7%) | 15.2% |
| ZSCAN18 | 7 out of 18 | (38.9%) | 24.4% |

**Table S5: Clinical Information for the HumanMethylation27 BeadChip Cohort and Hypermethylation Status for Significant Genes.**

| TCGA Sample Code | Gender | Maximum Tumor Dimension (cm) | Tumor Grade | Tumor Stage | Vital Status | Max Follwup | BNC1 cg18952647 | PCDH8 cg20366906 | SCUBE3 cg21604042 | SFRP1 cg13398291 | SFRP1 cg15839448 | SFRP1 cg22418909 |
| --- | --- | --- | --- | --- | --- | --- | --- | --- | --- | --- | --- | --- |
| TCGA-A3-3308 | FEMALE | 5.0 | G2 | T3b | LIVING | 16 | 0 | 0 | 0 | 0 | 0 | 0 |
| TCGA-A3-3311 | MALE | 5.5 | G2 | T1 | DECEASED | 1190 | 0 | 0 | 1 | 1 | 0 | 0 |
| TCGA-A3-3316 | MALE | 9.0 | G3 | T2 | LIVING | 1493 | 0 | 1 | 1 | 0 | 1 | 1 |
| TCGA-A3-3317 | MALE | 12.0 | G2 | T2 | LIVING | 1491 | 1 | 1 | 0 | 0 | 0 | 0 |
| TCGA-A3-3319 | MALE | 6.0 | G2 | T1b | LIVING | 1130 | 0 | 1 | 0 | 0 | 0 | 0 |
| TCGA-A3-3320 | FEMALE | 5.0 | G1 | T1b | LIVING | 1508 | 0 | 0 | 0 | 0 | 0 | 0 |
| TCGA-A3-3322 | MALE | 3.0 | G2 | T1a | LIVING | 1477 | 0 | 0 | 0 | 0 | 0 | 0 |
| TCGA-A3-3323 | MALE | 5.0 | G1 | T1b | LIVING | 1105 | 0 | 0 | 0 | 0 | 1 | 0 |
| TCGA-A3-3324 | MALE | 6.3 | G3 | T1b | LIVING | 1186 | 0 | 0 | 0 | 0 | 0 | 1 |
| TCGA-A3-3325 | MALE | 3.3 | G2 | T1a | LIVING | 751 | 0 | 0 | 0 | 0 | 0 | 0 |
| TCGA-A3-3326 | MALE | 2.7 | G1 | T1a | LIVING | 1137 | 0 | 0 | 0 | 0 | 0 | 0 |
| TCGA-A3-3329 | MALE | 6.5 | G2 | T1b | LIVING | 706 | 0 | 0 | 0 | 0 | 0 | 0 |
| TCGA-A3-3331 | FEMALE | 3.7 | G2 | T1 | LIVING | 1485 | 0 | 0 | 0 | 0 | 0 | 0 |
| TCGA-A3-3343 | MALE | 8.0 | G3 | T2 | LIVING | 944 | 0 | 0 | 1 | 0 | 0 | 0 |
| TCGA-A3-3346 | MALE | 5.2 | G3 | T1b | DECEASED | 137 | 1 | 1 | 0 | 1 | 0 | 1 |
| TCGA-A3-3347 | FEMALE | 6.5 | G2 | T1b | LIVING | 1610 | 1 | 1 | 1 | 1 | 1 | 1 |
| TCGA-A3-3349 | FEMALE | 5.0 | G2 | T1b | LIVING | 1385 | 0 | 0 | 0 | 0 | 0 | 0 |
| TCGA-A3-3351 | MALE | 8.4 | G2 | T2a | LIVING | 910 | 0 | 0 | 0 | 0 | 0 | 0 |
| TCGA-A3-3352 | MALE | 5.5 | G3 | T3a | DECEASED | 561 | 1 | 1 | 1 | 0 | 0 | 1 |
| TCGA-A3-3359 | FEMALE | 4.0 | G2 | T1a | LIVING | 1286 | 0 | 0 | 0 | 0 | 0 | 0 |
| TCGA-A3-3362 | FEMALE | 3.3 | G2 | T1a | LIVING | 1559 | 0 | 0 | 0 | 0 | 0 | 0 |
| TCGA-A3-3363 | MALE | 10.7 | G2 | T2 | LIVING | 319 | 0 | 0 | 0 | 0 | 0 | 0 |
| TCGA-A3-3365 | MALE | 3.0 | G2 | T1a | LIVING | 872 | 0 | 0 | 0 | 0 | 0 | 0 |
| TCGA-A3-3372 | MALE | 6.9 | G2 | T3 | LIVING | 735 | 0 | 0 | 0 | 0 | 0 | 0 |
| TCGA-A3-3374 | MALE | 5.5 | G3 | T1 | LIVING | 630 | 0 | 0 | 0 | 0 | 0 | 0 |
| TCGA-A3-3378 | MALE | 3.0 | G2 | T1 | LIVING | 567 | 0 | 0 | 0 | 0 | 0 | 0 |
| TCGA-A3-3380 | MALE | 5.2 | G3 | T1b | LIVING | 574 | 0 | 0 | 0 | 0 | 0 | 0 |
| TCGA-A3-3383 | MALE | 5.0 | G2 | T1 | LIVING | 861 | 0 | 0 | 0 | 0 | 0 | 0 |
| TCGA-B0-4833 | FEMALE | 5.0 | G2 | T1b | DECEASED | 2385 | 0 | 1 | 0 | 0 | 0 | 0 |

| TCGA Sample Code | Gender | Maximum Tumor Dimension (cm) | Tumor Grade | Tumor Stage | Vital Status | Max Follwup | BNC1 cg18952647 | PCDH8 cg20366906 | SCUBE3 cg21604042 | SFRP1 cg13398291 | SFRP1 cg15839448 | SFRP1 cg22418909 |
| --- | --- | --- | --- | --- | --- | --- | --- | --- | --- | --- | --- | --- |
| TCGA-B0-4836 | MALE | 2.8 | G3 | T3b | DECEASED | 1238 | 1 | 1 | 1 | 1 | 0 | 1 |
| TCGA-B0-4837 | MALE | 6.0 | G3 | T1b | DECEASED | 1378 | 1 | 0 | 1 | 0 | 0 | 0 |
| TCGA-B0-4838 | FEMALE | 4.2 | G3 | T1b | DECEASED | 834 | 0 | 0 | 0 | 0 | 0 | 0 |
| TCGA-B0-4839 | FEMALE | 4.5 | G2 | T1b | DECEASED | 1638 | 0 | 0 | 0 | 0 | 0 | 0 |
| TCGA-B0-5075 | FEMALE | 2.0 | G2 | T3a | DECEASED | 637 | 1 | 0 | 0 | 0 | 0 | 0 |
| TCGA-B0-5077 | MALE | 3.8 | G3 | T1a | DECEASED | 1316 | 0 | 0 | 0 | 0 | 0 | 0 |
| TCGA-B0-5081 | FEMALE | 5.5 | G2 | T3b | DECEASED | 362 | 1 | 0 | 0 | 0 | 0 | 1 |
| TCGA-B0-5085 | FEMALE | 6.8 | G3 | T3a | DECEASED | 769 | 0 | 0 | 1 | 0 | 0 | 0 |
| TCGA-B0-5088 | MALE | 5.1 | G3 | T1b | DECEASED | 563 | 0 | 0 | 0 | 0 | 0 | 0 |
| TCGA-B2-3924 | MALE | 4.2 | G2 | T1b | LIVING | 371 | 0 | 0 | 0 | 0 | 0 | 0 |
| TCGA-B2-4098 | FEMALE | 5.0 | G2 | T1b | DECEASED | 50 | 0 | 0 | 1 | 0 | 0 | 0 |
| TCGA-B2-4099 | MALE | 3.0 | G3 | T1a | LIVING | 373 | 0 | 0 | 0 | 1 | 0 | 0 |
| TCGA-B2-4102 | MALE | 5.0 | G2 | T1b | LIVING | 202 | 0 | 0 | 0 | 0 | 0 | 0 |
| TCGA-B8-4143 | FEMALE | 10.0 | G3 | T3a | DECEASED | 709 | 1 | 1 | 0 | 1 | 1 | 1 |
| TCGA-B8-4154 | FEMALE | 2.0 | G2 | T1a | LIVING | 749 | 1 | 0 | 0 | 0 | 0 | 0 |
| TCGA-BP-4158 | MALE | 7.0 | G2 | T1b | LIVING | 3377 | 0 | 0 | 0 | 0 | 0 | 0 |
| TCGA-BP-4159 | MALE | 7.0 | G2 | T1b | DECEASED | 2600 | 0 | 0 | 0 | 0 | 0 | 0 |
| TCGA-BP-4160 | MALE | 4.5 | G2 | T3a | LIVING | 2881 | 0 | 0 | 0 | 0 | 0 | 0 |
| TCGA-BP-4161 | MALE | 5.5 | G3 | T1b | LIVING | 2746 | 0 | 0 | 1 | 0 | 0 | 0 |
| TCGA-BP-4162 | FEMALE | 4.5 | G2 | T1b | LIVING | 3074 | 0 | 0 | 0 | 0 | 0 | 0 |
| TCGA-BP-4163 | FEMALE | 8.0 | G3 | T3a | LIVING | 2839 | 0 | 0 | 0 | 0 | 0 | 0 |
| TCGA-BP-4164 | FEMALE | 13.5 | G2 | T3a | DECEASED | 992 | 0 | 0 | 0 | 0 | 0 | 0 |
| TCGA-BP-4165 | FEMALE | 5.5 | G1 | T1b | LIVING | 3037 | 0 | 0 | 0 | 0 | 0 | 0 |
| TCGA-BP-4166 | MALE | 5.0 | G3 | T3a | LIVING | 13 | 0 | 0 | 0 | 0 | 0 | 0 |
| TCGA-BP-4167 | MALE | 4.5 | G2 | T3a | LIVING | 2718 | 0 | 0 | 0 | 0 | 0 | 0 |
| TCGA-BP-4169 | FEMALE | 9.0 | G2 | T2 | DECEASED | 701 | 0 | 0 | 0 | 0 | 0 | 0 |
| TCGA-BP-4170 | FEMALE | 5.0 | G2 | T1b | DECEASED | 2343 | 0 | 0 | 0 | 0 | 0 | 0 |
| TCGA-BP-4173 | MALE | 9.5 | G3 | T2 | LIVING | 1893 | 0 | 0 | 0 | 0 | 0 | 0 |
| TCGA-BP-4174 | MALE | 9.4 | G3 | T2 | LIVING | 1879 | 0 | 0 | 0 | 0 | 0 | 0 |
| TCGA-BP-4176 | MALE | 4.5 | G2 | T1b | LIVING | 1955 | 1 | 0 | 0 | 0 | 0 | 0 |
| TCGA-BP-4325 | FEMALE | 4.5 | G2 | T1b | LIVING | 2964 | 0 | 0 | 0 | 0 | 0 | 0 |

| TCGA Sample Code | Gender | Maximum Tumor Dimension (cm) | Tumor Grade | Tumor Stage | Vital Status | Max Follwup | BNC1 cg18952647 | PCDH8 cg20366906 | SCUBE3 cg21604042 | SFRP1 cg13398291 | SFRP1 cg15839448 | SFRP1 cg22418909 |
| --- | --- | --- | --- | --- | --- | --- | --- | --- | --- | --- | --- | --- |
| TCGA-BP-4326 | FEMALE | 6.3 | G2 | T1b | LIVING | 1624 | 1 | 0 | 0 | 0 | 0 | 0 |
| TCGA-BP-4327 | FEMALE | 7.5 | G2 | T2 | DECEASED | 108 | 1 | 0 | 0 | 0 | 0 | 1 |
| TCGA-BP-4329 | MALE | 7.5 | G2 | T3a | LIVING | 845 | 0 | 0 | 0 | 1 | 0 | 0 |
| TCGA-BP-4330 | FEMALE | 12.0 | G2 | T3a | LIVING | 1888 | 0 | 0 | 0 | 0 | 0 | 0 |
| TCGA-BP-4331 | MALE | 3.7 | G2 | T1a | DECEASED | 2453 | 0 | 0 | 0 | 0 | 0 | 0 |
| TCGA-BP-4332 | MALE | 4.0 | G2 | T3a | LIVING | 1147 | 0 | 0 | 0 | 0 | 0 | 0 |
| TCGA-BP-4334 | FEMALE | 5.5 | G3 | T3a | DECEASED | 460 | 0 | 1 | 0 | 0 | 0 | 0 |
| TCGA-BP-4337 | FEMALE | 6.8 | G4 | T3b | DECEASED | 2 | 0 | 0 | 0 | 0 | 0 | 0 |
| TCGA-BP-4338 | MALE | 5.5 | G3 | T1b | LIVING | 2859 | 1 | 1 | 1 | 0 | 0 | 0 |
| TCGA-BP-4340 | FEMALE | 4.5 | G2 | T1b | DECEASED | 562 | 0 | 0 | 0 | 0 | 0 | 0 |
| TCGA-BP-4341 | MALE | 7.3 | G2 | T3a | DECEASED | 1589 | 0 | 0 | 0 | 0 | 0 | 0 |
| TCGA-BP-4342 | MALE | 11.0 | G3 | T2 | DECEASED | 2256 | 1 | 1 | 1 | 1 | 1 | 1 |
| TCGA-BP-4343 | MALE | 7.0 | G3 | T3a | DECEASED | 1912 | 0 | 0 | 0 | 0 | 0 | 1 |
| TCGA-BP-4345 | MALE | 5.5 | G3 | T3b | LIVING | 1516 | 0 | 0 | 0 | 0 | 0 | 0 |
| TCGA-BP-4346 | MALE | 6.9 | G3 | T3b | DECEASED | 1493 | 0 | 0 | 0 | 0 | 0 | 0 |
| TCGA-BP-4347 | MALE | 3.0 | G2 | T3b | LIVING | 1367 | 0 | 0 | 0 | 0 | 0 | 0 |
| TCGA-BP-4349 | FEMALE | 1.5 | G2 | T1a | LIVING | 372 | 0 | 0 | 0 | 0 | 0 | 0 |
| TCGA-BP-4351 | FEMALE | 16.0 | G2 | T3a | LIVING | 969 | 0 | 0 | 0 | 0 | 0 | 0 |
| TCGA-BP-4352 | FEMALE | 10.0 | G4 | T3b | DECEASED | 344 | 1 | 1 | 1 | 1 | 1 | 1 |
| TCGA-BP-4353 | MALE | 3.9 | G2 | T1 | DECEASED | 375 | 0 | 0 | 0 | 0 | 0 | 0 |
| TCGA-BP-4354 | MALE | 16.5 | G4 | T4 | DECEASED | 1034 | 1 | 0 | 0 | 0 | 0 | 1 |
| TCGA-BP-4355 | FEMALE | 13.0 | G4 | T3a | DECEASED | 953 | 0 | 0 | 0 | 0 | 0 | 0 |
| TCGA-BP-4756 | FEMALE | 6.5 | G2 | T1b | LIVING | 374 | 0 | 0 | 0 | 0 | 0 | 0 |
| TCGA-BP-4758 | MALE | 4.0 | G2 | T1a | LIVING | 2208 | 0 | 0 | 0 | 0 | 0 | 0 |
| TCGA-BP-4759 | MALE | 3.0 | G2 | T1a | LIVING | 2372 | 0 | 0 | 0 | 0 | 0 | 0 |
| TCGA-BP-4761 | MALE | 12.0 | G4 | T3a | LIVING | 902 | 1 | 1 | 1 | 0 | 0 | 1 |
| TCGA-BP-4762 | MALE | 1.1 | G3 | T1a | DECEASED | 1343 | 0 | 0 | 0 | 0 | 0 | 0 |
| TCGA-BP-4763 | FEMALE | 2.2 | G2 | T1a | DECEASED | 1270 | 0 | 0 | 0 | 0 | 0 | 0 |
| TCGA-BP-4765 | MALE | 3.2 | G2 | T1a | LIVING | 2184 | 0 | 0 | 0 | 0 | 0 | 0 |
| TCGA-BP-4766 | FEMALE | 3.9 | G3 | T1a | LIVING | 1462 | 0 | 0 | 0 | 1 | 0 | 0 |
| TCGA-BP-4768 | FEMALE | 3.0 | G2 | T1a | LIVING | 400 | 0 | 0 | 0 | 0 | 0 | 0 |

| TCGA Sample Code | Gender | Maximum Tumor Dimension (cm) | Tumor Grade | Tumor Stage | Vital Status | Max Follwup | BNC1 cg18952647 | PCDH8 cg20366906 | SCUBE3 cg21604042 | SFRP1 cg13398291 | SFRP1 cg15839448 | SFRP1 cg22418909 |
| --- | --- | --- | --- | --- | --- | --- | --- | --- | --- | --- | --- | --- |
| TCGA-BP-4771 | MALE | 5.7 | G4 | T3a | DECEASED | 161 | 0 | 0 | 0 | 0 | 0 | 0 |
| TCGA-BP-4774 | FEMALE | 3.6 | G2 | T1a | LIVING | 1884 | 0 | 0 | 0 | 0 | 0 | 0 |
| TCGA-BP-4775 | FEMALE | 2.9 | G2 | T1a | LIVING | 1842 | 0 | 0 | 0 | 0 | 0 | 0 |
| TCGA-BP-4776 | MALE | 2.7 | G2 | T1a | LIVING | 410 | 0 | 0 | 0 | 0 | 0 | 0 |
| TCGA-BP-4777 | MALE | 1.9 | G3 | T1a | LIVING | 1731 | 0 | 0 | 0 | 0 | 0 | 0 |
| TCGA-BP-4781 | MALE | 3.2 | G3 | T1a | LIVING | 2080 | 1 | 0 | 0 | 0 | 0 | 0 |
| TCGA-BP-4787 | FEMALE | 9.5 | G4 | T3a | DECEASED | 479 | 1 | 0 | 0 | 1 | 1 | 0 |
| TCGA-BP-4789 | MALE | 3.4 | G2 | T1a | LIVING | 1489 | 0 | 0 | 0 | 0 | 0 | 0 |
| TCGA-BP-4790 | MALE | 3.5 | G2 | T1a | DECEASED | 1111 | 0 | 0 | 0 | 0 | 0 | 0 |
| TCGA-BP-4797 | MALE | 4.5 | G3 | T3b | LIVING | 1107 | 0 | 0 | 0 | 0 | 0 | 0 |
| TCGA-BP-4798 | MALE | 9.5 | G4 | T3b | DECEASED | 334 | 1 | 1 | 0 | 0 | 0 | 1 |
| TCGA-BP-4799 | MALE | 6.0 | G3 | T3b | DECEASED | 1133 | 1 | 1 | 1 | 1 | 1 | 1 |
| TCGA-BP-4803 | MALE | 4.8 | G3 | T3a | LIVING | 203 | 0 | 0 | 0 | 0 | 0 | 0 |
| TCGA-BP-4804 | MALE | 5.1 | G2 | T1b | LIVING | 1459 | 0 | 0 | 0 | 0 | 0 | 0 |
| TCGA-BP-4807 | MALE | 1.9 | G3 | T1a | LIVING | 389 | 0 | 0 | 0 | 0 | 0 | 0 |
| TCGA-BP-4959 | MALE | 4.1 | G3 | T1b | LIVING | 2660 | 0 | 0 | 0 | 0 | 0 | 0 |
| TCGA-BP-4960 | MALE | 10.5 | G3 | T2 | LIVING | 2172 | 1 | 0 | 0 | 0 | 0 | 0 |
| TCGA-BP-4961 | MALE | 3.0 | G2 | T1a | LIVING | 1935 | 0 | 0 | 0 | 0 | 0 | 1 |
| TCGA-BP-4962 | MALE | 8.5 | G2 | T2 | LIVING | 1785 | 0 | 0 | 0 | 0 | 0 | 0 |
| TCGA-BP-4963 | MALE | 6.5 | G3 | T1b | LIVING | 1834 | 0 | 1 | 0 | 0 | 0 | 1 |
| TCGA-BP-4964 | FEMALE | 3.9 | G2 | T1a | LIVING | 1862 | 0 | 1 | 0 | 0 | 0 | 0 |
| TCGA-BP-4967 | MALE | 5.5 | G2 | T3a | LIVING | 204 | 0 | 0 | 1 | 0 | 0 | 0 |
| TCGA-BP-4968 | MALE | 5.5 | G3 | T1b | LIVING | 1792 | 0 | 0 | 0 | 0 | 0 | 0 |
| TCGA-BP-4969 | FEMALE | 1.9 | G2 | T1a | LIVING | 1794 | 0 | 0 | 0 | 0 | 0 | 0 |
| TCGA-BP-4970 | MALE | 3.5 | G3 | T1a | LIVING | 432 | 0 | 0 | 0 | 0 | 0 | 0 |
| TCGA-BP-4971 | MALE | 8.1 | G3 | T3a | LIVING | 1487 | 0 | 0 | 0 | 0 | 0 | 0 |
| TCGA-BP-4972 | FEMALE | 4.2 | G3 | T3a | LIVING | 1506 | 0 | 0 | 0 | 0 | 0 | 0 |
| TCGA-BP-4973 | MALE | 9.0 | G3 | T3a | LIVING | 1742 | 0 | 0 | 0 | 0 | 0 | 0 |
| TCGA-BP-4974 | MALE | 11.5 | G4 | T3a | DECEASED | 220 | 0 | 0 | 0 | 0 | 0 | 0 |
| TCGA-BP-4975 | MALE | 5.0 | G3 | T1b | LIVING | 1433 | 0 | 0 | 0 | 0 | 0 | 0 |
| TCGA-BP-4976 | MALE | 4.0 | G3 | T1a | LIVING | 1656 | 0 | 0 | 0 | 0 | 0 | 0 |

| TCGA Sample Code | Gender | Maximum Tumor Dimension (cm) | Tumor Grade | Tumor Stage | Vital Status | Max Follwup | BNC1 cg18952647 | PCDH8 cg20366906 | SCUBE3 cg21604042 | SFRP1 cg13398291 | SFRP1 cg15839448 | SFRP1 cg22418909 |
| --- | --- | --- | --- | --- | --- | --- | --- | --- | --- | --- | --- | --- |
| TCGA-BP-4977 | MALE | 4.5 | G3 | T1b | LIVING | 455 | 0 | 0 | 0 | 0 | 0 | 0 |
| TCGA-BP-4981 | FEMALE | 8.6 | G3 | T3a | DECEASED | 1097 | 0 | 0 | 0 | 0 | 0 | 0 |
| TCGA-BP-4982 | MALE | 4.5 | G3 | T1b | LIVING | 1013 | 0 | 0 | 0 | 0 | 0 | 0 |
| TCGA-BP-4983 | FEMALE | 6.3 | G4 | T3a | LIVING | 1412 | 1 | 1 | 0 | 0 | 0 | 1 |
| TCGA-BP-4985 | MALE | 12.0 | G4 | T3a | DECEASED | 951 | 1 | 1 | 1 | 1 | 0 | 1 |
| TCGA-BP-4986 | MALE | 1.8 | G3 | T1a | LIVING | 785 | 0 | 0 | 0 | 0 | 0 | 0 |
| TCGA-BP-4987 | FEMALE | 4.3 | G2 | T1b | LIVING | 1205 | 0 | 0 | 0 | 1 | 0 | 0 |
| TCGA-BP-4988 | MALE | 2.0 | G2 | T1a | DECEASED | 827 | 0 | 0 | 0 | 0 | 0 | 0 |
| TCGA-BP-4989 | MALE | 6.2 | G3 | T3a | LIVING | 117 | 0 | 1 | 0 | 0 | 0 | 0 |
| TCGA-BP-4991 | MALE | 2.5 | G2 | T1a | LIVING | 1413 | 0 | 0 | 0 | 0 | 0 | 0 |
| TCGA-BP-4992 | MALE | 5.6 | G4 | T1b | LIVING | 500 | 1 | 0 | 0 | 0 | 0 | 0 |
| TCGA-BP-4995 | MALE | 4.6 | G3 | T1b | LIVING | 1371 | 0 | 1 | 0 | 0 | 0 | 0 |
| TCGA-BP-4998 | MALE | 3.5 | G3 | T1a | LIVING | 931 | 0 | 0 | 0 | 0 | 0 | 0 |
| TCGA-BP-4999 | MALE | 3.5 | G2 | T1a | LIVING | 1266 | 0 | 0 | 0 | 0 | 0 | 0 |
| TCGA-BP-5000 | MALE | 5.4 | G3 | T1b | LIVING | 788 | 0 | 1 | 0 | 0 | 0 | 1 |
| TCGA-BP-5001 | FEMALE | 4.1 | G2 | T1b | LIVING | 1177 | 0 | 0 | 0 | 0 | 0 | 0 |
| TCGA-BP-5004 | MALE | 3.0 | G3 | T1a | LIVING | 1126 | 1 | 0 | 0 | 0 | 0 | 0 |
| TCGA-BP-5006 | MALE | 3.0 | G2 | T1a | LIVING | 840 | 0 | 0 | 0 | 0 | 0 | 0 |
| TCGA-BP-5007 | MALE | 10.0 | G2 | T2 | LIVING | 1140 | 0 | 1 | 0 | 0 | 0 | 0 |
| TCGA-BP-5008 | MALE | 2.5 | G2 | T1a | LIVING | 1071 | 0 | 0 | 0 | 0 | 0 | 0 |
| TCGA-BP-5009 | MALE | 5.7 | G3 | T1b | DECEASED | 1102 | 0 | 0 | 1 | 0 | 0 | 0 |
| TCGA-CJ-4634 | FEMALE | 7.0 | G2 | T1b | LIVING | 1820 | 0 | 0 | 0 | 0 | 0 | 0 |
| TCGA-CJ-4635 | MALE | 6.0 | G3 | T1b | LIVING | 1416 | 0 | 0 | 0 | 0 | 0 | 0 |
| TCGA-CJ-4636 | MALE | 7.0 | G3 | T3a | LIVING | 1924 | 0 | 1 | 0 | 0 | 0 | 0 |
| TCGA-CJ-4637 | FEMALE | 9.0 | G4 | T2b | DECEASED | 2227 | 1 | 0 | 1 | 1 | 1 | 1 |
| TCGA-CJ-4638 | FEMALE | 15.0 | G4 | T3a | DECEASED | 431 | 1 | 1 | 0 | 0 | 0 | 1 |
| TCGA-CJ-4639 | FEMALE | 7.7 | G2 | T2 | LIVING | 2308 | 0 | 0 | 0 | 0 | 0 | 0 |
| TCGA-CJ-4640 | MALE | 9.0 | G4 | T3a | LIVING | 1998 | 0 | 0 | 0 | 0 | 0 | 0 |
| TCGA-CJ-4641 | FEMALE | 9.5 | G4 | T3a | DECEASED | 1661 | 0 | 0 | 0 | 1 | 0 | 0 |
| TCGA-CJ-4643 | FEMALE | 9.5 | G3 | T2b | LIVING | 1792 | 0 | 0 | 0 | 0 | 0 | 0 |
| TCGA-CJ-4644 | FEMALE | 10.0 | G3 | T3a | DECEASED | 336 | 0 | 0 | 0 | 0 | 0 | 0 |

| TCGA Sample Code | Gender | Maximum Tumor Dimension (cm) | Tumor Grade | Tumor Stage | Vital Status | Max Follwup | BNC1 cg18952647 | PCDH8 cg20366906 | SCUBE3 cg21604042 | SFRP1 cg13398291 | SFRP1 cg15839448 | SFRP1 cg22418909 |
| --- | --- | --- | --- | --- | --- | --- | --- | --- | --- | --- | --- | --- |
| TCGA-CJ-4868 | MALE | 9.0 | G3 | T3a | DECEASED | 645 | 1 | 1 | 0 | 0 | 0 | 0 |
| TCGA-CJ-4870 | FEMALE | 11.5 | G2 | T3a | LIVING | 1497 | 0 | 0 | 0 | 0 | 0 | 0 |
| TCGA-CJ-4871 | MALE | 12.0 | G4 | T3a | LIVING | 2422 | 0 | 0 | 0 | 0 | 0 | 1 |
| TCGA-CJ-4872 | MALE | 4.5 | G4 | T1b | LIVING | 1435 | 0 | 0 | 0 | 0 | 0 | 0 |
| TCGA-CJ-4873 | FEMALE | 5.0 | G3 | T3a | LIVING | 2258 | 0 | 0 | 0 | 0 | 0 | 0 |
| TCGA-CJ-4874 | FEMALE | 6.0 | G3 | T1b | LIVING | 2283 | 0 | 0 | 1 | 0 | 0 | 0 |
| TCGA-CJ-4875 | MALE | 8.0 | G3 | T3a | LIVING | 2353 | 0 | 0 | 0 | 0 | 0 | 0 |
| TCGA-CJ-4876 | MALE | 10.5 | G3 | T2b | LIVING | 1955 | 0 | 1 | 0 | 0 | 0 | 0 |
| TCGA-CJ-4878 | FEMALE | 4.5 | G2 | T3a | LIVING | 2187 | 0 | 0 | 0 | 0 | 0 | 0 |
| TCGA-CJ-4881 | MALE | 10.0 | G3 | T3a | LIVING | 2013 | 1 | 0 | 0 | 0 | 0 | 0 |
| TCGA-CJ-4884 | FEMALE | 4.5 | G3 | T3a | LIVING | 1759 | 1 | 0 | 0 | 0 | 0 | 0 |
| TCGA-CJ-4885 | MALE | 6.0 | G3 | T3a | LIVING | 2125 | 0 | 0 | 0 | 0 | 0 | 0 |
| TCGA-CJ-4886 | FEMALE | 3.7 | G3 | T1a | LIVING | 1951 | 0 | 0 | 0 | 0 | 0 | 0 |
| TCGA-CJ-4887 | MALE | 4.0 | G3 | T3a | DECEASED | 931 | 0 | 1 | 0 | 0 | 0 | 0 |
| TCGA-CJ-4888 | MALE | 5.8 | G4 | T3a | DECEASED | 1566 | 1 | 0 | 0 | 0 | 0 | 1 |
| TCGA-CJ-4889 | FEMALE | 4.0 | G4 | T1a | LIVING | 1945 | 0 | 0 | 0 | 0 | 0 | 1 |
| TCGA-CJ-4890 | MALE | 14.0 | G4 | T3a | LIVING | 2085 | 0 | 0 | 0 | 0 | 0 | 0 |
| TCGA-CJ-4891 | FEMALE | 8.5 | G4 | T3c | DECEASED | 818 | 0 | 0 | 0 | 0 | 0 | 1 |
| TCGA-CJ-4892 | FEMALE | 5.5 | G2 | T1b | LIVING | 1520 | 0 | 0 | 0 | 0 | 0 | 0 |
| TCGA-CJ-4893 | FEMALE | 5.2 | G3 | T1b | LIVING | 749 | 0 | 0 | 0 | 0 | 0 | 0 |
| TCGA-CJ-4894 | MALE | 11.0 | G3 | T3a | DECEASED | 841 | 0 | 1 | 0 | 0 | 0 | 0 |
| TCGA-CJ-4895 | MALE | 15.9 | G4 | T3a | DECEASED | 1200 | 0 | 0 | 1 | 0 | 0 | 1 |
| TCGA-CJ-4899 | MALE | 4.5 | G2 | T1b | LIVING | 1527 | 0 | 0 | 0 | 0 | 0 | 0 |
| TCGA-CZ-4854 | MALE | 5.7 | G2 | T1b | DECEASED | 1404 | 1 | 1 | 1 | 1 | 0 | 0 |
| TCGA-CZ-4857 | MALE | 9.2 | G3 | T3a | DECEASED | 1432 | 1 | 0 | 1 | 1 | 1 | 1 |
| TCGA-CZ-4858 | MALE | 7.8 | G4 | T2 | LIVING | 2105 | 1 | 1 | 1 | 0 | 0 | 0 |
| TCGA-CZ-4861 | MALE | 8.2 | G2 | T2 | DECEASED | 445 | 1 | 1 | 0 | 0 | 1 | 0 |
| TCGA-CZ-4862 | MALE | 4.7 | G2 | T1b | LIVING | 1843 | 0 | 0 | 0 | 0 | 0 | 0 |

**Table S6: Clinical Information for the HumanMethylation450 BeadChip Cohort and Hypermethylation Status for Significant Genes.**

| TCGA Sample Code | Gender | Maximum Tumor Dimension (cm) | Tumor Grade | Tumor Stage | Vital Status | Max Follwup | BNC1 4 of 13 | GATA5 4 of 14 | GREM1 2 of 3 | SFRP1 3 of 6 | BNC1 cg06523224 | GUCY2D cg04157161 | RASSF5 cg18328206 | SST cg02164046 | ZSCAN18 cg14231297 |
| --- | --- | --- | --- | --- | --- | --- | --- | --- | --- | --- | --- | --- | --- | --- | --- |
| TCGA-A3-3357 | MALE | 7.8 | G3 | T2 | LIVING | 1425 | 1 | 0 | 0 | 1 | 1 | 0 | 0 | 0 | 0 |
| TCGA-A3-3367 | MALE | 4.9 | G3 | T1b | LIVING | 1054 | 0 | 0 | 0 | 0 | 0 | 0 | 0 | 1 | 0 |
| TCGA-A3-3370 | FEMALE | 4.3 | G2 | T1b | LIVING | 776 | 0 | 0 | 0 | 0 | 0 | 0 | 0 | 0 | 0 |
| TCGA-A3-3373 | FEMALE | 6.5 | G3 | T1b | LIVING | 334 | 0 | 0 | 0 | 0 | 0 | 0 | 0 | 0 | 1 |
| TCGA-A3-3376 | MALE | 2.5 | G2 | T1a | LIVING | 1070 | 0 | 0 | 0 | 0 | 0 | 0 | 0 | 0 | 0 |
| TCGA-A3-3385 | FEMALE | 3.5 | G2 | T1a | LIVING | 726 | 0 | 0 | 0 | 0 | 0 | 0 | 0 | 0 | 1 |
| TCGA-B0-4690 | MALE | 12.0 | G3 | T4 | DECEASED | 43 | 0 | 0 | 1 | 1 | 0 | 0 | 1 | 1 | 1 |
| TCGA-B0-4691 | MALE | 10.0 | G3 | T2 | DECEASED | 139 | 1 | 1 | 0 | 1 | 1 | 0 | 1 | 1 | 1 |
| TCGA-B0-4693 | FEMALE | 13.0 | G4 | T3a | DECEASED | 77 | 0 | 0 | 0 | 0 | 0 | 0 | 0 | 0 | 0 |
| TCGA-B0-4694 | MALE | 6.0 | G4 | T3b | DECEASED | 106 | 0 | 0 | 0 | 0 | 0 | 1 | 0 | 0 | 1 |
| TCGA-B0-4697 | FEMALE | 14.0 | G4 | T3b | DECEASED | 577 | 0 | 0 | 0 | 0 | 0 | 0 | 0 | 0 | 0 |
| TCGA-B0-4701 | FEMALE | 11.0 | G3 | T3a | DECEASED | 237 | 0 | 0 | 0 | 0 | 0 | 0 | 0 | 0 | 1 |
| TCGA-B0-4703 | MALE | 10.5 | G4 | T3a | DECEASED | 182 | 0 | 0 | 0 | 0 | 0 | 0 | 0 | 0 | 1 |
| TCGA-B0-4706 | MALE | 7.0 | G4 | T3a | DECEASED | 65 | 0 | 0 | 0 | 0 | 0 | 0 | 0 | 0 | 1 |
| TCGA-B0-4707 | MALE | 10.2 | G4 | T3a | DECEASED | 599 | 0 | 0 | 0 | 0 | 0 | 0 | 0 | 1 | 1 |
| TCGA-B0-4710 | FEMALE | 10.6 | G3 | T3a | LIVING | 95 | 0 | 0 | 0 | 0 | 0 | 1 | 0 | 0 | 1 |
| TCGA-B0-4712 | MALE | 8.5 | G3 | T3a | DECEASED | 1337 | 0 | 1 | 0 | 1 | 0 | 1 | 1 | 1 | 1 |
| TCGA-B0-4713 | FEMALE | 13.5 | G2 | T3b | DECEASED | 201 | 1 | 1 | 1 | 1 | 1 | 0 | 0 | 0 | 1 |
| TCGA-B0-4714 | MALE | 5.0 | G3 | T3b | DECEASED | 98 | 0 | 0 | 0 | 0 | 0 | 0 | 0 | 0 | 1 |
| TCGA-B0-4718 | MALE | 8.0 | G2 | T3a | LIVING | 616 | 1 | 0 | 1 | 1 | 0 | 0 | 1 | 1 | 1 |
| TCGA-BP-4770 | FEMALE | 15.0 | G4 | T4 | DECEASED | 329 | 0 | 0 | 0 | 0 | 1 | 0 | 0 | 0 | 0 |
| TCGA-BP-4782 | FEMALE | 4.2 | G2 | T1a | LIVING | 420 | 0 | 0 | 0 | 0 | 0 | 0 | 0 | 0 | 0 |
| TCGA-BP-4801 | MALE | 3.2 | G2 | T1a | LIVING | 1124 | 0 | 0 | 0 | 0 | 0 | 0 | 0 | 0 | 1 |
| TCGA-B0-4810 | MALE | 11.5 | G3 | T3a | DECEASED | 477 | 0 | 0 | 0 | 0 | 0 | 1 | 0 | 1 | 1 |
| TCGA-B0-4811 | MALE | 6.0 | G3 | T3a | DECEASED | 1417 | 0 | 0 | 0 | 1 | 0 | 0 | 0 | 0 | 0 |
| TCGA-B0-4813 | MALE | 6.5 | G3 | T3b | DECEASED | 18 | 1 | 0 | 0 | 1 | 1 | 1 | 1 | 0 | 1 |
| TCGA-B0-4814 | MALE | 12.5 | G3 | T4 | DECEASED | 167 | 1 | 0 | 0 | 1 | 1 | 1 | 0 | 1 | 1 |
| TCGA-B0-4815 | MALE | 7.9 | G4 | T3a | DECEASED | 1587 | 0 | 1 | 0 | 0 | 0 | 1 | 1 | 0 | 1 |
| TCGA-B0-4816 | MALE | 10.4 | G3 | T2 | DECEASED | 1370 | 0 | 0 | 0 | 0 | 0 | 0 | 0 | 0 | 0 |
| TCGA-B0-4817 | MALE | 4.8 | G3 | T3c | DECEASED | 1019 | 0 | 0 | 0 | 0 | 0 | 1 | 0 | 0 | 1 |
| TCGA-B0-4818 | FEMALE | 9.7 | G3 | T2 | DECEASED | 510 | 0 | 0 | 0 | 0 | 0 | 0 | 0 | 0 | 0 |
| TCGA-B0-4819 | FEMALE | 9.0 | G4 | T3b | DECEASED | 183 | 0 | 0 | 0 | 0 | 0 | 1 | 0 | 1 | 1 |
| TCGA-B0-4822 | MALE | 13.2 | G4 | T2 | DECEASED | 1111 | 0 | 0 | 0 | 1 | 0 | 1 | 0 | 1 | 1 |
| TCGA-B0-4823 | MALE | 3.5 | G2 | T1a | DECEASED | 454 | 0 | 0 | 0 | 0 | 0 | 0 | 0 | 0 | 0 |
| TCGA-B0-4824 | FEMALE | 3.5 | G3 | T1a | DECEASED | 1657 | 0 | 0 | 0 | 0 | 0 | 0 | 0 | 0 | 1 |

| TCGA Sample Code | Gender | Maximum Tumor Dimension (cm) | Tumor Grade | Tumor Stage | Vital Status | Max Follwup | BNC1 4 of 13 | GATA5 4 of 14 | GREM1 2 of 3 | SFRP1 3 of 6 | BNC1 cg06523224 | GUCY2D cg04157161 | RASSF5 cg18328206 | SST cg02164046 | ZSCAN18 cg14231297 |
| --- | --- | --- | --- | --- | --- | --- | --- | --- | --- | --- | --- | --- | --- | --- | --- |
| TCGA-B0-4827 | FEMALE | 11.0 | G4 | T3b | DECEASED | 884 | 0 | 0 | 0 | 1 | 0 | 1 | 0 | 1 | 1 |
| TCGA-B0-4828 | MALE | 9.0 | G3 | T2 | DECEASED | 307 | 0 | 0 | 0 | 0 | 0 | 1 | 0 | 1 | 1 |
| TCGA-B0-4841 | MALE | 7.5 | G3 | T2 | DECEASED | 203 | 0 | 0 | 0 | 0 | 0 | 0 | 0 | 0 | 1 |
| TCGA-B0-4842 | FEMALE | 12.5 | G4 | T3a | DECEASED | 1723 | 0 | 0 | 0 | 0 | 0 | 1 | 0 | 0 | 1 |
| TCGA-B0-4843 | MALE | 6.8 | G3 | T3a | DECEASED | 320 | 0 | 0 | 0 | 0 | 1 | 0 | 0 | 0 | 0 |
| TCGA-B0-4844 | MALE | 8.2 | G3 | T3a | DECEASED | 313 | 1 | 1 | 0 | 0 | 1 | 1 | 1 | 1 | 1 |
| TCGA-B0-4845 | MALE | 5.5 | G2 | T3a | DECEASED | 1985 | 0 | 0 | 0 | 0 | 0 | 0 | 0 | 0 | 1 |
| TCGA-B0-4846 | MALE | 7.6 | G2 | T3a | DECEASED | 1199 | 0 | 0 | 0 | 0 | 0 | 0 | 0 | 0 | 0 |
| TCGA-B0-4847 | MALE | 3.6 | G3 | T3a | DECEASED | 792 | 0 | 0 | 0 | 0 | 0 | 1 | 0 | 0 | 1 |
| TCGA-B0-4848 | MALE | 14.0 | G3 | T3b | DECEASED | 882 | 0 | 0 | 0 | 0 | 0 | 0 | 0 | 0 | 1 |
| TCGA-B0-4849 | MALE | 13.5 | G3 | T3a | DECEASED | 69 | 0 | 0 | 0 | 0 | 0 | 0 | 0 | 0 | 0 |
| TCGA-B0-4852 | FEMALE | 8.8 | G2 | T2 | DECEASED | 1121 | 0 | 0 | 0 | 0 | 1 | 0 | 0 | 0 | 1 |
| TCGA-CZ-4853 | MALE | 3.5 | G2 | T1a | LIVING | 774 | 1 | 0 | 0 | 1 | 0 | 1 | 0 | 0 | 1 |
| TCGA-CZ-4856 | FEMALE | 4.2 | G2 | T1b | LIVING | 18 | 0 | 0 | 0 | 0 | 0 | 1 | 0 | 0 | 0 |
| TCGA-CZ-4859 | FEMALE | 5.0 | G2 | T1 | LIVING | 1787 | 0 | 0 | 0 | 0 | 0 | 0 | 0 | 0 | 0 |
| TCGA-CZ-4863 | FEMALE | 7.5 | G3 | T3b | LIVING | 1927 | 0 | 0 | 0 | 0 | 0 | 1 | 0 | 0 | 0 |
| TCGA-CZ-4865 | FEMALE | 3.0 | G2 | T1a | DECEASED | 165 | 0 | 0 | 0 | 0 | 0 | 0 | 0 | 0 | 0 |
| TCGA-CZ-4866 | FEMALE | 3.3 | G3 | T1 | LIVING | 1867 | 0 | 0 | 0 | 0 | 1 | 0 | 0 | 0 | 1 |
| TCGA-CJ-4882 | MALE | 7.0 | G3 | T3a | LIVING | 1883 | 0 | 0 | 0 | 0 | 0 | 0 | 0 | 0 | 1 |
| TCGA-CJ-4897 | FEMALE | 13.5 | G3 | T3a | LIVING | 1808 | 0 | 0 | 0 | 0 | 0 | 0 | 0 | 0 | 0 |
| TCGA-CJ-4901 | MALE | 14.0 | G3 | T3b | LIVING | 1450 | 0 | 0 | 1 | 0 | 0 | 1 | 0 | 0 | 1 |
| TCGA-CJ-4902 | MALE | 8.0 | G3 | T3a | LIVING | 1519 | 0 | 0 | 0 | 0 | 0 | 1 | 0 | 0 | 1 |
| TCGA-CJ-4903 | MALE | 5.0 | G3 | T1b | LIVING | 1559 | 0 | 0 | 0 | 0 | 0 | 0 | 0 | 0 | 0 |
| TCGA-CJ-4904 | FEMALE | 14.5 | G3 | T3a | LIVING | 1792 | 0 | 0 | 0 | 0 | 0 | 0 | 0 | 0 | 0 |
| TCGA-CJ-4905 | FEMALE | 3.8 | G2 | T1a | LIVING | 1495 | 0 | 0 | 0 | 0 | 0 | 0 | 0 | 0 | 0 |
| TCGA-CJ-4907 | MALE | 6.0 | G3 | T3b | LIVING | 1498 | 0 | 0 | 0 | 0 | 0 | 0 | 0 | 0 | 0 |
| TCGA-CJ-4908 | MALE | 3.9 | G2 | T1a | LIVING | 1530 | 0 | 0 | 0 | 0 | 0 | 0 | 0 | 0 | 0 |
| TCGA-CJ-4912 | MALE | 15.0 | G3 | T2 | LIVING | 1656 | 0 | 0 | 0 | 0 | 0 | 0 | 0 | 0 | 0 |
| TCGA-CJ-4913 | FEMALE | 6.3 | G4 | T3a | DECEASED | 1173 | 0 | 0 | 0 | 0 | 0 | 0 | 0 | 1 | 1 |
| TCGA-CJ-4916 | FEMALE | 10.4 | G3 | T3a | LIVING | 1372 | 0 | 0 | 0 | 0 | 0 | 0 | 0 | 0 | 0 |
| TCGA-CJ-4918 | MALE | 14.0 | G4 | T3a | DECEASED | 92 | 0 | 0 | 0 | 0 | 0 | 0 | 0 | 0 | 0 |
| TCGA-CJ-4920 | FEMALE | 5.5 | G2 | T1b | DECEASED | 139 | 0 | 0 | 0 | 0 | 0 | 0 | 0 | 0 | 1 |
| TCGA-CJ-4923 | FEMALE | 12.0 | G4 | T3a | DECEASED | 572 | 0 | 0 | 0 | 0 | 0 | 1 | 1 | 0 | 1 |
| TCGA-B0-4945 | FEMALE | 4.0 | G2 | T1a | DECEASED | 2145 | 0 | 0 | 0 | 0 | 0 | 0 | 0 | 0 | 0 |
| TCGA-BP-4993 | MALE | 2.5 | G3 | T1a | LIVING | 176 | 0 | 0 | 0 | 0 | 0 | 0 | 0 | 0 | 0 |
| TCGA-BP-5010 | MALE | 12.2 | G4 | T3a | DECEASED | 877 | 0 | 0 | 0 | 1 | 1 | 0 | 0 | 1 | 1 |
| TCGA-B0-5080 | MALE | 9.0 | G3 | T3a | DECEASED | 342 | 0 | 0 | 0 | 0 | 0 | 0 | 0 | 0 | 1 |
| TCGA-B0-5092 | FEMALE | 3.8 | G3 | T1a | DECEASED | 459 | 0 | 0 | 0 | 0 | 0 | 0 | 0 | 1 | 1 |

| TCGA Sample Code | Gender | Maximum Tumor Dimension (cm) | Tumor Grade | Tumor Stage | Vital Status | Max Follwup | BNC1 4 of 13 | GATA5 4 of 14 | GREM1 2 of 3 | SFRP1 3 of 6 | BNC1 cg06523224 | GUCY2D cg04157161 | RASSF5 cg18328206 | SST cg02164046 | ZSCAN18 cg14231297 |
| --- | --- | --- | --- | --- | --- | --- | --- | --- | --- | --- | --- | --- | --- | --- | --- |
| TCGA-B0-5094 | MALE | 8.0 | G2 | T3b | DECEASED | 333 | 1 | 1 | 1 | 1 | 1 | 1 | 1 | 1 | 1 |
| TCGA-B0-5095 | MALE | 10.2 | G3 | T3a | DECEASED | 245 | 0 | 0 | 0 | 0 | 0 | 0 | 0 | 0 | 0 |
| TCGA-B0-5096 | FEMALE | 5.0 | GX | T3a | DECEASED | 67 | 0 | 1 | 0 | 0 | 1 | 1 | 0 | 0 | 1 |
| TCGA-B0-5097 | FEMALE | 7.0 | G2 | T3b | LIVING | 665 | 0 | 0 | 0 | 0 | 0 | 0 | 0 | 0 | 1 |
| TCGA-B0-5099 | FEMALE | 7.0 | G3 | T3b | DECEASED | 485 | 0 | 0 | 0 | 1 | 0 | 0 | 0 | 1 | 1 |
| TCGA-B0-5100 | MALE | 8.0 | G3 | T3a | DECEASED | 1913 | 1 | 0 | 0 | 0 | 1 | 0 | 0 | 0 | 1 |
| TCGA-B0-5102 | FEMALE | 4.0 | G3 | T1 | DECEASED | 2770 | 0 | 0 | 0 | 0 | 0 | 0 | 0 | 0 | 0 |
| TCGA-B0-5104 | FEMALE | 4.5 | G2 | T1 | DECEASED | 2751 | 0 | 0 | 0 | 0 | 0 | 0 | 0 | 0 | 0 |
| TCGA-B0-5106 | MALE | 3.6 | G2 | T1a | DECEASED | 1597 | 0 | 0 | 0 | 0 | 0 | 0 | 0 | 0 | 1 |
| TCGA-B0-5107 | FEMALE | 8.0 | G4 | T2 | DECEASED | 927 | 1 | 0 | 1 | 1 | 1 | 1 | 0 | 1 | 1 |
| TCGA-B0-5108 | MALE | 5.5 | G2 | T3a | LIVING | 911 | 0 | 0 | 0 | 0 | 0 | 1 | 0 | 0 | 1 |
| TCGA-B0-5109 | MALE | 9.5 | G4 | T3b | DECEASED | 586 | 0 | 0 | 0 | 0 | 0 | 1 | 0 | 0 | 0 |
| TCGA-B0-5110 | FEMALE | 3.0 | G2 | T1a | LIVING | 1092 | 0 | 0 | 0 | 0 | 1 | 0 | 0 | 0 | 0 |
| TCGA-B0-5113 | FEMALE | 13.7 | G2 | T3a | LIVING | 359 | 0 | 0 | 0 | 0 | 0 | 0 | 0 | 0 | 0 |
| TCGA-B0-5115 | MALE | 9.0 | G3 | T2 | LIVING | 797 | 0 | 0 | 0 | 0 | 0 | 0 | 0 | 0 | 0 |
| TCGA-B0-5116 | MALE | 10.5 | G3 | T3b | LIVING | 657 | 1 | 0 | 0 | 0 | 1 | 0 | 0 | 0 | 1 |
| TCGA-B0-5119 | FEMALE | 5.7 | G2 | T1b | LIVING | 59 | 0 | 0 | 0 | 0 | 0 | 0 | 0 | 0 | 0 |
| TCGA-B0-5120 | FEMALE | 3.8 | G2 | T1a | LIVING | 493 | 0 | 0 | 0 | 0 | 0 | 0 | 0 | 0 | 1 |
| TCGA-B0-5121 | MALE | 4.5 | G2 | T1b | LIVING | 554 | 0 | 0 | 0 | 0 | 0 | 0 | 0 | 0 | 0 |
| TCGA-BP-5168 | MALE | 4.0 | G2 | T1a | DECEASED | 1463 | 0 | 0 | 0 | 0 | 0 | 0 | 0 | 0 | 0 |
| TCGA-BP-5169 | MALE | 5.0 | G4 | T1b | LIVING | 889 | 0 | 0 | 0 | 0 | 0 | 1 | 0 | 1 | 1 |
| TCGA-BP-5170 | MALE | 3.0 | G2 | T1a | LIVING | 2411 | 0 | 0 | 0 | 0 | 0 | 0 | 0 | 0 | 1 |
| TCGA-BP-5173 | MALE | 2.0 | G2 | T1a | DECEASED | 54 | 0 | 0 | 0 | 0 | 0 | 0 | 0 | 0 | 0 |
| TCGA-BP-5174 | FEMALE | 1.9 | G2 | T1a | LIVING | 2291 | 0 | 0 | 0 | 0 | 0 | 0 | 0 | 0 | 0 |
| TCGA-BP-5175 | MALE | 2.8 | G3 | T1a | LIVING | 932 | 0 | 0 | 0 | 0 | 0 | 1 | 0 | 0 | 1 |
| TCGA-BP-5176 | FEMALE | 2.1 | G2 | T1a | DECEASED | 1590 | 0 | 0 | 0 | 0 | 0 | 0 | 0 | 0 | 0 |
| TCGA-BP-5177 | FEMALE | 4.0 | G3 | T1a | LIVING | 434 | 0 | 0 | 0 | 0 | 0 | 0 | 0 | 0 | 0 |
| TCGA-BP-5178 | MALE | 10.0 | G4 | T3a | DECEASED | 1912 | 1 | 1 | 0 | 1 | 1 | 1 | 1 | 1 | 1 |
| TCGA-BP-5180 | MALE | 4.0 | G2 | T1a | LIVING | 2263 | 0 | 0 | 0 | 0 | 0 | 0 | 0 | 0 | 1 |
| TCGA-BP-5182 | MALE | 3.0 | G3 | T1a | LIVING | 1172 | 0 | 0 | 0 | 0 | 0 | 1 | 0 | 1 | 0 |
| TCGA-BP-5183 | MALE | 5.2 | G3 | T3a | LIVING | 1291 | 0 | 0 | 0 | 0 | 1 | 1 | 0 | 0 | 0 |
| TCGA-BP-5184 | MALE | 3.3 | G3 | T1a | LIVING | 1133 | 0 | 0 | 0 | 0 | 0 | 0 | 0 | 0 | 0 |
| TCGA-BP-5185 | MALE | 3.3 | G3 | T1a | LIVING | 1132 | 0 | 0 | 0 | 0 | 0 | 0 | 0 | 0 | 0 |
| TCGA-BP-5186 | FEMALE | 1.9 | G2 | T1a | LIVING | 693 | 0 | 0 | 0 | 0 | 0 | 0 | 0 | 0 | 0 |
| TCGA-BP-5187 | MALE | 3.5 | G2 | T1a | LIVING | 406 | 0 | 0 | 0 | 0 | 0 | 0 | 0 | 0 | 0 |
| TCGA-BP-5189 | MALE | 4.3 | G4 | T1b | DECEASED | 822 | 0 | 0 | 0 | 0 | 0 | 0 | 0 | 0 | 0 |
| TCGA-BP-5190 | MALE | 4.0 | G3 | T1a | LIVING | 1010 | 0 | 0 | 0 | 0 | 0 | 0 | 0 | 1 | 1 |
| TCGA-BP-5191 | MALE | 3.2 | G2 | T3a | LIVING | 966 | 0 | 0 | 0 | 0 | 0 | 0 | 0 | 1 | 1 |

| TCGA Sample Code | Gender | Maximum Tumor Dimension (cm) | Tumor Grade | Tumor Stage | Vital Status | Max Follwup | BNC1 4 of 13 | GATA5 4 of 14 | GREM1 2 of 3 | SFRP1 3 of 6 | BNC1 cg06523224 | GUCY2D cg04157161 | RASSF5 cg18328206 | SST cg02164046 | ZSCAN18 cg14231297 |
| --- | --- | --- | --- | --- | --- | --- | --- | --- | --- | --- | --- | --- | --- | --- | --- |
| TCGA-BP-5192 | MALE | 2.7 | G2 | T1a | LIVING | 714 | 0 | 0 | 0 | 0 | 0 | 0 | 0 | 0 | 0 |
| TCGA-BP-5194 | MALE | 3.5 | G2 | T1a | LIVING | 796 | 0 | 0 | 0 | 0 | 0 | 0 | 0 | 0 | 0 |
| TCGA-BP-5195 | MALE | 2.4 | G2 | T1a | LIVING | 748 | 0 | 0 | 0 | 0 | 0 | 0 | 0 | 0 | 0 |
| TCGA-BP-5196 | MALE | 2.8 | G2 | T1a | LIVING | 1018 | 0 | 0 | 0 | 0 | 0 | 0 | 0 | 0 | 0 |
| TCGA-BP-5198 | MALE | 6.0 | G3 | T3b | LIVING | 603 | 0 | 0 | 0 | 0 | 1 | 1 | 0 | 1 | 1 |
| TCGA-BP-5199 | MALE | 7.6 | G4 | T2 | LIVING | 1354 | 0 | 0 | 0 | 0 | 0 | 0 | 0 | 1 | 0 |
| TCGA-BP-5200 | MALE | 7.5 | G4 | T2 | LIVING | 1063 | 0 | 0 | 0 | 0 | 0 | 0 | 0 | 0 | 1 |
| TCGA-BP-5201 | MALE | 9.5 | G4 | T3b | LIVING | 951 | 0 | 0 | 0 | 0 | 0 | 0 | 0 | 0 | 1 |
| TCGA-BP-5202 | MALE | 3.0 | G2 | T3a | LIVING | 29 | 0 | 0 | 0 | 0 | 0 | 0 | 0 | 0 | 0 |
| TCGA-B0-5400 | FEMALE | 8.0 | G4 | T3b | LIVING | 1132 | 0 | 0 | 0 | 0 | 0 | 0 | 0 | 0 | 0 |
| TCGA-B0-5402 | MALE | 25.0 | G4 | T4 | LIVING | 456 | 0 | 0 | 0 | 0 | 0 | 0 | 0 | 0 | 1 |
| TCGA-CZ-5451 | MALE | 8.7 | G3 | T2 | LIVING | 1929 | 0 | 0 | 0 | 0 | 0 | 0 | 0 | 0 | 0 |
| TCGA-CZ-5452 | MALE | 14.0 | G2 | T2 | LIVING | 1789 | 0 | 0 | 0 | 0 | 0 | 1 | 0 | 0 | 1 |
| TCGA-CZ-5453 | MALE | 7.2 | G2 | T2 | LIVING | 25 | 0 | 0 | 0 | 0 | 0 | 0 | 0 | 0 | 1 |
| TCGA-CZ-5454 | MALE | 15.1 | G2 | T2 | DECEASED | 722 | 1 | 0 | 0 | 0 | 1 | 1 | 0 | 0 | 1 |
| TCGA-CZ-5455 | MALE | 7.5 | G4 | T3b | DECEASED | 561 | 0 | 0 | 0 | 0 | 0 | 1 | 0 | 0 | 1 |
| TCGA-CZ-5456 | MALE | 12.0 | G3 | T2 | LIVING | 1712 | 1 | 1 | 0 | 1 | 1 | 1 | 1 | 1 | 1 |
| TCGA-CZ-5457 | MALE | 13.5 | G4 | T3a | LIVING | 1682 | 0 | 0 | 0 | 0 | 0 | 0 | 0 | 1 | 1 |
| TCGA-CZ-5458 | MALE | 9.2 | G3 | T3a | LIVING | 1731 | 0 | 0 | 0 | 0 | 0 | 0 | 0 | 0 | 0 |
| TCGA-CZ-5459 | MALE | 4.5 | G3 | T3b | LIVING | 1682 | 0 | 0 | 0 | 0 | 0 | 0 | 0 | 1 | 0 |
| TCGA-CZ-5460 | MALE | 2.4 | G2 | T3b | LIVING | 1430 | 0 | 0 | 0 | 0 | 0 | 0 | 0 | 1 | 1 |
| TCGA-CZ-5461 | MALE | 6.4 | G4 | T1b | DECEASED | 330 | 1 | 1 | 1 | 0 | 1 | 1 | 1 | 1 | 1 |
| TCGA-CZ-5462 | MALE | 4.5 | G3 | T1b | DECEASED | 311 | 0 | 0 | 0 | 0 | 0 | 1 | 0 | 1 | 1 |
| TCGA-CZ-5463 | MALE | 7.2 | G2 | T2 | LIVING | 662 | 0 | 0 | 0 | 0 | 0 | 0 | 0 | 0 | 1 |
| TCGA-CZ-5464 | MALE | 7.5 | G2 | T3b | LIVING | 1740 | 0 | 0 | 0 | 0 | 0 | 0 | 0 | 0 | 1 |
| TCGA-CZ-5465 | FEMALE | 5.6 | G2 | T3b | LIVING | 1446 | 0 | 0 | 0 | 0 | 0 | 0 | 0 | 0 | 1 |
| TCGA-CZ-5466 | MALE | 8.8 | G2 | T3a | LIVING | 685 | 0 | 0 | 0 | 0 | 0 | 1 | 0 | 0 | 1 |
| TCGA-CZ-5467 | FEMALE | 11.0 | G4 | T3a | DECEASED | 73 | 0 | 0 | 0 | 0 | 0 | 0 | 0 | 0 | 0 |
| TCGA-CZ-5468 | MALE | 8.6 | G4 | T3b | DECEASED | 59 | 1 | 1 | 1 | 1 | 1 | 1 | 0 | 1 | 1 |
| TCGA-CZ-5469 | MALE | 11.3 | G2 | T2 | DECEASED | 945 | 1 | 0 | 1 | 0 | 1 | 1 | 1 | 1 | 1 |
| TCGA-CZ-5470 | FEMALE | 7.1 | G3 | T2 | LIVING | 386 | 0 | 0 | 0 | 0 | 1 | 1 | 1 | 0 | 1 |
| TCGA-B0-5710 | MALE | 5.7 | G2 | T1b | LIVING | 1461 | 0 | 0 | 0 | 0 | 0 | 0 | 0 | 0 | 1 |
| TCGA-B0-5711 | MALE | 7.5 | G3 | T3b | LIVING | 2931 | 0 | 0 | 0 | 0 | 0 | 0 | 0 | 0 | 0 |
| TCGA-B0-5713 | FEMALE | 7.2 | G3 | T3b | LIVING | 1864 | 0 | 0 | 0 | 0 | 0 | 0 | 0 | 0 | 0 |
|  |  |  |  |  |  |  |  |  |  |  |  |  |  |  |  |
|  |  |  |  |  |  |  | 18 | 11 | 9 | 19 | 26 | 42 | 14 | 34 | 81 |
|  |  |  |  |  |  |  | 12.41% | 7.59% | 6.21% | 13.10% | 17.93% | 28.97% | 9.66% | 23.45% | 55.86% |

**Table S7: Cox Proportional-Hazard Regression Analysis for the Probes that were Statistically Significantly Associated with Poorer Survival in Patients from the HumanMethylation27 BeadChip Cohort compared with Tumor Stage, Tumor Grade, Tumor Maximum Dimension and Patient Gender.**

|  | **Survival Analysis - Cox Proportional-Hazard Regression** | | | | |
| --- | --- | --- | --- | --- | --- |
|  | **(181 patients with clinical data from the HumanMethylation27 BeadChip Cohort)** | | | | |
| **Gene Name** | **Hypermethylated Probe** | **Survival Indicator** | **p-value** | **Hazard Ratio** | **95% Confidence Interval** |
| BNC1 | cg18952647 | Hypermethylation | 0.0038 | 2.3560 | 1.3227 to 4.1963 |
|  |  | Tumor Stage | 0.0220 | 1.4911 | 1.0642 to 2.0952 |
| SFRP1 | cg22418909 | Tumor Stage | 0.0185 | 1.5097 | 1.1405 to 4.3427 |
|  |  | Hypermethylation | 0.0196 | 2.2255 | 1.0736 to 2.1231 |
| SFRP1 | cg13398291 | Hypermethylation | 0.0092 | 2.4044 | 1.2468 to 4.6368 |
|  |  | Tumor Stage | 0.0174 | 1.5179 | 1.0780 to 2.1373 |
| SFRP1 | cg15839448 | Tumor Stage | 0.0138 | 1.5420 | 1.0943 to 2.1729 |
|  |  | Hypermethylation | 0.0393 | 2.2706 | 1.0452 to 4.3427 |
| PCDH8 | cg20366906 | Hypermethylation | 0.0120 | 2.1271 | 1.1843 to 3.8203 |
|  |  | Tumor Stage | 0.0147 | 1.5378 | 1.0903 to 2.1690 |
| SCUBE3 | cg21604042 | Tumor Stage | 0.0046 | 1.6550 | 1.1700 to 2.3410 |
|  |  | Hypermethylation | 0.0148 | 2.2478 | 1.1757 to 4.2977 |

**Table S8: Kaplan-Meier Survival Curve Analysis for the Single Most Frequently Hypermethylated Probe for each Hypermethylated Gene in the HumanMethylation450 BeadChip Cohort and the Non-Significant Gene Level Results for the HumanMethylation450 BeadChip Cohort.**

|  | **Survival Analysis - Kaplan-Meier Analysis** | | | | | |
| --- | --- | --- | --- | --- | --- | --- |
|  | **(145 patients with clinical data from the HumanMethylation450 BeadChip Cohort)** | | | | | |
| **Gene Name** | **Hypermethylated Probe** | **Percentage Hypermethylation** | **p-value** | **Hazard Ratio** | **95% Confidence Interval** | **FDR corrected p-value** |
| ZSCAN18 | cg14231297 | 55.00% | 0.0028 | 2.1008 | 1.306 to 3.3794 | 0.0028 |
| BNC1 | cg06523224 | 8.13% | 0.0042 | 2.1460 | 1.0897 to 4.2261 | 0.0084 |
| GUCY2D | cg04157161 | 28.13% | 0.0048 | 1.9729 | 1.1298 to 3.4451 | 0.0144 |
| RASSF5 | cg18328206 | 9.38% | 0.0060 | 2.3941 | 0.9689 to 5.9160 | 0.0240 |
| SST | cg02164046 | 24.38% | 0.0071 | 1.9616 | 1.0779 to 3.5697 | 0.0355 |
| UCHL1 | cg07068756 | 11.88% | 0.0161 | 2.0593 | 0.9420 to 4.5016 | 0.0966 |
| GNB4 | cg12872693 | 15.00% | 0.0228 | 1.9463 | 0.9305 to 4.0712 | 0.1596 |
| SCUBE3 | cg00347904 | 6.25% | 0.0247 | 2.5167 | 0.7148 to 8.8609 | 0.1976 |
| DKK3 | cg19867649 | 6.88% | 0.0377 | 2.3568 | 0.6950 to 7.9921 | 0.3393 |
| RPRM | cg26649384 | 16.88% | 0.0440 | 1.7585 | 0.9020 to 3.4284 | 0.4400 |
| HOXC13 | cg07892422 | 7.50% | 0.0466 | 2.1606 | 0.7293 to 6.4015 | 0.5126 |
| WIF1 | cg26397188 | 17.50% | 0.0470 | 1.7674 | 0.8873 to 3.5204 | 0.5640 |
|  |  |  |  |  |  |  |
|  |  |  |  |  |  |  |
| **Gene Name** | **Hypermethylated Probes Assessed** | **Percentage Hypermethylation** | **p-value** | **Hazard Ratio** | **95% Confidence Interval** | **FDR corrected p-value** |
| PCDH8 | 4 of 11 | 36.55% | 0.0638 | 1.5573 | 0.9369 to 2.5888 | - |
| SST | 2 of 3 | 15.86% | 0.0864 | 1.6548 | 0.8262 to 3.3144 | - |
| DLEC1 | 2 of 3 | 10.34% | 0.1343 | 1.6585 | 0.7350 to 3.7423 | - |
| DKK1 | 3 of 5 | 8.28% | 0.2117 | 1.5914 | 0.6536 to 3.8751 | - |
| OVOL1 | Both | 9.66% | 0.2866 | 1.4581 | 0.6475 to 3.2833 | - |
| SLC34A2 | 4 of 7 | 24.83% | 0.3117 | 1.3139 | 0.7368 to 2.3432 | - |
| ROBO1 | 2 of 3 | 9.66% | 0.3158 | 1.4527 | 0.6168 to 3.4210 | - |
| ATP5G2 | 2 of 4 | 33.79% | 0.3713 | 1.2528 | 0.7470 to 2.1011 | - |
| ZSCAN18 | 4 of 7 | 17.24% | 0.6689 | 1.1401 | 0.6071 to 2.1413 | - |
| QPCT | 3 of 5 | 11.72% | 0.7517 | 0.8886 | 0.4388 to 1.7997 | - |
| GSTP1 | 2 of 4 | 14.48% | 0.7658 | 1.1026 | 0.5656 to 2.1494 | - |
| EPB41L3 | 4 of 11 | 14.48% | 0.7772 | 1.0901 | 0.5857 to 2.0286 | - |
| PDLIM4 | Both | 13.79% | 0.8360 | 0.9254 | 0.4521 to 1.8943 | - |
| TMPRSS2 | 2 of 4 | 12.41% | 0.8694 | 0.9404 | 0.4574 to 1.9333 | - |
| UCHL1 | 3 of 5 | 5.52% | 0.9041 | 1.0638 | 0.3767 to 3.0045 | - |

**Table S9: Cox Proportional-Hazard Regression Analysis for the Probes or Genes that were Statistically Significantly Associated with Poorer Survival in Patients from the HumanMethylation450 BeadChip Cohort compared with Tumor Stage, Tumor Grade, Tumor Maximum Dimension and Patient Gender.**

|  | **Survival Analysis - Cox Proportional-Hazard Regression** | | | | |
| --- | --- | --- | --- | --- | --- |
|  | **(145 patients with clinical data from the HumanMethylation450 BeadChip Cohort)** | | | | |
| **Gene Name** | **Hypermethylated Probe(s)** | **Survival Indicator** | **p-value** | **Hazard Ratio** | **95% Confidence Interval** |
| ZSCAN18 | cg14231297 | Tumor Grade | 0.0191 | 1.5750 | 1.0792 to 2.2985 |
|  |  | Hypermethylation | 0.0280 | 1.8653 | 1.0727 to 3.2434 |
| BNC1 | cg06523224 | Tumor Grade | 0.0164 | 1.6173 | 1.0943 to 2.3902 |
|  |  | Hypermethylation | 0.0503 | 1.7579 | 1.0029 to 3.0836 |
| SFRP1 | ≥ 3 out of 6 | Tumor Grade | 0.0326 | 1.5453 | 1.0389 to 2.2986 |
|  |  | Hypermethylation | 0.0357 | 1.9159 | 1.0476 to 3.5039 |
| GREM1 | ≥ 2 out of 3 | Tumor Grade | 0.0054 | 1.7095 | 1.1745 to 2.4886 |
|  |  | Hypermethylation | 0.0073 | 3.0509 | 1.3567 to 6.8607 |
| BNC1 | ≥ 4 out of 13 | Tumor Grade | 0.0123 | 1.6464 | 1.1149 to 2.4018 |
|  |  | Hypermethylation | 0.0334 | 1.9931 | 1.0591 to 3.7509 |
